# Effects of Pharmacologic and Nonpharmacologic Interventions for the Management of Sleep Problems in People With Fibromyalgia: Systematic Review and Network Meta‐Analysis of Randomized Controlled Trials

**DOI:** 10.1002/acr.25505

**Published:** 2025-03-26

**Authors:** Jemma Hudson, Mari Imamura, Clare Robertson, Daniel Whibley, Lorna Aucott, Katie Gillies, Paul Manson, Debra Dulake, Abhishek Abhishek, Nicole K. Y. Tang, Gary J. Macfarlane, Miriam Brazzelli

**Affiliations:** ^1^ University of Aberdeen Aberdeen United Kingdom; ^2^ University of Aberdeen, Aberdeen, United Kingdom, and University of Michigan Ann Arbor; ^3^ University of Nottingham Nottingham United Kingdom; ^4^ University of Warwick Coventry United Kingdom

## Abstract

**Objective:**

Fibromyalgia is a chronic condition characterized by widespread musculoskeletal pain and fatigue. Almost everyone with fibromyalgia has sleep problems. We aimed to evaluate the effectiveness and safety of current interventions for the management of fibromyalgia‐related sleep problems.

**Methods:**

Major electronic databases were searched in November 2021. We focused on randomized controlled trials assessing pharmacologic and/or nonpharmacologic interventions in adults and children and identified 168 studies for inclusion. We assessed the methodologic quality of included studies using the Cochrane Risk‐of‐Bias tool. Our primary outcome of interest was sleep quality assessed using validated patient‐reported outcome measures.

**Results:**

Results from primary studies were analyzed using network meta‐analyses (NMA). The NMA for sleep quality included 65 studies evaluating 35 treatment categories (8,247 participants). Most studies were at high overall risk of bias. Compared with placebo or sham treatments, there was some evidence that exercise (specifically land‐based aerobic exercise training in combination with flexibility training [standardized mean difference (SMD) −4.69, 95% credible interval (Crl) −8.14 to −1.28] and aquatic‐based aerobic exercise training [SMD −2.63, 95% Crl −4.74 to −0.58]) may improve sleep. There was also a suggestion that land‐based strengthening exercise, psychological and behavioral therapy with a focus on sleep, electrotherapy, weight loss, dental splints, antipsychotics, and tricyclics may have a modest effect on sleep.

**Conclusion:**

There is a low level of certainty surrounding the effectiveness of interventions for the management of sleep problems in people with fibromyalgia, but some forms of exercise training appear more likely to provide an improvement in sleep quality.

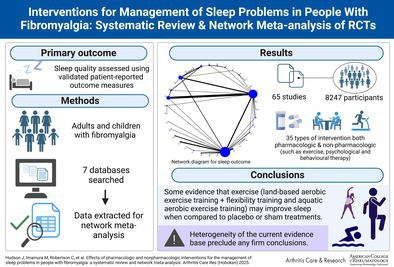

## INTRODUCTION

Fibromyalgia is a complex, heterogeneous condition^1^ that affects 2% to 3% of the global population.^2^ In the absence of a cure, a range of treatments are offered to alleviate symptoms. Most people with fibromyalgia complain about sleep problems.^3^, ^4^ Fibromyalgia‐related sleep problems are poorly managed, and after an initial diagnosis, people continue to seek help to improve their sleep for many years.^5^

**SIGNIFICANCE & INNOVATIONS**

- This systematic review and network meta‐analysis provides a comprehensive and up‐to‐date synthesis of randomized clinical trials investigating pharmacologic and nonpharmacologic interventions for fibromyalgia‐related sleep problems.
- A wide range of interventions, especially nonpharmacologic interventions, have been tested in fibromyalgia trials with very low‐to‐moderate certainty regarding effectiveness.
- Our results indicate that engaging in some forms of exercise—such as land‐based aerobic exercise training in combination with flexibility training and aquatic‐based aerobic exercise training—may improve sleep quality in people with fibromyalgia.
- Certain pharmacologic interventions may also be effective in improving sleep but not without side effects.

The 2015 European guidelines for the management of fibromyalgia considered sleep as a key outcome of interest.^1^ Although general recommendations were made for interventions to manage sleep, these were graded as “weak” due to a paucity of published evidence at that time. Additionally, sleep was not the primary focus of the guidelines. Previously published evidence reviews informed the National Institute of Health and Care Excellence (NICE) draft guidelines for the management of chronic pain; however, these cluster a wide range of conditions (including osteoarthritis, mechanical back pain, and fibromyalgia) and do not have a specific focus on sleep.^6^ Given the number of published randomized controlled trials (RCTs) in this field since 2015, the objective of this study was to undertake a comprehensive evidence synthesis and network meta‐analysis (NMA) to assess the clinical effectiveness and adverse events of pharmacologic and nonpharmacologic treatments for the management of fibromyalgia‐related sleep problems.

## PATIENTS AND METHODS

This systematic review and NMA was conducted in line with the recommendations of the Cochrane Handbook for Systematic Reviews of Interventions^7^ and in adherence with the Preferred Reporting Items for Systematic Reviews and Meta‐Analyses (PRISMA) guidelines.^8^ The study protocol was registered in the PROSPERO database (CRD42021296922).

### Search strategy

Comprehensive search strategies were developed by an information scientist with input from our expert advisers to identify RCTs in patients with fibromyalgia with sleep as an outcome. The searches were not restricted by publication date or language, and we used the Cochrane Highly Sensitive Search Strategy filter for identifying RCTs. The following databases were searched in November 2021: Ovid MEDLINE, Embase, PyscINFO, Allied and Complementary Medicine Database, EBSCO CINAHL, Clarivate Science Citation Index, and the Cochrane Central Register of Controlled Trials. Reference lists of systematic reviews and included studies were checked to identify additional potentially relevant reports. Details of the search strategies are reported in Supplementary Material S2.

### Study selection

Studies were eligible for inclusion if they were RCTs with a parallel‐group, cross‐over, or cluster design comparing pharmacologic and nonpharmacologic interventions to treat sleep problems in adults and children with fibromyalgia versus usual care, placebo, no treatment (including waiting list), or another active intervention. Studies that compared two or more regimens of the same treatment (eg, varying doses of the same drug) were excluded if a placebo or another intervention group was not considered. Two reviewers (MI and CR) independently screened a sample of 100 titles and abstracts at the beginning of the study selection process and compared results to ensure consistency. The remaining citations were divided into two sets and allocated to the same two reviewers. All potentially relevant articles were retrieved in full and assessed by one reviewer for inclusion with a second reviewer checking all articles that were labeled unclear and 10% of the excluded articles. Disagreements were resolved by discussion between reviewers.

### Data extraction

The primary outcomes of interest were sleep quality (patient's experience of sleep and perceived sleep quality) and adverse events. Secondary outcomes were sleep efficiency (calculated as total sleep time/total time in bed × 100%), duration of sleep and/or total sleep time, and disease‐specific quality of life (QoL). For sleep quality, we identified through an update of a previously published systematic review^9^ five patient‐reported outcome measures (PROMs) validated in people with fibromyalgia. These outcome measures were the Pittsburgh Sleep Quality Index (PSQI),^10^ the Medical Outcomes Study Sleep Scale (MOS‐SS),^11^ the Jenkins Sleep Scale (JSS),^12^ the Fibromyalgia Sleep Diary (FMSD),^13^ and the Sleep Quality Numeric Rating Scale (SQ‐NRS).^14^ Single‐item numerical rating scales (NRS) or visual analog scales (VAS) broadly measuring a similar sleep quality construct to that of the SQ‐NRS were also considered proxy measures. In the absence of an accepted fibromyalgia‐specific QoL tool, we used the Fibromyalgia Impact Questionnaire (FIQ)^15^ and the Short Form 36 Health Survey (SF‐36) physical component summary (PCS) and mental component summary (MCS) as a proxy for disease‐specific measures.^16^ Information on how sleep duration and efficiency were assessed (eg, self‐reported or objectively measured) was not consistently reported across included studies. We recorded adverse events that occurred in ≥10% of participants in included studies and serious adverse events. Outcomes were collected at the end of the intervention period or the first assessment point thereafter.

For each study, we extracted information on study design, participants, interventions, and outcome measures. The risk of bias (RoB) in included studies was assessed using the revised Cochrane RoB tool and associated full guidance document.^17^ Two reviewers (CR and MI) conducted dual independent data extraction and RoB assessment from 10% of the included studies using a bespoke pro forma. Single data extraction and RoB assessment were undertaken by the two reviewers for the remaining studies, with one reviewer checking the information extracted by the other reviewer for consistency. Any discrepancy was resolved by discussion between reviewers or consultation with a third reviewer (MB). Two reviewers working together evaluated the certainty of the evidence included in the NMA, using the Confidence in NMA (CINeMA) approach,^18^ which is broadly based on the Grading of Recommendations Assessment, Development and Evaluation framework.^19^

### Data analysis

We pooled all sleep quality PROMs together to form an overarching sleep quality outcome. We also analyzed each individual outcome through sensitivity analyses (results not presented).

For studies reporting more than one sleep quality outcome, we specified a hierarchy based on the most frequently reported outcome across included studies. The adopted hierarchical order was as follows: PSQI, MOS‐SS, JSS, FMSD, and SQ‐NRS. Because a mixture of “change from baseline” and “final score” were available from the included studies, we converted the final score to “change from baseline” when baseline values were available. For the imputation of the change from baseline SD, we used a correlation coefficient as per the recommendation of the Cochrane Handbook for Systematic Reviews of Intervention.^7^ Because we had no available data to calculate the correlation coefficient, we chose a 0.5 value and performed a sensitivity analysis assuming a correlation coefficient of 0.8 to assess whether the results changed. The effect size calculated was the standardized mean difference (SMD), which divides the difference in mean between interventions by the estimated pooled between‐person SD for that trial. Because some studies had small sample sizes, we used the Hedges (adjusted) G method.^20^ Effect sizes reported were either SMD for the sleep quality outcome and mean differences (MD) for the remaining outcomes, with 95% confidence intervals or credible intervals (Crl).

Whenever possible, we performed pairwise and NMAs of all relevant outcome variables. For each pairwise meta‐analysis, a random‐effects model was used to compare the direct evidence, with the percentage of variation across studies due to heterogeneity being assessed by *I* ^*2*^ statistic.

For each outcome, an NMA was performed to combine both direct and indirect evidence using a Bayesian framework, according to guidance from the NICE Decision Support Unit in the United Kingdom and reported in adherence with the PRISMA for NMAs.^8^ Random‐effects models with a normal likelihood were used because all our outcomes were continuous. Convergence was assessed using history, autocorrelation, and Brooks‐Gelman Rubin plots. A sensitivity analysis, which removed pharmacologic interventions from the main analysis, showed only minimal differences (results not presented). Consistency was evaluated by examining the agreement between direct and indirect evidence in all closed loops. To explore the presence of inconsistency for any treatment contrast in the network, we performed a node‐splitting analysis. We also estimated the ranking probabilities of the different interventions using the surface under the cumulative ranking (SUCRA) curve, which is a numeric presentation of the likelihood that an intervention is successful, as well as rankograms. The network diagrams and the node‐splitting analysis were performed in Stata 17^21^ whereas all remaining analysis was completed using the WinBUGS (Medical Research Council Biostatistics Unit).^22^

### Data availability

The main technical data of this evidence synthesis are presented in the text or contained within tables, figures, and supplemental material. Additional results not presented in this manuscript, data used to analyze secondary outcomes, and additional raw data extracted from the included studies can be obtained from the corresponding author on request.

## RESULTS

**Figure 1:**
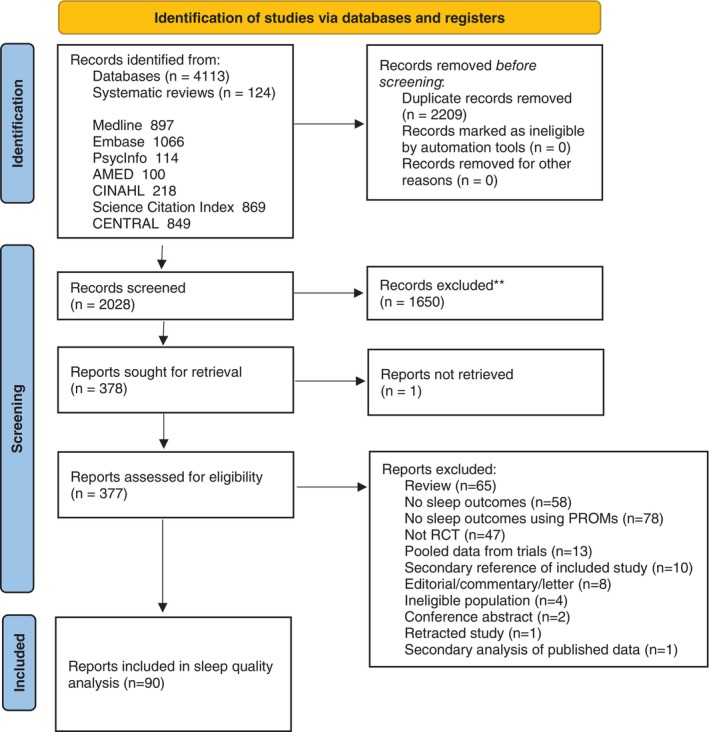
PRISMA flow diagram for identification of the quantitative studies. For more information, visit: http://www.prisma‐statement.org/. *Source*: Page MJ, McKenzie JE, Bossuyt PM, et al. The PRISMA 2020 statement: an updated guideline for reporting systematic reviews. *BMJ* 2021;372:n71. AMED, Allied and Complementary Medicine Database; CENTRAL, Cochrane Central Register of Controlled Trials; CINAHL, Cumulative Index to Nursing and Allied Health Literature; PRISMA, Preferred Reporting Items for Systematic Reviews and Meta‐Analyses; PROM, patient‐reported outcome measure; RCT, randomized controlled trial.

The literature searches identified 4,113 citations, and 378 full‐text records were assessed for eligibility. Of these, 90 (total n = 12,082 participants) assessed sleep quality using one of the PROMs validated in people with fibromyalgia (ie, PSQI, MOS‐SS, JSS, FMSD, and SQ‐NRS) and were included in the NMA (Figure 1).

Of the 90 studies, all participants were adults, 94% were women, with an average age ranging from 35.1 to 57.7 years. According to the information from 30 studies that reported ethnicity, most participants were “White” or “Caucasian.” Further details of study characteristics are presented in Supplementary Material S3. Across studies, a total of 97 active treatments, alone or in combination, were assessed. Most (78%) were nonpharmacologic treatments. These treatments were grouped into 45 categories (34 nonpharmacologic and 11 pharmacologic) according to their characteristics and mode of action.

### RoB of included studies

**Figure 2:**
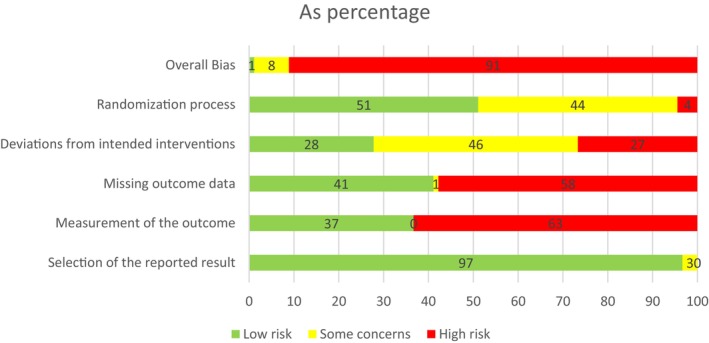
Summary of RoB assessment of the included studies. RoB, risk of bias.

The summary of the RoB assessment for studies included in the NMA is shown in Figure 2. Eighty‐two (91.1%) were judged as high RoB in at least one domain; therefore, the overall RoB was judged as high. Seven studies (7.8%) were given an overall judgment of “some concerns”^23^, ^24^, ^25^, ^26^, ^27^, ^28^, ^29^, and one study (1.1%) had an overall judgment of low risk.

### Sleep quality outcome

**Figure 3:**
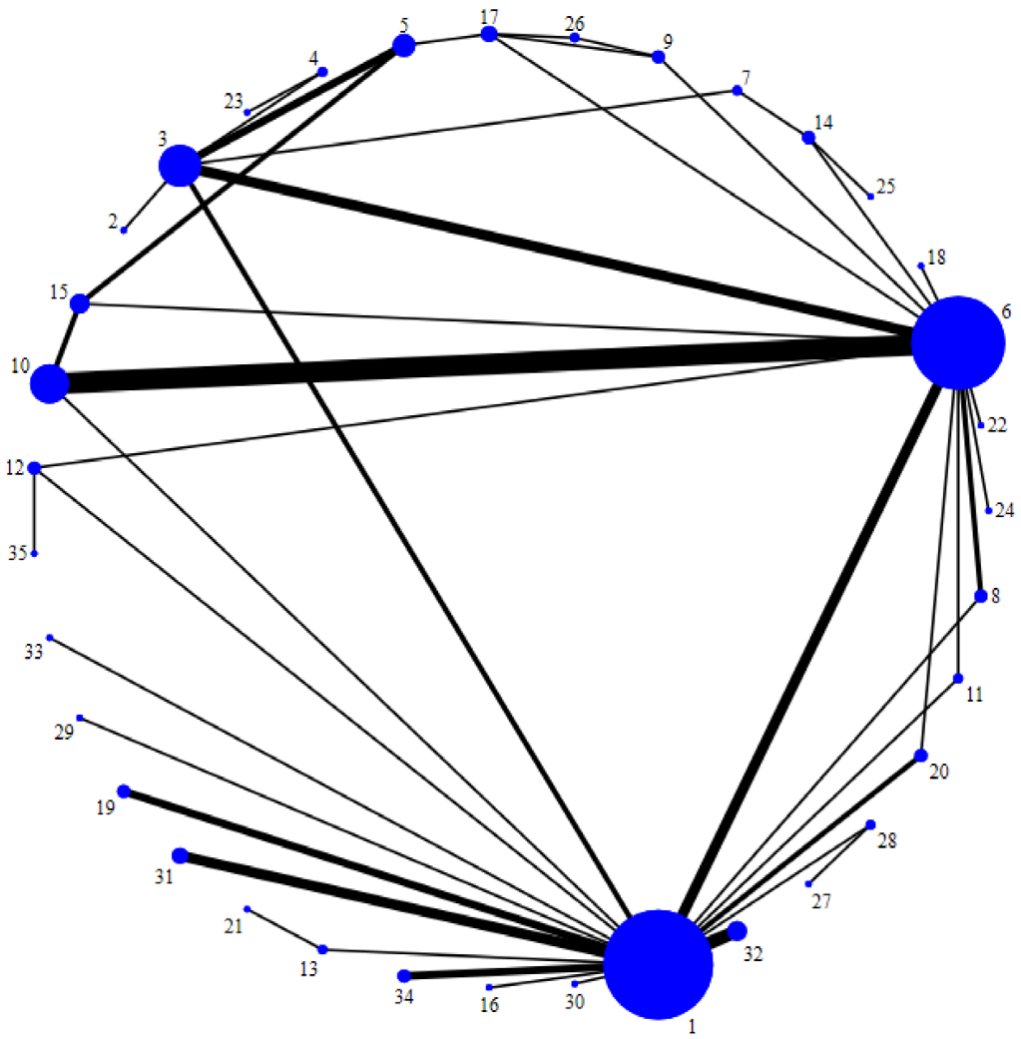
Network diagram for sleep outcome. 1, placebo or sham; 2, education and LD flexibility exercise; 3, LD mind‐body exercise; 4, LD aerobic exercise; 5, education; 6, usual care; 7, AQ aerobic exercise; 8, nutrition; 9, balneotherapy; 10, generic PT/BT; 11, manual therapy; 12, relaxation; 13, electrotherapy; 14, LD flexibility exercise; 15, PT/BT targeted to sleep; 16, AQ mind‐body exercise; 17, AQ mixed exercise; 18, weight loss; 19, neuromodulation; 20, nonmainstream practice; 21, dental splint; 22, hyperbaric oxygen therapy; 23, LD aerobic exercise and LD flexibility exercise; 24, multidisciplinary; 25, LD flexibility exercise and manual therapy; 26, balneotherapy and AQ mixed exercise; 27, tricyclics; 28, antipsychotics; 29, endogenous hormones; 30, antioxidant; 31, SRIs; 32, gabapentinoid; 33, analgesic; 34, CNS depressants; and 35, LD strengthening exercise. Circle size represents the number of randomized participants; line width represents the number of direct comparisons. AQ, aquatic; CNS, central nervous system; LD, land‐based; PT/BT, psychological or behavioral therapy; SRI, serotonin reuptake inhibitor. Color figure can be viewed in the online issue, which is available at http://onlinelibrary.wiley.com/doi/10.1002/acr.25505/abstract.

Sixty‐five studies (72%) assessed sleep quality and were included in the NMA (n = 8,247 participants, 35 treatments). Studies were excluded from the network if they did not provide all required data (13 studies),^27^, ^30^, ^31^, ^32^, ^33^, ^34^, ^35^, ^36^, ^37^, ^38^, ^39^, ^40^, ^41^ were disconnected from the main network (4 studies),^23^, ^25^, ^26^, ^42^ evaluated an intervention and a comparator that belonged to the same category (5 studies),^43^, ^44^, ^45^, ^46^, ^47^ or did not clarify whether the outcome was an index or subscale of a validated scale (2 studies);^28^, ^48^ 1 study^49^ was removed because of data outliers (mean and SD were considerably different). The network comprises 39 studies providing PSQI outcome data, 13 providing MOS‐SS data, 6 JSS data, 3 VAS data, 2 SQ‐NRS, and 1 study each providing FMSD and NRS data. Figure 3 shows the network plot for eligible comparisons for sleep quality. Most interventions were compared with either placebo or sham or usual care. Of the 35 interventions, most were nonpharmacologic (n = 26). For cross‐over trials, we only used data from the first phase before the cross‐over.

Table 1 shows the NMA results for the interventions versus placebo or sham (see Supplementary Material S4 for all other comparisons). The sensitivity analysis, in which we assumed a higher correlation for calculating SD for those studies that did not provide a change from baseline score, showed similar results.

**Table 1:** Primary outcome (sleep quality): NMA evidence plus GRADE*

Interventions	SMD (95% Crl)	GRADE
Education + LD flexibility exercise	0.61 (−1.90 to 3.15)	Very low^a^ ^,^ ^b^ ^,^ ^e^
LD mind‐body exercise	−0.20 (−1.27 to 0.89)	Low^a^ ^,^ ^d^
LD aerobic exercise	−0.14 (−2.63 to 2.30)	Very low^a^ ^,^ ^b^ ^,^ ^e^
Education	0.08 (−1.32 to 1.47)	Very low^a^ ^,^ ^b^ ^,^ ^e^
Usual care	−0.17 (−1.07 to 0.72)	Low^a^ ^,^ ^c^
AQ aerobic exercise	−2.63 (−4.74 to −0.58)	Low^a^ ^,^ ^e^
Nutrition	−0.16 (−1.81 to 1.49)	Low^a^ ^,^ ^b^
Balneotherapy	−0.60 (−2.55 to 1.35)	Very low^a^ ^,^ ^d^ ^,^ ^e^
Generic PT/BT	−0.44 (−1.57 to 0.66)	Low^a^ ^,^ ^d^
Manual therapy	−0.52 (−2.18 to 1.15)	Low^a^ ^,^ ^d^
Relaxation	−0.62 (−2.57 to 1.34)	Low^a^ ^,^ ^d^
Electrotherapy	−0.98 (−3.28 to 1.34)	Very low^a^ ^,^ ^d^ ^,^ ^e^
LD Flexibility exercise	0.49 (−1.56 to 2.56)	Very low^a^ ^,^ ^b^ ^,^ ^e^
PT/BT sleep	−0.89 (−2.39 to 0.61)	Very low^a^ ^,^ ^c^ ^,^ ^e^
AQ mind‐body exercise	4.26 (1.76–6.76)	Low^a^ ^,^ ^e^
AQ Mixed exercise	−0.19 (−1.91 to 1.52)	Very low^a^ ^,^ ^b^ ^,^ ^e^
Weight loss	−1.15 (−3.55 to 1.27)	Very low^a^ ^,^ ^d^ ^,^ ^e^
Neuromodulation	−0.25 (−1.55 to 1.05)	Very low^a^ ^,^ ^d^ ^,^ ^e^
Nonmainstream practice	−1.15 (−2.66 to 0.33)	Moderate^a^
Dental splint	−1.62 (−4.86 to 1.65)	Low^a^ ^,^ ^e^
HBOT	−4.51 (−7.44 to −1.56)	Low^a^ ^,^ ^e^
LD aerobic exercise + LD flexibility exercise	−4.69 (−8.14 to −1.28)	Low^a^ ^,^ ^e^
Multidisciplinary	1.79 (−0.61 to 4.20)	Low^a^ ^,^ ^e^
LD Flexibility exercise + manual therapy	0.78 (−2.30 to 3.83)	Very low^a^ ^,^ ^b^ ^,^ ^e^
Balneotherapy + AQ mixed exercise	0.38 (−2.19 to 2.89)	Very low^a^ ^,^ ^b^ ^,^ ^e^
Tricyclics	−1.26 (−4.47 to 1.93)	Very low^a^ ^,^ ^b^ ^,^ ^e^
Antipsychotics	−1.28 (−3.56 to 0.97)	Very low^a^ ^,^ ^d^ ^,^ ^e^
Endogenous hormones	0.24 (−2.06 to 2.53)	Low^a^ ^,^ ^e^
Antioxidant	−0.29 (−2.61 to 2.06)	Low^b^ ^,^ ^e^
SRI	−0.02 (−1.13 to 1.10)	Very low^a^ ^,^ ^b^ ^,^ ^e^
Gabapentinoid	−0.42 (−1.41 to 0.56)	Very low^a^ ^,^ ^b^ ^,^ ^e^
Analgesic	−0.24 (−2.46 to 1.94)	Very low^a^ ^,^ ^d^ ^,^ ^e^
CNS depressants	−0.19 (−1.50 to 1.13)	Very low^a^ ^,^ ^b^ ^,^ ^e^
LD strengthening exercise	−0.95 (−3.89 to 2.04)	Very low^a^ ^,^ ^d^ ^,^ ^e^

* Negative values indicate a better outcome, whereas positive values indicate a worse outcome. AQ, aquatic; Crl, credible interval; CNS, central nervous system; GRADE, Grading of Recommendations Assessment, Development and Evaluation; HBOT, hyperbaric oxygen therapy; LD, land‐based; NMA, network meta‐analysis; PT/BT, psychological or behavioral therapy; PT/BT sleep, PT/BT targeted to sleep; SMD, standardized mean difference; SRI, serotonin reuptake inhibitor.

^a^ Downgraded by one level due to major concerns on the within‐study bias.

^b^ Downgraded by one level due to major concerns on imprecision.

^c^ Downgraded by one level due to major concerns on heterogeneity.

^d^ Downgraded by one level due to some concerns on both imprecision and heterogeneity.

^e^ Downgraded by one level due to major concerns on incoherence.

Compared with placebo or sham treatment (n = 2,087), there was evidence of a beneficial effect on sleep quality for aquatic‐based aerobic exercise training (n = 59; SMD −2.63, 95% Crl −4.74 to −0.58) and land‐based aerobic exercise training in combination with flexibility exercise training (n = 32; SMD −4.69, 95% Crl −8.14 to −1.28). There was also a suggestion of a modest effect on sleep for land‐based strengthening exercise training (n = 56; SMD −0.95, 95% Crl −3.89 to 2.04), psychological or behavioral therapy (PT/BT) with a focus on sleep (PT/BT sleep; n = 94; SMD −0.89, 95% Crl −2.39 to 0.61), weight loss (n = 41; SMD −1.15, 95% Crl −3.55 to 1.27), electrotherapy (n = 20; SMD −0.98, 95% Crl −3.28 to 1.34), dental splint (n = 29; SMD −1.62, 95% Crl −4.862 to 1.65), tricyclics (n = 43; SMD −1.26, Crl −4.47 to 1.93), and antipsychotics (n = 53; SMD −1.28, Crl −3.56 to 0.97); however, this could not be confirmed with certainty because of the width of the Crl, and our certainty in the current evidence was generally low. We found a positive effect for hyperbaric oxygen therapy (n = 9; SMD −4.51, 95% Crl −7.44 to −1.56) compared with placebo or sham, but this estimate was derived from indirect evidence and based on the assessment of only nine participants in the intervention group. For most other pharmacologic and nonpharmacologic interventions, there was no clear evidence of an improvement in sleep quality, and the certainty of evidence is low to very low.

### QoL outcome: FIQ

Fifty‐two (n = 7,127 participants, 35 interventions) of the 56 studies that reported FIQ were included in the NMA (four studies were excluded because they did not form part of the main network). Results are presented in Table 2 and Supplementary Material 4. Improvements in FIQ were observed for land‐based aerobic exercise in combination with mixed flexibility exercise training (n = 32; MD −19.91, 95% Crl −34.89 to −4.94), multidisciplinary training (n = 81; MD −17.31, 95% Crl −28.38 to −6.29), land‐based mind‐body exercise training (n = 420; MD −16.18, 95% Crl −22.72 to −9.73), generic psychological or behavioral therapy (PT/BT generic) with relaxation (n = 29; MD −12.07, 95% Crl −20.75 to −3.35), PT/BT sleep (n = 77; MD −11.68, 95% Crl −20.34 to −3.11), and generic PT/BT (n = 145; MD −6.23, 95% Crl −12.02 to −0.62) compared with placebo or sham. Positive effects were observed for participants receiving antioxidants (n = 12; MD −17.75, 95% Crl −34.91 to −0.61), iron replacement (n = 38; MD −15.10, 95% Crl −30.41 to −0.06), serotonin reuptake inhibitors (SRI) (n = 573; MD −9.85, 95% Crl −15.80 to −3.80), and central nervous system (CNS) depressants (n= 881; MD −8.83, 95% Crl −14.77 to −2.74). In general, the magnitude of effects varied across interventions. A large positive effect was also observed after hyperbaric oxygen therapy (n = 9; MD −26.29, 95% Crl −37.56 to −15.15); however, as before, we question the reliability of this estimate due to the very small sample (nine patients in the intervention group) and lack of a proper comparator intervention.

**Table 2:** Secondary outcomes (quality of life)*

	FIQ^a^	SF‐36 MCS^b^	SF‐36 PCS^b^
Interventions	MD (95% Crl)	MD (95% Crl)	MD (95% Crl)
Education + LD flexibility exercise	2.14 (−11.61 to 15.64)	1.27 (−7.30 to 10.30)	0.59 (−5.42 to 7.95)
LD mind‐body exercise	−16.18 (−22.72 to −9.73)	7.27 (1.11–13.94)	7.61 (3.56–13.06)
LD aerobic exercise	−9.23 (−21.46 to 3.07)	4.23 (−3.49 to 12.33)	6.17 (1.05–12.81)
Education	−4.79 (−12.87 to 3.21)	10.32 (2.06–19.35)	3.29 (−3.10 to 11.04)
AQ flexibility exercise	5.58 (−8.00 to 19.30)	NA	NA
Usual care	0.79 (−3.72 to 5.15)	−0.46 (−5.22 to 4.36)	0.68 (−2.48 to 3.87)
AQ aerobic exercise	−8.92 (−23.46 to 5.59)	NA	NA
Nutrition	−5.06 (−12.99 to 2.86)	−7.96 (−14.83 to −1.11)	0.82 (−4.02 to 5.43)
Balneotherapy	−5.58 (−18.68 to 7.68)	NA	NA
Generic PT/BT	−6.23 (−12.02 to −0.62)	NA	NA
Manual therapy	−9.22 (−20.18 to 1.81)	NA	NA
Relaxation	1.80 (−7.29 to 10.91)	NA	NA
Electrotherapy	−9.16 (−21.59 to 2.98)	−0.78 (−5.26 to 3.62)	−0.08 (−4.13 to 3.97)
LD flexibility exercise	5.07 (−12.93 to 23.41)	NA	NA
PT/BT sleep	−11.68 (−20.34 to −3.11)	NA	NA
AQ mind‐body exercise	2.02 (−6.29 to 10.45)	NA	NA
AQ Mixed exercise	1.51 (−7.50 to 10.66)	NA	NA
Weight loss	−3.75 (−13.74 to 6.21)	NA	NA
Neuromodulation	0.37 (−8.82 to 9.53)	NA	NA
Nonmainstream practice	−6.20 (−15.49 to 3.18)	3.84 (−1.68 to 9.22)	1.73 (−2.45 to 5.75)
HBOT	−26.29 (−37.56 to −15.15)	NA	NA
LD aerobic exercise + LD flexibility exercise	−19.91 (−34.89 to −4.94)	NA	NA
Generic PT/BT + relaxation	−12.07 (−20.75 to −3.35)	NA	NA
Multidisciplinary	−17.31 (−28.38 to −6.29)	NA	NA
LD flexibility exercise + manual therapy	−0.32 (−26.12 to 25.78)	NA	NA
Balneotherapy + AQ mixed exercise	−5.75 (−18.97 to 7.71)	NA	NA
Tricyclics	−10.63 (−27.49 to 6.09)	NA	NA
Antipsychotics	−6.63 (−19.43 to 6.12)	NA	NA
Antioxidant	−17.75 (−34.91 to −0.61)	7.27 (−1.68 to 15.62)	5.00 (−0.43 to 10.80)
SRI	−9.85 (−15.80 to −3.80)	1.79 (−0.84 to 4.62)	1.50 (−0.45 to 3.79)
Iron replacement	−15.10 (−30.41 to −0.06)	NA	NA
Gabapentinoid	−3.86 (−8.18 to 0.40)	0.87 (−2.22 to 4.11)	0.10 (−2.32 to 2.51)
Analgesic	−3.29 (−11.68 to 5.19)	NA	NA
CNS depressants	−8.83 (−14.77 to −2.74)	1.09 (−1.80 to 4.22)	2.93 (1.10–4.79)

* AQ, aquatic; CNS, central nervous system; Crl, credible interval; FIQ, Fibromyalgia Impact Questionnaire; HBOT, hyperbaric oxygen therapy; LD, land‐based; MCS, mental component summary; MD, mean difference; NA, not applicable; PCS, physical component summary; PT/BT, psychological or behavioral therapy; PT/BT sleep, PT/BT targeted to sleep; SF‐36, Short Form 36 Health Survey; SRI, serotonin reuptake inhibitor.

^a^ Higher scores indicate a worse outcome.

^b^ Negative values indicate a worse outcome, whereas positive values indicate a better outcome.

### QoL outcome: SF‐36 MCS score

Of the studies that reported SF‐36 MCS, 15 of 17 (n = 359, 13 interventions) were included in the NMA (two were excluded as they were not linked in the network). Land‐based mind‐body exercise (n = 281; MD 7.27, 95% Crl 1.11–13.94) and education (n = 22; MD 10.31, 95% Crl 2.06–19.35) were associated with an improvement in SF‐36 MCS score compared with placebo or sham (Table 2, Supplementary Material 4). In contrast, there was evidence that SF‐36 MCS scores were worse after nutrition (n = 36) than after placebo or sham (n = 1,167; MD −7.96, 95% Crl −14.83 to −1.11), but there was no clear evidence that SF‐36 MCS scores were worse after usual care and electrotherapy than after placebo or sham. The remaining interventions showed no clear evidence of a positive effect when compared with placebo or sham.

### QoL outcome: SF‐36 PCS score

Of the studies that reported SF‐36 PCS scores, 16 of 17 (n = 401 participants, 13 interventions) were included in the analysis (one was excluded as it did not link with other studies). Compared with placebo or sham (n = 1,355), a better SF‐36 PCS score was recorded after land‐based mind‐body exercise training (n = 281; MD 7.61, 95% Crl 3.56–13.06), land‐based aerobic exercise training (n = 75; MD 6.17, 95% Crl 1.05–12.81), and use of CNS depressants (n = 874; MD 2.93, Crl 1.10–4.79) (Table 2, Supplementary Material 4). There was insufficient evidence that electrotherapy (n = 20) had a positive effect on the SF‐36 PCS score compared with placebo or sham (MD −0.82, 95% Crl −4.13 to 3.97), and there was no clear evidence that the effects of the remaining interventions were different from those of placebo or sham.

### Sleep duration

Sleep duration was reported in two studies (n = 363 participants, three interventions). There was insufficient evidence that gabapentinoid (n = 169) increased sleep duration compared with placebo or sham (n = 179; MD 7.40, 95% Crl −9.84 to 24.74), whereas SRI (n = 15) appeared to be detrimental to sleep duration compared with placebo or sham (n = 179; MD −24.40, 95% Crl −59.81 to 21.96) (see Supplementary Material S5).

### Consistency between direct and indirect evidence

For sleep quality, there was evidence of inconsistency between direct and indirect evidence for usual care and aquatic‐based aerobic exercise compared with land‐based mind‐body exercise, land‐based flexibility exercise compared with usual care, and land‐based flexibility exercise compared with aquatic‐based aerobic exercise (Supplementary Material S4). For FIQ, for some intervention comparisons (generic psychological or behavioral therapy compared with placebo or sham, and sleep‐focused psychological or behavioral therapy compared with education or usual care), the node‐splitting analysis showed significant disagreement (inconsistency) between direct and indirect estimates (Supplementary Material S5). For SF‐36 MCS and PCS, there was no need to check for the presence of inconsistency between direct and indirect estimates as the only two closed loops in the network were from a single three‐arm trial (Supplementary Material S5).

### Ranking of interventions

For sleep quality and FIQ, hyperbaric oxygen therapy and land‐based aerobic with flexibility exercise training were ranked as the top two interventions (these were not evaluated for SF‐36). However, it is important to note that SUCRA does not consider the magnitude of differences in effects between interventions, as well as the body and quality of evidence that contributes to each treatment comparison. Moreover, between the five considered outcomes, we observed some inconsistencies. For example, antioxidant therapy and land‐based mind‐body exercise were ranked low for the sleep quality outcome but not for FIQ. For these reasons, we have little confidence in the SUCRA findings alone.

### Adverse events

Data on adverse events were available from 18 of 90 studies (20%) that assessed pharmacologic interventions and 2 of 90 studies (2.2%) that assessed nonpharmacologic interventions. Due to the heterogeneity across included studies, we have summarized adverse events narratively.

In general, nonpharmacologic treatments under investigation were generally well tolerated, with most reported adverse events being mild or moderate in severity such as stiffness and fatigue. In contrast, pharmacologic treatments were commonly associated with adverse events like dizziness, somnolence, headache, and dry mouth.

## DISCUSSION

This evidence synthesis included 90 RCTs assessing sleep quality in patients with fibromyalgia. To our knowledge, our study is the most comprehensive approach to assess the current evidence on pharmacologic and nonpharmacologic interventions for fibromyalgia‐related sleep problems.

The findings of our NMA on sleep quality using validated PROMs show that, compared with placebo or sham treatment, some forms of exercise such as land‐based aerobic exercise training combined with flexibility exercise training and aquatic aerobic exercise training, may improve sleep quality, although our certainty in the current evidence is generally low. For all other pharmacologic and nonpharmacologic interventions, there was a modest effect on sleep quality compared with placebo or sham treatment (Crl indicated uncertainty, and the certainty of evidence is low to very low). Notably, we did not observe a significant, beneficial effect of pharmacologic interventions on sleep quality.

Compared with placebo or sham treatment, some interventions positively affected participants’ QoL. Using the FIQ, an improvement in QoL was observed among participants who undertook land‐based aerobic and flexibility exercise training, multidisciplinary training, land‐based mind‐body exercise training, either generic PT/BT or PT/BT sleep, generic PT/BT alongside relaxation, and pharmacologic treatments including antioxidant, iron replacement, SRIs, and CNS depressants, although the magnitude of the effect varied. An improvement in the SF‐36 MCS score was observed after land‐based mind‐body exercise and education interventions, whereas an improvement in the SF‐36 PCS score was observed after land‐based mind‐body exercise training, land‐based aerobic exercise training, and use of CNS depressants.

Overall, our analyses were hampered by the lack of head‐to‐head comparisons for active treatments. Most interventions were compared with either placebo, sham, or usual care. Some of the nonpharmacologic studies failed to compare their active interventions with sham procedures that involved appropriate control strategies in terms of exposure time (frequency and duration) and “attention” received from the therapist and/or instructor. Appropriate sham controls have been used in similar clinical areas and are considered particularly useful for studies with subjective or self‐reported endpoints (eg, improvement of symptoms) and when the risk of the sham procedures is low (eg, less intensive or generic physical activity or procedure).^50^, ^51^ Furthermore, the recent Control Interventions in Physical, Psychological, and Self‐Management Therapy Trials (CoPPS) statement recommends designing control interventions that are as similar as possible to the interventions under investigation, apart from the components the trial aims to assess.^52^ Appropriate sham‐control could potentially reduce bias by allowing for blinding of participants.^53^ Blinding in nonpharmacologic RCTs can be challenging. When patients are unblinded and aware they are receiving the treatment under investigation, their self‐reported outcomes may be biased by higher expectations of improvement. Conversely, those who know they are not receiving the intervention may have much lower expectations or even experience a nocebo response.^54^, ^55^

Most of the studies that contributed to the network were small (<100 participants), with short‐term follow up (around 3 months) and assessed a diverse range of interventions. To make the NMA feasible, we grouped the 97 different active interventions into 45 categories according to their characteristics and mode of action; however, inevitably, the individual interventions varied within the category groups. The limited number of studies available for each intervention comparison also precluded a meaningful assessment of publication bias. We were also only able to analyze average treatment effects and not relevant clinical and demographic modifiers at the patient level (eg, severity of disease, duration of illness, extent and nature of sleep disturbances, and level of physical activity before and during treatment). Sleeplessness may also be exacerbated by mood disorders such as depression, which are common among people with fibromyalgia; however, due to inconsistent reporting across studies, we could not explore this further.^56^

Unfortunately, our primary outcome, sleep quality, was not consistently and objectively measured across studies; several different PROMs were used. Because there is no consensus on which is the best outcome measure to use in the field of fibromyalgia, we decided to combine studies irrespective of the way sleep quality was measured, provided that a validated instrument was used. This might have contributed to heterogeneity and inconsistency in the network, thus limiting the reliability of our findings. Furthermore, the interpretation of results was complicated by the lack of information on their minimally important clinical difference. Our original plan was to conduct a component NMA to disentangle the effect of each component of the interventions assessed by the included studies, but this proved impossible due to the lack of suitable data.

According to CINeMA, for many comparisons included in our NMA, our certainty of the evidence was rated as low to very low (sleep quality outcome). The level of certainty was downgraded for within‐study bias, primarily due to an inadequate reporting of randomization and allocation concealment methods as well as issues related to missing outcome data. The certainty level was also downgraded for imprecision because of the low number of studies available for each comparison and their small sample size, as well as heterogeneity and incoherence across comparisons. Given our CINeMA findings, we were unable to conduct sensitivity analyses restricted to high‐quality studies.

There are several published systematic reviews assessing different forms of exercise training and other nonpharmacologic interventions for the management of fibromyalgia symptoms.^57^, ^58^, ^59^, ^60^ Although not primarily focused on sleep problems, they all identify similar limitations to those we observed here, including heterogeneity across studies in terms of study protocols, insufficient evidence to establish the effectiveness of one intervention compared with another, lack of appropriate comparator treatments, insufficient statistical power in most studies and low‐to‐moderate quality of the evidence. One systematic review that aimed to evaluate the effectiveness of nonpharmacologic treatments for fibromyalgia revealed that all types of exercise, except for flexibility exercises, helped reduce pain intensity, whereas aerobic and strengthening exercises helped improve sleep quality.^61^ However, the authors identified only a limited number of studies, usually of small sample sizes, for each form of exercise (10 for aerobic exercise, 9 for strengthening, and 2 for flexibility) and found considerable heterogeneity in outcome measures, intervention programs, and control interventions, in line with our findings.

Overall, there is a suggestion that some forms of exercise training, psychological and behavioral therapy, and some pharmacologic treatments may play a role in improving fibromyalgia‐related sleep problems and/or patients’ QoL. However, the limitations of the current evidence do not allow reliable conclusions about optimal interventions for treating sleep problems in people with fibromyalgia. There is a clear need to improve the quality of existing evidence. It is worth noting that most participants were middle‐aged women from high‐income countries. Information on ethnicity and level of education was often not reported. Future studies should be properly designed and include an adequate number of diverse patients to reduce bias and to ensure results are generalizable.^62^ Interventions should be compared with established therapies or adequate sham treatments to demonstrate their comparative efficacy and safety. Descriptions of placebo and sham treatments should be guided by the Template for Intervention Description and Replication‐Placebo checklist.^63^ There should also be consensus on the best way to capture fibromyalgia symptoms. Currently, having different tools to measure symptoms not only impacts the ability to synthesize research evidence but also confuses health professionals and patients when trying to document and tackle fibromyalgia‐related symptoms. The development of a core outcome set for measuring sleep outcomes in both adults and children with fibromyalgia would be beneficial for informing new standardized PROMs in this field. It would also be crucial to involve people with fibromyalgia in the conception and content validation of any tool measuring sleep, ensuring that PROMs cover what matters most to patients.

## AUTHOR CONTRIBUTIONS

All authors contributed to at least one of the following manuscript preparation roles: conceptualization AND/OR methodology, software, investigation, formal analysis, data curation, visualization, and validation AND drafting or reviewing/editing the final draft. As corresponding author, Dr Brazzelli confirms that all authors have provided the final approval of the version to be published, and takes responsibility for the affirmations regarding article submission (eg, not under consideration by another journal), the integrity of the data presented, and the statements regarding compliance with institutional review board/Declaration of Helsinki requirements.

## Supporting information


**Disclosure form**.

**Appendix 1:** Literature searches

**Appendix 2:** Characteristics of studies reporting patient‐reported outcome measures (PROMs) of sleep quality included in the network meta‐analysis (ordered by intervention category comparison)

**Appendix 3:** Network‐meta‐analysis results for sleep quality outcome

**Appendix 4:** Network‐meta‐analysis results for other outcomes
