# Supplementary material for: Effects of Pharmacologic and Nonpharmacologic Interventions for the Management of Sleep Problems in People With Fibromyalgia: Systematic Review and Network Meta‐Analysis of Randomized Controlled Trials
Source: Arthritis Care Res (Hoboken). 2025 Mar 26;77(9):1095–105. doi: 10.1002/acr.25505 (PMC12371313; doi:10.1002/acr.25505)
Supplement: Supplementary file 2 — Appendix 1: Literature searches [file ACR-77-1095-s002.docx]

**Appendix 1 Literature searches**

Ovid **MEDLINE**(R) and Epub Ahead of Print, In-Process, In-Data-Review & Other Non-Indexed Citations, Daily and Versions(R) <1946 to October 29, 2021>

1. Fibromyalgia/

2. (fibromyalg$ or fibrosit$ or FMS or muscular rheumatism).tw,kf.

3. (chronic adj2 widespread adj2 pain).tw,kf.

4. (chronic adj2 diffuse adj2 pain).tw,kf.

5. or/1-4

6. sleep/ or Sleep Wake Disorders/ or sleep deprivation/ or sleep hygiene/ or "Sleep Initiation and Maintenance Disorders"/

7. (sleep* or wakefulness or waking or awake$ or sleeplessness or insomni$).tw,kf.

8. 6 or 7

9. randomized controlled trial.pt.

10. controlled clinical trial.pt.

11. randomized.ab.

12. placebo.ab.

13. drug therapy.fs.

14. randomly.ab.

15. trial.ab.

16. groups.ab.

17. or/9-16

18. exp animals/ not humans/

19. 17 not 18

20. 5 and 8 and 19

**Embase** <1974 to 2021 Week 43>

1. fibromyalgia/

2. (fibromyalgia or fibrositis).tw,kf.

3. (chronic adj2 widespread adj2 pain).tw,kf.

4. (chronic adj2 diffuse adj2 pain).tw,kf.

5. 1 or 2 or 3 or 4

6. sleep/ or sleep deprivation/ or sleep hygiene/ or exp sleep disorder/

7. (sleep* or wakefulness or waking or awake$ or sleeplessness or insomni$).tw,kf.

8. 6 or 7

9. Randomized controlled trial/

10. Controlled clinical study/

11. randomization/

12. double blind procedure/

13. random$.tw,kf.

14. placebo.ti,kf.

15. ((doubl* or singl*) adj blind).tw,kf.

16. (assigned or allocated).tw,kf.

17. (controlled adj7 (study or design or trial)).tw,kf.

18. 9 or 10 or 11 or 12 or 13 or 14 or 15 or 16 or 17

19. 5 and 8 and 18

APA **PsycInfo** <1967 to October Week 4 2021>

1 fibromyalgia/ 2162

2 (fibromyalgia or fibrositis or FMS or muscular rheumatism).tw. 4069

3 (chronic adj2 widespread adj2 pain).tw. 300

4 (chronic adj2 diffuse adj2 pain).tw. 18

5 1 or 2 or 3 or 4 4209

6 Sleep/ or sleep wake disorders/ or sleep deprivation/ or sleepiness/ 36375

7 (sleep* or wakefulness or waking or awake$ or sleeplessness or insomni$).tw. 92877

8 6 or 7 93325

9 Randomized Controlled Trial/ 783

10 randomized controlled trials/ or randomized clinical trials/ 1041

11 treatment effectiveness evaluation/ 26005

12 random$.tw. 216081

13 placebo.tw. 41537

14 ((doubl* or singl*) adj blind).tw. 25676

15 (assigned or allocated).tw. 85585

16 (controlled adj7 (study or design or trial)).tw. 52172

17 9 or 10 or 11 or 12 or 13 or 14 or 15 or 16 300618

18 5 and 8 and 17 114

**AMED** (Allied and Complementary Medicine) <1985 to October 2021>

1. fibromyalgia/

2. (fibromyalg$ or fibrosit$ or FMS or muscular rheumatism).tw,hw.

3. (chronic adj2 widespread adj2 pain).tw,hw.

4. (chronic adj2 diffuse adj2 pain).tw,hw.

5. or/1-4

6. sleep/ or Sleep disorders/

7. (sleep* or wakefulness or waking or awake$ or sleeplessness or insomni$).tw,hw.

8. 6 or 7

9. randomized controlled trial.pt.

10. controlled clinical trial.pt.

11. randomized.ab.

12. placebo.ab.

13. randomly.ab.

14. trial.ab.

15. groups.ab.

16. or/9-15

17. 5 and 8 and 16

**Web of Science** Science Citation Index Expanded (SCI-EXPANDED), Conference Proceedings Citation Index – Science (CPCI-S)

1 fibromyalg* or fibrosit* or FMS or "muscular rheumatism" (Topic) 28,032

2 chronic NEAR/2 widespread NEAR/2 pain (Topic) 1,547

3 chronic NEAR/2 diffuse NEAR/2 pain (Topic) 61

4 #1 or #2 or #3 28,534

5 sleep* or wakefulness or waking or awake* or sleeplessness or insomni* (Topic) 340,778

6 (random* OR clinical) NEAR/3 (study OR trial) (Topic) 1,212,201

7 RCT or "double blind" or "single blind" or random* or trial (Topic) 2,887,547

8 #6 or #7 3,091,797

9 #4 and #5 and #8 869

**CINAHL**

S1 (MH "Fibromyalgia")

S2 TX fibromyalg$ OR fibrosit$ OR FMS OR muscular rheumatism

S3 TX chronic N2 widespread N2 pain

S4 chronic N2 diffuse N2 pain

S5 s1 OR s2 OR s3 OR s4

S6 (MH "Sleep") OR (MH "Sleep Disorders") OR (MH "Sleep Deprivation") OR (MH "Sleep Hygiene")

S7 sleep* OR wakefulness OR waking OR awake$ OR sleeplessness OR insomni$

S8 S6 OR S7

S9 MH randomized controlled trials

S10 MH double-blind studies

S11 MH single-blind studies

S12 MH random assignment

S13 TI (randomised OR randomized)

S14 AB (random*)

S15 TI (trial)

S16 MH (placebos)

S17 PT (randomized controlled trial)

S18 AB (control W5 group)

S19 S9 OR S10 OR S11 OR S12 OR S13 OR S14 OR S15 OR S16 OR S17 OR S18

S20 S5 AND S8 AND S19

**CENTRAL**

#1 MeSH descriptor: [Fibromyalgia] this term only 1500

#2 fibromyalg* or fibrosit* or FMS or "muscular rheumatism" 3979

#3 chronic NEAR/2 widespread NEAR/2 pain 213

#4 chronic NEAR/2 diffuse NEAR/2 pain 11

#5 #1 or #2 or #3 or #4 4024

#6 MeSH descriptor: [Sleep] this term only 4243

#7 MeSH descriptor: [Sleep Wake Disorders] this term only 1819

#8 MeSH descriptor: [Sleep Deprivation] this term only 793

#9 MeSH descriptor: [Sleep Initiation and Maintenance Disorders] this term only 2644

#10 sleep* or wakefulness or waking or awake* or sleeplessness or insomni* 57593

#11 #6 or #7 or #8 or #9 or #10 57593

#12 #5 and #11 986 [849 trials]
