# Supplementary material for: Effects of Pharmacologic and Nonpharmacologic Interventions for the Management of Sleep Problems in People With Fibromyalgia: Systematic Review and Network Meta‐Analysis of Randomized Controlled Trials
Source: Arthritis Care Res (Hoboken). 2025 Mar 26;77(9):1095–105. doi: 10.1002/acr.25505 (PMC12371313; doi:10.1002/acr.25505)
Supplement: Supplementary file 3 — Appendix 2: Characteristics of studies reporting patient‐reported outcome measures (PROMs) of sleep quality included in the network meta‐analysis (ordered by intervention category comparison) [file ACR-77-1095-s003.docx]

## Appendix 2 Characteristics of studies reporting patient-reported outcome measures (PROMs) of sleep quality included in the network meta-analysis (ordered by intervention category comparison)

| **Intervention category comparison** | **Study ID (Author, Year, Reference ID)** | **Duration of treatment (Tx) or first assessment (Ax), if later (weeks)** | **Intervention** | **Number randomised and gender (female [F], male [M], %)** | **Age, years, mean (SD)** | **BMI kg/m^2^, mean (SD)** | **Quality of life measures, mean (SD)** | **Ethnicity, n (%)** | **Education status, n (%)** |
| --- | --- | --- | --- | --- | --- | --- | --- | --- | --- |
| NON-PHARMACOLOGICAL INTERVENTIONS |  |  |  |  |  |  |  |  |  |
| Studies reporting Pittsburgh Sleep Quality Index (PSQI) outcome |  |  |  |  |  |  |  |  |  |
| UC vs. Balneotherapy | Maindet 2021^1^  Ref ID 704 | 24  (Tx 3w; Ax at 24w) | Usual care | 108  F92% M8% | 49.2 (8.8) | 27.7 (5.8) | NR | NR | < Baccalaureate: 31.5%; High school diploma (baccalaureate):30.6%; University degree or higher: 38.0% |
|  |  |  | Spa therapy | 110  F90% M10% | 50.4 (8.9) | 26.6 (6.6) | NR | NR | < Baccalaureate: 31.8%; High school diploma (baccalaureate):25.5%; University degree or higher: 42.7% |
| UC vs. Flex/skill LD | Ceca 2020^2^  Ref ID 2297 | 20 | Waitlist | 33  F95% M5% | 57.4 (4.5) | NR | NR | White: 100% | NR |
|  |  |  | Self-myofascial conditioning programme | 33  F87% M13% | 50.6 (7.1) | NR | NR | White: 100% | NR |
| UC vs. Manual T | Castro-Sánchez 2014^3^  Ref ID 237 | 5 | No treatment | 44  F55% M45% | 53 (7) | NR | 67.6 (12.1) | NR | NR |
|  |  |  | Manual therapy | 45  F53% M47% | 54 (8) | NR | 66.7 (13.4) | NR | NR |
| UC  vs. Mind-body Ex LD | Jiao 2019^4^  Ref ID 215 | 12 | Waitlist (stable usual therapy) | 31  F87% M13% | 53.5 (0.6) | 26.3 (3.3) | FIQ 46.1 (16.8)  SF36 PCS 31.2 (15.2)  SF36 MCS 50.8 (19.2) | Chinese: 100% | <9 grade: 10%; High school: 23%; College: 61%; Postgraduate: 6% |
|  |  |  | Ba-Duan-Jin | 31  F84% M16% | 48.9 (10.2) | 23.4 (2.4) | FIQ 46.6 (15.4)  SF36 PCS 35.2 (18.5)  SF36 MCS 52.8 (20) | Chinese: 100% | <9 grade: 10%; High school: 26%; College: 55%; Postgraduate: 10% |
| UC vs. Mind-body Ex LD | Lynch 2012^5^  Ref ID 327 | 8 | Control (waitlist/ usual care) | 47  F98% M2% | 52.1 (8.6) | NR | FIQ 61.83 (13.36)  SF36 PCS 32.55 (8.82)  SF36 MCS 40.38 (10.08) | NR | NR |
|  |  |  | Qigong | 53  F94% M6% | 52.8 (8.9) | NR | FIQ 65.53 (14.44)  SF36 PCS 29.94 (8.3)  SF36 MCS 38.13 (9.62) | NR | NR |
| UC vs. Mx Exercise AQ | Munguía-Izquierdo 2008^6^  Ref ID 199 | 16 | Control | 25  F100% | 46 (8) | 27 (4) | FIQ 63.6 (16.7) | White: 100% | Highest education (%) Elementary school 63; High school 29; College/university 8; |
|  |  |  | Aquatic exercise | 35  F100% | 50 (7) | 27 (4) | FIQ 68.1 (12.4) | White: 100% | Highest education (%) Elementary school 56; High school 32; College/university 12 |
| UC vs. PBO/Sham vs. Non-MSM practice | Lauche 2016^7^  Ref ID 217 | 18 days | Usual care | 46  F100% | 56.8 (7.7) | 28.2 (5.4) | FIQ 61.4 (14.1)  SF36 PCS 31.2 (7.6)  SF36 MCS 40.1 (13) | NR | <High school: 69.6%; High school: 13.0%; University degree: 17.4% |
|  |  |  | Sham cupping | 48  F98% M2% | 56.3 (8.7) | 27.2 (4.7) | FIQ 61.8 (11.8)  SF36 PCS 31.7 (7.4)  SF36 MCS 37.6 (11.1) | NR | <High school: 33 (68.8); High school: 8 (16.7); University degree: 7 (14.6) |
|  |  |  | Cupping therapy | 47  F98% M2% | 54.35 (10.6) | 29.4 (7.3) | FIQ 55.7 (12.8)  55.7 (12.8)  SF36 PCS 31.6 (7.3)  SF36 MCS 41.1 (12.1) | NR | <High school: 35 (74.5); High school: 10 (21.3); University degree: 2 (4.3) |
| UC vs. Nutrition | San Mauro Martin 2019^8^  Ref ID 651 | 4 | No supplement | 7  F100% | 51.7 (7.5) | 27.0 (6.7) | NR | NR | NR |
|  |  |  | Turmeric-based food supplement | 6  F100% | 51.2 (9.4) | 28.1 (4.5) | NR | NR | NR |
| UC vs. PBO/Sham vs. Nutrition | Barmaki 2019^9^  Ref ID 268 | 24 | No supplementary treatment | 31  F100% | 47.8 (9.0) | 25.58 (5.25) | SF12 PCS 29 (5.7)  SF12 MCS 39 (9.1) | NR | NR |
|  |  |  | Food supplement (as placebo) | 33  F100% | 47.4 (8.6) | 25.54 (5.14) | SF12 PCS 27.5 (6.3)  SF12 MCS 35.8 (11) | NR | NR |
|  |  |  | Phytotherapy treatment (Fib-19-01) | 36  F100% | 49.6 (9.4) | 27.23 (6.02) | SF12 PCS 26.5 (7.1)  SF12 MCS 36.8 (10) | NR | NR |
| UC vs. PT/BT gen | Amutio 2018^10^  Ref ID 1397 | 7 | Waitlist | 19  F100% | 51.8 (10.2) whole pop | NR | NR | NR | No formal education: 8%; Primary-school education: 62%; Intermediate studies education: 16%; 14% had a higher education  (whole pop) |
|  |  |  | Mindfulness treatment | 20  F100% |  | NR | NR | NR |  |
| UC vs. PT/BT gen | Simister 2018^11^  Ref ID 265 | 8 | TAU | 34 | 39.7 (9.36) whole pop | NR | FIQR 55.28 (16.39) | NR | 87% of the sample had at least a high school education and 59% had education beyond the high school level (whole pop) |
|  |  |  | Online ACT + TAU | 33  (F95% M5% whole pop) |  | NR | FIQR 55.83 (12.56) | NR |  |
| UC vs. PBO/Sham vs. PT/BT gen | Schmidt 2011^12^  Ref ID 349 | 8 | Wait list | 59  F100% | 52.3 (10.9) | NR | FIQ 5.65 (1.86) | NR | No school completed: 1.7%; 9 years: 30.5%; 11 years/GCSE: 25.4%; A-level/college entry: 42.4%; Missing data: 0 |
|  |  |  | Active control (muscle relaxation and stretching) | 59  F100% | 51.9 (9.2) | NR | FIQ 5.5 (1.68) | NR | No school completed: 0; 9 years: 28.6%; 11 years/GCSE: 39.3%; A-level/college entry: 30.4%; Missing data: 1.8% |
|  |  |  | Mindfulness-based stress reduction | 59  F100% | 53.4 (8.7) | NR | FIQ 5.84 (1.37) | NR | No school completed: 1.9%; 9 years: 34.0%; 11 years/GCSE: 41.5%; A-level/college entry: 20.8%; Missing data: 1.9% |
| UC vs. PT/BT gen vs. PT/BT sleep | Lami 2018^13^  Ref ID 458 | 10 (Tx 9w; Ax at 10w) | Usual medical care (UMC) | 42  F100% | 51.4 (9.4) | NR | FIQ 55.57 (18.14) | NR | Basic education: 26.8%; High school: 34.2%; Professional instruction: 17.1%; University studies: 22% |
|  |  |  | CBT-P (CBT for pain) | 42 F100% | 49.4 (6.4) | NR | FIQ 65.53 (11.08) | NR | Basic education: 29.4%; High school: 44.1%; Professional instruction: 20.6%; University studies: 5.9% |
|  |  |  | CBT-IP (CBT for insomnia and pain) | 42  F100% | 49.7 (8.4) | NR | FIQ 61.98 (11.14) | NR | Basic education: 31.5%; High school: 21.1%; Professional instruction: 7.9%; University studies: 39.5% |
| UC vs. Relaxation/Medication | Onieva-Zafra 2019^14^  Ref ID 689 | Unclear (8w?) | Control | 27? F96% M4% | 51.3 (6.5) | NR | NR | NR | NR |
|  |  |  | Guided imagery | 29?  F97% M3%  (study enrolled 60 in total) | 53.6 (5.8) | NR | NR | NR | NR |
| UC vs. Weight loss | Senna 2012^15^  Ref ID 345 | 24 | No weight loss | 43  F90% M10% | 46.3 (14.4) | 32.8 (1.4) | FIQ 53.2 (11.55) | NR | Education >high school: 59.5% |
|  |  |  | Dietary weight loss | 43  F90% M10% | 44.8 (13.6) | 32.3 (1.4) | FIQ 54.6 (13.1) | NR | Education >high school: 58.5% |
| PBO/Sham vs. Aerobic LD + Relaxation/Meditation | Arcos-Carmona 2011^16^  Ref ID 358 | 10 | Sham magnet therapy | 28  F100% M0% | 44.4 (9.25) whole pop | NR | NR | NR | NR |
|  |  |  | Aerobic exercise + progressive relaxation technique | 28  F100% M0% |  | NR | NR | NR | NR |
| PBO/Sham vs. Manual T | Castro-Sanchez 2011^17^  Ref ID 711 | 20 | (Sham) magnotherapy | 32  F96% M4% | 46.3 (12.3) | NR | NR | NR | No school: 65.5%; Primary school: 24.1%; Secondary school: 3.4%; University: 6.9% |
|  |  |  | Massage-myofascial release therapy | 32  F94% M6% | 49.3 (11.6) | NR | NR | NR | No school: 73.3%; Primary school: 13.3%; Secondary school: 10%; University: 3.3% |
| PBO/Sham  vs. Manual T | Nadal-Nicolás 2020^18^  Ref ID 2308 | 4 | Placebo (sham ultrasound) | 15  F100% | 53 (6) whole pop | 28.7 (4.1) | NR | NR | NR |
|  |  |  | Manual therapy | 15  F100% | -- | 28.4 (4.3) | NR | NR | NR |
| PBO/Sham  vs. Mind-body Ex AQ | Ide 2008^19^  Ref ID 493 | 4 | Control (supervised recreational activities) | 20  F100% | 45.5 (8.7) | NR | FIQ 6.22 (1.71)  SF36 PCS 44.12 (24.38)  SF36 MCS 39.12 (25.89) | White: 95% | NR |
|  |  |  | Aquatic respiratory exercise-based program | 20  F100% | 46.6 (9.8) | NR | FIQ 6.64 (1.51)  SF36 PCS 40.28 (19.74)  SF36 MCS 41.11 (22.03) | White: 95% | NR |
| PBO/Sham  vs. Mind-body Ex LD | Liu 2012^20^  Ref ID 110 | 6 | Sham (specially developed sham Quigong exercise) | 6  Gender NR | 57.5 (range 45-70) | NR | NR | NR | NR |
|  |  |  | Qigong | 8 Gender NR | 55.7 (range 20-70) | NR | NR | NR | NR |
| PBO/Sham  vs. Mind-body Ex LD | Sarmento 2020^21^  Ref ID 409 | 10 | Sham Qigong | 14  F100% M0% | 56.1 (12.3) | NR | FIQR 57.6 (16.7) | NR | NR |
|  |  |  | Qigong | 14  F100% M0% | 42.6 (10.7) | NR | FIQR 52.1 (18.5) | NR | NR |
| PBO/Sham + Multicompoment therapy (MT)   vs. Neuromodulation + MT (Aerobic LD + Flex/skill AQ + Relaxation/Medication + Education) | Guinot 2021^22^  Ref ID 2313 | 12 | Sham rTMS + Multi-component therapy (MT) | 19  F79% M21% | 42.8 (8.8) | 25.1 (4.5) | FIQ 62.9 (9.9) | NR | College: 63.2%; High school: 36.8% |
|  |  |  | rTMS + MT (aerobic training [land-based] + pool-based therapy [balance and posture work] + relaxation + education) | 20  F100% | 46.5 (10.4) | 26.7 (4.8) | FIQ 64.3 (12) | NR | College: 50.0%; High school: 50.0% |
| PBO/Sham + Multimodal   vs. Manual T + Multimodal (PT/BT gen + Flex/skill LD) | Moustafa 2015^23^  Ref ID 346 | 12 | Control (multimodal programme, plus manual contact similar to manipulative therapy) | 60  F45% M55% | 51.4 (7) | NR | FIQ 71.3 (5.8) | NR | Primary school: 23.5 %; Secondary school: 28.5%; Advanced technical college: 21.5 %; University diploma: 21.5 %; Other: 5 % |
|  |  |  | Upper cervical manipulative therapy + multimodal program (consisting of CBT and stretching exercise) | 60  F42% M58% | 53.5 (8) | NR | FIQ 70.9 (4.4) | NR | Primary school: 20 %; Secondary school: 32 %; Advanced technical college: 25 %; University diploma: 18 %; Other: 5 % |
| PBO/Sham vs. Neuromodulation | Goldway 2019^24^  Ref ID 227 | 5 | Sham neurofeedback | 12  F78% M22% | 35.9 (10.6) | NR | NR | NR | NR |
|  |  |  | Amygdala electrical-finger-print (Amyg-EFP) neurofeedback | 31  F96% M4% | 35.5 (12.6) | NR | NR | NR | NR |
| PBO/Sham vs. Neuromodulation | Samartin-Veiga 2021^25^  Ref ID 1787 | Unclear (Tx 3w, Ax at 4w?) | Sham tDCS | 30  F100% M0% | 50.67 (8.88) | NR | NR | NR | NR |
|  |  |  | M1-tDCS (classic) | 34  F100% M0% | 49.38 (8.83) | NR | NR | NR | NR |
|  |  |  | DLPFC-tDCS (classic) | 33  F100% M0% | 50.55 (8.89) | NR | NR | NR | NR |
|  |  |  | OIC-tDCS (novel) | 33  F100% M0% | 50.21 (8.20) | NR | NR | NR | NR |
| PBO/Sham vs. Neuromodulation | Wu 2021^26^  Ref ID 2311 | 8 | Telephone support (control) | 20  F70% M30% | 42.2 (10.9) | 23.8 (5.1) | FIQR 47.3 (23.12) | NR | ≤High school: 30.0%; College: 55.0%; ≥Graduate school: 15.0% |
|  |  |  | Neurofeedback | 60  F95% M5% | 48.6 (13.5) | 21.9 (3.9) | FIQR 55.08 (17.69) | NR | ≤High school: 26.7%; College: 65.0%; ≥Graduate school: 8.3% |
| PBO/Sham + SSRI vs. Nutrition + SSRI | Mirzaei 2018^27^  Ref ID 1967 | 8 | Placebo + Trazodone | 37  Gender NR | 41 (10.3) | NR | FIQ 50.7 (16)  SF36 PCS 49.6 (9.3)  SF36 MCS 50.19 (9.1) | NR | NR |
|  |  |  | Vitamin D + Trazodone | 37  Gender NR | 42.1 (10.8) | NR | FIQ 53.4 (16.6)  SF36 PCS 50.3 (11.24)  SF36 MCS 49.8 (11.3) | NR | NR |
| PBO/Sham vs. Non-MSM practice | Mataran-Penarrocha 2011^28^  Ref ID 659 | 25 | Placebo (sham ultrasound treatment) | 52  F98% M2% | 52.3 (11.0) | NR | NR | NR | NR |
|  |  |  | Craniosacral therapy | 52  F95% M5% | 48.2 (13.3) | NR | NR | NR | NR |
| Aerobic LD vs. Aerobic LD + Flex/skill LD | Gómez-Hernández 2020^29^  Ref ID 603 | 12 | Control (stationary cycling) | 32  F100% M0% | 54.6 (8.5) | 20.5 (1.7) | FIQ 83.65 (3.36) | NR | NR |
|  |  |  | Flexibility | 32  F100% M0% | 54.0 (5.0) | 21.0 (1.9) | FIQ 84.1 (4.12) | NR | NR |
| Aerobic AQ vs. Mind-body Ex LD | de Medeiros 2020^30^  Ref ID 209 | 12 | Aquatic aerobic exercise | 21  F100% | 50.7 (9.7) | 30.4 (5.2) | FIQ 67 (16) | NR | NR |
|  |  |  | Mat pilates | 24  F100% | 45.5 (10.6) | 27.8 (4.7) | FIQ 68 (14) | NR | NR |
| Aerobic LD  vs. Mind-body Ex LD vs. Mind-body Ex LD vs. Mind-body Ex LD vs. Mind-body Ex LD | Wang 2018^31^  Ref ID 276 | 24 | Aerobic exercise 2x 24 (twice weekly for 24 weeks) | 75  F96% M4% | 50.9 (12.5) | 30.0 (6.8) | FIQR 57.3 (20.3)  SF36 PCS 30.3 (7.5)  SF36 MCS 39.4 (11.1) | White race: 60.0% | High school or higher education: 96.0% |
|  |  | 12 | Tai Chi 1×12 (once weekly for 12 weeks) | 39  F85% M15% | 53.0 (12.6) | 30.6 (6.4) | FIQR 52.4 (18.7)  SF36 PCS 32.8 (6.6)  SF36 MCS 39.5 (10.5) | White race: 71.8% | High school or higher education: 97.4% |
|  |  | 12 | Tai Chi 2×12 (twice weekly for 12 weeks) | 37  F81% M19% | 52.1 (10.3) | 30.4 (6.8) | FIQR 53.8 (23.3)  SF36 PCS 32.5 (9.7)  SF36 MCS 42 (11.1) | White race: 54.1% | High school or higher education: 94.6% |
|  |  | 24 | Tai Chi 1×24 (once weekly for 24 weeks) | 39  F97% M3% | 50.8 (11.8) | 29.9 (6.4) | FIQR 56.5 (15.5)  SF36 PCS 28.5 (7.3)  SF36 MCS 40.9 (11.9) | White race: 61.5% | High school or higher education: 92.1% |
|  |  | 24 | Tai Chi 2×24 (twice weekly for 24 weeks) | 36  F100% | 52.1 (13.3) | 29.3 (7.4) | FIQR 60.4 (17.8)  SF36 PCS 28.5 (6.5)  SF36 MCS 39.1 (9.8) | White race: 58.3% | High school or higher education: 97.2% |
| Balneotherapy vs. Balneotherapy + Mx Exercise AQ vs. Mx Exercise AQ | Kurt 2016^32^  Ref ID 687 | 3 | Balneotherapy | 40  F100% | 38.1 (10.9) | NR | FIQ 77.6 (11.9) | NR | Illiterate: 16.2%; Primary - secondary: 43.2%; High school: 29.7%; University: 10.8% |
|  |  |  | Balneotherapy + Exercise | 40  F100% | 35.1 (11.6) | NR | FIQ 73.3 (9.9) | NR | Illiterate: 13.9%; Primary - secondary: 55.6%; High school: 27.8%; University: 2.8% |
|  |  |  | Exercise | 40  F100% | 41.9 (12.8) | NR | FIQ 77.1 (10.5) | NR | Illiterate: 16.7%; Primary - secondary: 47.2%; High school: 27.8%; University: 8.3% |
| Education vs. Mind-body Ex LD | Jones 2012 ^33^  Ref ID 210 | 12 | Education | 50  F94% M6% | 54.8 | 30.1 | FIQ 63.6 | White: 95.3% | Some college or higher: 80.9 % |
|  |  |  | Tai Chi | 51  F92% M8% | 53.3 | 30.9 | FIQ 64.1 | White: 98.0% | Some college or higher: 88.2 % |
| Education vs. Mind-body Ex LD | Maddali Bongi 2016^34^  Ref ID 153 | 16 | Control (FMS educational lesson) | 25?  Gender NR | 54.3 (10.6) | NR | FIQ 46.39 (14.46)  SF36 PCS 36.18 (9.27)  SF36 MCS 39.09 (9.59) | NR | NR |
|  |  |  | Tai Ji Quan | 25?  Gender NR | 50.4 (13.7) | NR | FIQ 54.33 (14.61)  SF36 PCS 35.36 (6.7)  SF36 MCS 31.64 (7.45) | NR | NR |
| Education vs. Mind-body Ex LD | Mist 2012^35^  Ref ID 641 | 12 | Education | 50  F94% M6% | 54.8 | 30.1 | FIQ 63.6 | White: 95.3% | Some college or higher: 80.9% |
|  |  |  | Tai chi | 51  F92% M8% | 53.3 | 30.9 | FIQ 64.1 | White: 98.0% | Some college or higher: 88.2% |
| Education vs. Mx Exercise AQ | Fonseca 2021^36^  Ref ID 271 | 11 | Health education | 19  F100% | 54.5 (11.2) | 29.4 (5.1) | FIQ 57.8 (14.8) | NR | NR |
|  |  |  | Aquatic physiotherapy | 27  F100% | 53.8 (10.4) | 27.2 (5.9) | FIQ 58.4 (16.3) | NR | NR |
| Education vs. PT/BT sleep | Martínez 2014^37^  Ref ID 118 | 6 | Sleep hygiene educational program | 32  F100% | 48.66 (7.27) | NR | FIQ 64.09 (13.61) | NR | (n = 29) Basic education 38.1%; High school 14.3%; Professional instruction 28.6%; University studies 19.0% |
|  |  |  | CBT-I | 32  F100% | 46.53 (6.31) | NR | FIQ 60.71 (11.83) | NR | (n = 30) Basic education 21.7%; High school 34.8%; Professional instruction 17.4%; University studies 26.1% |
| Education vs. PT/BT sleep | Miró 2011^38^  Ref ID 2540 | 6 | Sleep hygiene | 22  F100% | 50.2 (6.1) | NR | FIQ 62.19 (13.97) | NR | (n = 15) Basic education: 30%; High school: 16.7%; Professional instruction: 18.0%; University studies: 25.3% |
|  |  |  | CBT-I | 22  F100% | 43.9 (6.1) | NR | FIQ 59.66 (12.83) | NR | (n = 16) Basic education: 32.5%; High school: 21.3%; Professional instruction: 14.5%; University studies: 32.8% |
| Education + Flex/skill LD vs. Mind-body Ex LD | Wang 2010^39^  Ref ID 245 | 12 | Control (wellness education and stretching) | 33  F88% M12% | 50.5 (10.5) | 31.5 (7.4) | FIQR 68 (11)  SF36 PCS 28 (7.8)  SF36 MCS 37.8 (10.5) | White race: 52% | High-school or higher education: 91% |
|  |  |  | Tai Chi | 33  F85% M15% | 49.7 (11.8) | 33.9 (8.9) | FIQR 62.9 (15.5)  SF36 PCS 28.5 (8.4)  SF36 MCS 42.6 (12.2) | White race: 61% | High-school or higher education: 94% |
| Electro T vs. Occlusal SS | Molina-Torres 2016^40^  Ref ID 341 | 12 | Laser therapy | 29  F93% M7% | 51.0 (8.3) | NR | NR | NR | Primary studies: 63.0%; Higher education: 37.0% |
|  |  |  | Occlusal stabilization splint | 29  F97% M3% | 51.8 (7.8) | NR | NR | NR | Primary studies: 67.9%; Higher education: 32.1% |
| Flex/skill AQ vs. Mind-body Ex AQ | Calandre 2009^41^  Ref ID 243 | 6 | Stretching in pool | 39  F87% M13% | 51 (8.0) | NR | NR | NR | NR |
|  |  |  | Tai Chi in pool | 42  F93% M7% | 49 (8.4) | NR | NR | NR | NR |
| Flex/skill LD vs. Aerobic AQ | López-Rodríguez 2013^42^  Ref ID 248 | 12 | Stretching | 38  F100% | 54.8 (7.5) whole pop | NR | FIQ 68.75 (14.6) | NR | NR |
|  |  |  | Aquatic Bio-dance | 38  F100% |  | NR | FIQ 66.09 (11.8) | NR | NR |
| Manual T vs. Non-MSM practice | Castro Sánchez 2019^43^  Ref ID 238 | 4 | Myofascial release | 32  F88% M13% | 46.8 (7.2) | NR | NR | NR | No studies: 9.4%; School level: 43.7%; Bachelor level: 40.6%; University level: 6.3%; |
|  |  |  | Dry needling | 32  F94% M6% | 47.4 (5.0) | NR | NR | NR | No studies: 15.6%; School level: 31.3%; Bachelor level: 37.5%; University level: 15.6%; |
| Nutrition vs. Nutrition | Martínez-Rodríguez 2020^44^  Ref ID 213 | 16 | Control (Mediterranean diet) | 11  F100% | 50 (5) | 28.6 (5.1) | NR | NR | NR |
|  |  |  | Tryptophan- and magnesium-enriched Mediterranean diet | 11  F100% | 48 (4) | 28.2 (3.7) | NR | NR | NR |
| Nutrition vs. Nutrition | Slim 2017^45^  Ref ID 1811 | 24 | Gluten-free diet | 35  F100% | 52 (36-66) median (range) | 27.0 (5.85) | FIQR 69.5 (16.3)  SF12 PCS 28.7 (4.7)  SF12 MCS 31.9 (9.2) | NR | NR |
|  |  |  | Hypocaloric diet | 40  F95% M5% | 53 (32-65) median (range) | 30.2 (5.29) | FIQR 70.4 (16.1)  SF12 PCS 27.1 (5.4)  SF12 MCS 34.4 (12.2) | NR | NR |
| PT/BT gen vs. PT/BT gen | Van Gordon 2017^46^  Ref ID 198 | 8 | CBT | 74  F84% M16% | 47.3 (9.8) | NR | FIQR 54.04 (8.86) | White British: 71.6%; White non-British: 9.5%; Asian: 9.5%; Black Caribbean: 9.5% | School leaver: 59.5%; Vocational: 25.7%; University: 14.9% |
|  |  |  | Meditation awareness training (mindfulness-based intervention) | 74  F82% M18% | 46.4 (9.1) | NR | FIQR 55.24 (10.06) | White British: 77.0%; White non-British: 9.5%; Asian: 8.1%; Black Caribbean: 5.3% | School leaver: 55.4%; Vocational: 25.7%; University: 18.9% |
| PT/BT gen vs. PT/BT sleep | Prados 2020^47^  Ref ID 2269 | 9 | CBT for pain | 19  F100% | 51.2 (5.3) | 27.8 (3.0) | NR | NR | Non-compulsory secondary or higher education: 60.0% |
|  |  |  | CBT for pain and insomnia combined | 20  F100% | 49.0 (9.5) | 26.4 (5.6) | NR | NR | Non-compulsory secondary or higher education: 82.4% |
| Relaxation/Medication vs. Strengthening LD | Ericsson 2016^48^  Ref ID 326 | 15 | Relaxation (active control) | 63  F100% | 52.1 (9.8) | 28.7 (5.3) | NR | NR | ≤9 years: 24 %; 10-12 years: 35 %; >12 years: 41 % |
|  |  |  | Resistance exercise | 67  F100% | 50.8 (9.1) | 27.4 (5.3) | NR | NR | ≤9 years: 12 %; 10-12 years: 51 %; >12 years: 37 %; |
| Studies reporting Medical Outcome Study Sleep Scale (MOS-SS) outcome |  |  |  |  |  |  |  |  |  |
| UC vs. Multidisciplinary (PT/BT gen + Mx Exercise LD + Mx Exercise AQ) | Castel 2013^49^  Ref ID 376 | Unclear (possibly 12w) | Pharmacological treatment | 74  F100% | 48.8 (7.2) | 28.8 (5.8) | FIQ 66.6 (17.4) | 98% of the total sample were Caucasian | NR but the inclusion criteria included between 3 and 8 years of schooling |
|  |  |  | Multidisciplinary (including CBT + physical therapy [aerobic capacity, muscular strengthening, and flexibility, as part of hydro-kinesiotherapy and kinesiotherapy in a gymnasium]) + Pharmacological treatment | 81  F100% | 49.0 (6.8) | 27.6 (4.8) | FIQ 64.6 (16) |  |  |
| UC vs. PT/BT gen | Kong 2021^50^  Ref ID 716 | 8 | Control | 30  F97% M3% | ≤39 y: 13%; 40-49y: 13%; 50-59y: 37%; ≥60y: 37% | NR | NR | NR | ≤Middle: 33.3%; ≤High: 23.3%; ≥College: 43.3% |
|  |  |  | CBT | 30  F93% M7% | ≤39 y: 23%; 40-49y: 13%; 50-59y: 37%; ≥60y: 27% | NR | NR | NR | ≤Middle: 30.0%; ≤High: 36.7%; ≥College: 33.3% |
| UC vs. PT/BT gen | Williams 2010^51^  Ref ID 273 | 24 | Standard care | 59  F95% M5% | 50.8 (10.6) | 29.3 (5.2) | SF36 PCS 38.9 (9.5) | White: 96.6%, Other: 3.4% | Postgraduate degree: 11.9%; College degree: 33.9%; Some college: 37.3%; High school or less: 16.9% |
|  |  |  | Web-enhanced behavioural self-management program + standard care | 59  F95% M5% | 50.2 (12.3) | 28.0 (5.3) | SF36 PCS 38.9 (8.6) | White: 58/59 (98.3%), Other: 1/59 (1.7%) | Postgraduate degree: 11.9%; College degree: 25.4%; Some college: 42.4%; High school or less: 20.3% |
| UC vs. PT/BT gen vs. UC vs. PT/BT gen | Racine 2019^52^  Ref ID 296 | 10 | Delayed operant learning | 36  Gender NR | NR | NR | NR | NR | NR |
|  |  |  | Immediate operant learning | .54  Gender NR | NR | NR | NR | NR | NR |
|  |  |  | Delayed energy conservation | 35  Gender NR | NR | NR | NR | NR | NR |
|  |  |  | Immediate energy conservation | 53  Gender NR | NR | NR | NR | NR | NR |
| UC vs. PT/BT gen vs. PT/BT gen + Relaxation/Medication | Castel 2012^53^  Ref ID 281 | 14 | Pharmacological treatment (standard care) | 30  F100% | 48.7 (6.5) | NR | FIQ 66.1 (3) | White: 100% | Formal Education status. Low: 60%; Middle: 33.3%; High: 6.7% |
|  |  |  | CBT | 34  F94% M6% | 50.0 (7.6) | NR | FIQ 62.7 (2.8) | White: 100% | Formal Education status. Low: 58.8%; Middle: 32.4%; High: 8.8%; |
|  |  |  | CBT + hypnosis | 29  F97% M3% | 50.2 (6.2) | NR | FIQ 69.3 (3) | White: 100% | Formal Education status. Low: 44.8%; Middle: 51.7%; High: 3.4%; |
| UC vs. Relaxation/Medication | Picard 2013^54^  Ref ID 275 | 12 (Tx duration unclear; Ax at 12w) | Wait list | 31  F100% | 49.3 (8.5) | NR | FIQ 50.1 (13.6) | NR | NR |
|  |  |  | Self-hypnosis | 31  F100% | 48.1 (9.3) | NR | FIQ 49.5 (11.6) | NR | NR |
| UC vs. PBO/Sham vs. Relaxation/Medication | Amirova 2017^55^  Ref ID 583 | 4 | Usual care | 58  F91% M9% | 49.0 (10.1) | NR | FIQR 65.5 (16.1)  SF36 16.98 (17.04) | NR | NR |
|  |  |  | Attention control | 66  F95% M5% | 50.5 (10.8) | NR | FIQR 69.83 (20.67)  SF36 15.45 (16.23) | NR | NR |
|  |  |  | Mitchell method relaxation technique online | 67  F94% M6% | 48.1 (11.1) | NR | FIQR 68.09 (20.03)  SF36 20.47 (17.09) | NR | NR |
| PBO/Sham vs. Neuromodulation | Nelson 2010^56^  Ref ID 331 | Unclear (22 sessions) | Sham LENS | 21  F100% | 52.0 (11.4) | NR | FIQ 39.25 (6.46) | Non-Hispanic White: 88.2% | Mean (SD) years: 16.1 (3.1) |
|  |  |  | Low energy neurofeedback system (LENS) | 21  F94% M6% | 51.6 (8.6) | NR | FIQ 44.75 (11.46) | Non-Hispanic White: 88.2% | Mean (SD) years: 15.8 (2.9) |
| Studies reporting Jenkins Sleep Scale (JSS) outcome |  |  |  |  |  |  |  |  |  |
| UC vs. HBOT | Curtis 2021^57^  Ref ID 2323 | 12 (Ax at end of waiting period) | Wait list | 9  F100% | 51.8 (14.5) | 25.0 (4.2) | FIQR 80.1 (6.5) | NR | NR |
|  |  | 8 (Ax immediately after the 8-week Tx period) | Hyperbaric oxygen therapy | 9  F78% M22% | 45.7 (14.2) | 24.9 (5.3) | FIQR 79.7 (6.1) | NR | NR |
| PBO/Sham vs. Electro T | Udina-Corte 2020^58^ Ref ID 2309 | 4 | Sham NAE | 19  F100% | 52 (8) | NR | FIQ 55.5 (13.2)  SF36 PCS 50.5 (2.5)  SF36 MCS 45.9 (3.2) | NR | NR |
|  |  |  | Neuro-adaptive electrostimulation (NAE) | 23  F100% | 52 (9) | NR | FIQ 57.3 (14.6)  SF36 PCS 50.6 (3.6)  SF36 MCS 44.6 (3.2) | NR | NR |
| Flex/skill LD vs. Flex/skill LD + Manual T | Toprak Celenay^59^ 2020  Ref ID 333 | 6 | Spinal stabilization exercise | 21  F100% | 44.0 (10.0) | 27.4 (7.1) median (IQR) | NR | NR | Median (IQR) Years: 12.0 (11.0) |
|  |  |  | Spinal stabilization exercise + kinesio taping | 21  F100% | 38.0 (24.0) | 24.8 (4.6) median (IQR) | NR | NR | Median (IQR) Years: 12.0 (4.0) |
| Studies reporting VAS/NRS on sleep quality |  |  |  |  |  |  |  |  |  |
| UC vs. Mind-body Ex LD | Haak 2008^60^  Ref ID 1609 | 7 | Waiting list control | 28  F100% | 53.4 (8.0) | NR | NR | NR | NR |
|  |  |  | Qigong | 29  F100% | 54.0 (9.4) | NR | NR | NR | NR |
| UC vs. Mind-body Ex LD | Wong 2018^61^  Ref ID 220 | 12 | Control | 19  F100% | 51 (2) | 22.2 (0.6) | NR | NR | NR |
|  |  |  | Tai Chi | 18  F100% | 51 (2) | 23.1 (0.5) | NR | NR | NR |
| UC vs. PT/BT gen | Haugmark 2021^62^  Ref ID 2302 | 12 | TAU | 85  F95% M5% | 41 (24, 51) | NR | EQ5D 0.47 (0.2) | NR | Primary/middle school (1–10 years): 14%; Upper secondary school/vocational 10–12 years: 38%; Bachelor/university >12 years: 48% |
|  |  |  | Multicomponent programme (Norwegian mindfulness-based and acceptance-based programme, the Vitality Training Programme (VTP), and physical activity counselling) | 85  F92% M8% | 44 (26, 52) | NR | EQ5D 0.51 (0.2) | NR | Primary/middle school (1–10 years): 9%; Upper secondary school/vocational 10–12 years: 42%; Bachelor/university >12 years: 47% |
| PBO/Sham vs. Nutrition | Merchant 2001^63^  Ref ID 470 | 12 (Tx 12w, wash-out 4w, then Tx 12w) | Placebo (tablet and liquid) | 43  F95% M5% (cross-over trial) | 46.6 (8.6) | NR | FIQ 54.9 (18.16) | NR | Mean (SD) years: 14.0 (2.2) |
|  |  |  | Dietary supplementation with chlorella extract (tablet and liquid) |  |  |  | FIQ 58.4 (14.9) |  |  |
| PBO/Sham vs. Non-MSM practice | Deluze 1992^64^  Ref ID 218 | 3 | Sham electroacupuncture | 34 F62% M38% | 49.0 (2.0) | NR | NR | NR | NR |
|  |  |  | Electroacupuncture | 36  F92% M8% | 46.8 (2.3) | NR | NR | NR | NR |
| Mind-body Ex LD vs. Mind-body Ex LD | Maddali Bongi^65^ 2012 Ref ID 154 | 7 | Qui Gong | 38  Gender NR  (cross-over trial) | 57.30 (11.5) | NR | FIQ 64.58 (16.54)  SF36 PCS 33.44 (6.46)  SF36 MCS 33.56 (9.9) | NR | NR |
|  |  |  | Rességuier method |  |  |  | FIQ 66.05 (13.5)  SF36 PCS 34 (7.44)  SF36 MCS 36.55 (10.64) |  |  |
| PHARMACOLOGICAL INTERVENTIONS |  |  |  |  |  |  |  |  |  |
| Studies reporting Pittsburgh Sleep Quality Index (PSQI) outcome |  |  |  |  |  |  |  |  |  |
| PBO/Sham vs. Antipsychotics | Potvin 2012^66^  Ref ID 230 | 12 | Placebo + current medication | 26  F100% | 49.1 (8.7) | NR | FIQ 63.8 (13.5) | Caucasian: 100% | NR |
|  |  |  | Quetiapine extended-release as add-on to current medication | 25  F100% | 50.0 (11.7) | NR | FIQ 59.7 (12.4) | Caucasian: 100% | NR |
| PBO/Sham vs. Antioxidant | Di Pierro 2017^67^  Ref ID 202 | 12 | Control (CoQ10-free supplement) | 10 (cross-over trial first phase only) F100% | 53.6 (7.8) | NR | FIQ 61.5 (9.5);  SF36 PCS 31.3 (4.3);  SF36 MCS 36.8 (10.5) | NR | NR |
|  |  |  | Coenzyme Q10 (CoQ10) | 12 (cross-over trial first phase only) F100% | 52.5 (10.4) | NR | FIQ 54.1 (16.6);  SF36 PCS 35.8 (7.7)  SF36 MCS 36.5 (7.5) | NR | NR |
| PBO/Sham vs. CNS depressant | Reuter 2017^68^  Ref ID 310 | 15 | Placebo | 12  F100% | 53.8 (6.9) | NR | FIQ 59.96 (15.39) | NR | NR |
|  |  |  | Oral gamma-hydroxybutyrate | 13  F100% | 55.2 (4.9) | NR | FIQ 60.93 (20) | NR | NR |
| Tricyclics vs. Antipsychotics | Calandre 2014^69^  Ref ID 253 | 16 | Amitriptyline as monotherapy | 45  F96% M4% | 50.6 (8.2) | NR | FIQ 76.6 (12.2) | NR | NR |
|  |  |  | Quetiapine extended-release as monotherapy | 45  F100% | 49.7 (7.9) | NR | FIQ 75.4 (11.4) | NR | NR |
| Tricyclics + PBO/Sham  vs. Endogenous hormone + PBO/Sham vs. Endogenous hormone + Tricyclics | de Zanette 2014^70^  Ref ID 370 | 6 | Amitriptyline + placebo | 21  F100% | 49.8 (8.9) | 27.6 (3.9) | FIQ 53.78 (12.83) | NR | Mean (SD) years: 10.9 (5.1) |
|  |  |  | Melatonin + placebo | 21  F100% | 47.4 (7.8) | 27.2 (4.0) | FIQ 64.87 (12.83) | NR | Mean (SD) years: 11.3 (3.8) |
|  |  |  | Melatonin + amitriptyline | 21  F100% | 49.7 (7.2) | 27.6 (4.6) | FIQ 65.15 (9.94) | NR | Mean (SD) years: 8.2 (5.6) |
| Studies reporting Medical Outcome Study Sleep Scale (MOS-SS) outcome |  |  |  |  |  |  |  |  |  |
| PBO/Sham vs. Gabapentinoids | Arnold 2007^71^  Ref ID 377 | 12 | Placebo | 75  F87% M13% | 47.3 (11.8) | NR | FIQ 47.7 (10.3) | White: 97.3%; African American: 1.3%; Asian: 0; Other: 1.3% | NR |
|  |  |  | Gabapentin 1200-2400 mg/d | 75  F93% M7% | 49.2 (10.6) | NR | FIQ 46.3 (11.5) | White: 97.3%; African American: 1.3%; Asian: 1.3%; Other: 0 | NR |
| PBO/Sham vs. Gabapentinoids | Arnold 2014^72^  Ref ID 146 | 13 | Placebo | 58  F90% M10% | 49.3 (12.7) | NR | FIQ 57.0 (13.8)  SF36 PCS 32.2 (8)  SF36 MCS 44.1 (11.6)  (whole pop) | White: 89.7%; Black: 5.2%; Asian: 5.2%; Other: 0 | NR |
|  |  |  | Pregabalin 165 mg | 63  F92% M8% | 50.3 (12.1) | NR |  | White: 90.5%; Black: 4.8%; Asian: 4.8%; Other: 0 | NR |
| PBO/Sham vs. Gabapentinoids | Ohta 2012^73^  Ref ID 222 | 15 | Placebo | 250  F88% M13% | 46.7 (12.6) | NR | FIQ 51.6 (15) | NR | NR |
|  |  |  | Pregabalin 300 or 450 mg | 251  F90% M10% | 47.9 (12.0) | NR | FIQ 52.7 (15.3) | NR | NR |
| PBO/Sham vs. Gabapentinoids vs. Gabapentinoids vs. Gabapentinoids | Arnold 2008*^74^  Ref ID 368 | 14 | Placebo | 184  F92% M8% | 49 (11.4) | NR | FIQ 58.7 (15.6)  SF36 PCS 31.9 (7.3)  SF36 MCS 43.4 (12.9) | White: 91.8%; Black: 3.8%; Other: 4.3% | NR |
|  |  |  | Pregabalin 300 mg/d | 183  F95% M5% | 49.1 (11.2) | NR | FIQ 61.1 (15.7)  SF36 PCS 31.3 (8.6)  SF36 MCS 41.6 (12.7) | White: 89.6%; Black: 4.9%; Other: 5.5% | NR |
|  |  |  | Pregabalin 450 mg/d | 190  F96% M4% | 50.8 (11.8) | NR | FIQ 59.6 (15.1)  SF36 PCS 31.2 (8.3)  SF36 MCS 43.6 (12.4) | White: 90.0%; Black: 6.3%; Other: 3.7% | NR |
|  |  |  | Pregabalin 600 mg/d | 188  F95% M5% | 50.9 (11.1) | NR | FIQ 59.5 (16.2)  SF36 PCS 32.7 (8.5)  SF36 MCS 41.7 (13.6) | White: 92.6%; Black: 2.7%; Other: 9 (4.8% | NR |
| PBO/Sham vs. Gabapentinoids vs. Gabapentinoids vs. Gabapentinoids | Crofford 2005*^75^  Ref ID 260 | 8 | Placebo | 131  F91% M9% | 49.7 (10.7) | NR | NR | White: 95.4% | NR |
|  |  |  | Pregabalin 150 mg/d | 132  F96% M4% | 48.0 (10.4) | NR | NR | White: 93.2% | NR |
|  |  |  | Pregabalin 300 mg/d | 134  F90% M10% | 47.7 (10.1) | NR | NR | White: 91.8% | NR |
|  |  |  | Pregabalin 450 mg/d | 132  F90% M10% | 48.9 (11.3) | NR | NR | White: 92.4% | NR |
| PBO/Sham vs. Gabapentinoids vs. Gabapentinoids vs. Gabapentinoids | Mease 2008*^76^  Ref ID 2539 | 13 | Placebo | 190  F96% M4% | 48.6 (11.3) | 30 | FIQ 64.3 (13.6)  SF36 PCS 31.5 (8.0)  SF36 MCS 39.6 (13.0)  (whole pop) | Caucasian: 87.9%; Black: 5.3%; Hispanic: 6.3%; Other: 0.5% | NR |
|  |  |  | Pregabalin 300 mg/d | 185  F94% M6% | 50.1 (10.4) | 31.4 |  | Caucasian: 91.4%; Black: 5.4%; Hispanic: 92.7%; Other: 90.5% | NR |
|  |  |  | Pregabalin 450 mg/d | 183  F92% M8% | 47.7 (10.8) | 30.2 |  | Caucasian: 92.3%; Black: 3.8%; Hispanic: 3.8%; Other: 0 | NR |
|  |  |  | Pregabalin 600 mg/d | 190  F95% M5% | 48.7 (11.2) | 30.5 |  | Caucasian: 89.5%; Black: 4.2%; Hispanic: 4.7%; Other: 1.6% | NR |
| PBO/Sham vs. Gabapentinoids  vs. SRI  vs. Gabapentinoids + SRI | Gilron 2016^77^  Ref ID 157 | 6 | Placebo | 41  F88% M12% (cross-over trial) | 56 (20-71) median (range) | NR | NR | Caucasian: 98% | NR |
|  |  |  | Pregabalin |  |  |  | NR |  |  |
|  |  |  | Duloxetine |  |  |  | NR |  |  |
|  |  |  | Pregabalin + duloxetine |  |  |  | NR |  |  |
| PBO/Sham vs. Iron replacement | Boomershine 2018^78^  Ref ID 626 | 5 days | Placebo | 40  F100% | 43.9 (10.8) | NR | FIQR 83.1 (9.4) | White: 75%; Black: 17.5%; Hispanic: 5.0%; Asian: 0; Other: 2.5% | NR |
|  |  |  | Ferric carboxymaltose | 41  F98% M3% | 41.2 (11.1) | NR | FIQR 82.8 (9.5) | White: 80.5%; Black: 17.1%; Hispanic: 0; Asian: 2.4%; Other: 0 | NR |
| PBO/Sham vs. SRI | Arnold 2010^79^  Ref ID 350 | 8 | Placebo | 133  F90% M10% | 50.1 (range 20-84) | NR | FIQ 62.76 (SE 1.17)  SF36 PCS 31.29 (SE 0.67))  SF36 MCS 39.42 (SE 1.04) | White: 89.5%; Black: 6.0%; Asian: 0; Other: 4.5% | NR |
|  |  |  | Es-reboxetine | 134  F89% M11% | 49.2 (range 21-79) | NR | FIQ 61.37 (SE 1.40)  SF36 PCS 30.5 (SE 0.68)  SF36 MCS 41.47 (SE 1.13) | White: 87.3%; Black: 4.5%; Asian: 0.7%; Other: 7.5% | NR |
| PBO/Sham vs. SRI | Ahmed 2016^80^  Ref ID 223 | 5 | Placebo | 19 ‘Predominantly women and white’ (89.5%)  (cross-over trial) | 49.2 (range 28–72) | NR | FIQ 56.7 (12.6) | NR | NR |
|  |  |  | Milnacipran |  |  |  |  |  |  |
| PBO/Sham vs. SRI | Branco 2010^81^  Ref ID 1522 | 16 | Placebo | 449  F94% M6% | 49.2 (10.3) | 26.7 (5.0) | FIQ 57 (11.8)  SF36 PCS 33.7 (6.8)  SF36 MCS 46.3 (9.8) | NR | NR |
|  |  |  | Milnacipran 200 mg/d | 435  F95% M5% | 48.3 (9.3) | 26.7 (5.4) | FIQ 56.7 (11.9)  SF36 PCS 33.4 (6.7)  SF36 MCS 47 (9.8) | NR | NR |
| Ultrasound T + Manual T vs. SSRI | González-Viejo^82^ 2005 Ref ID 279 | 24 (Tx 3w; Ax at 24w) | Ultrasonography plus physical therapy | 34  F100% | 46.8 (8.4) | NR | NR | NR | NR |
|  |  | 24 (Tx 24w) | Sertraline, 50 mg/24h | 36  F100% | 45.2 (7.2) | NR | NR | NR | NR |
| Studies reporting Jenkins Sleep Scale (JSS) outcome |  |  |  |  |  |  |  |  |  |
| PBO/Sham vs. CNS depressant | Moldofsky 2010^83^  Ref ID 147 | 8 | Placebo | 66 | 46.5 (11.3) whole pop | NR | NR | Caucasian: 92% of whole pop | NR |
|  |  |  | Sodium oxybate 4.5 g | 62 |  |  | NR |  |  |
|  |  |  | Sodium oxybate 6 g | 67  (F94 M6% whole pop) |  |  | NR |  |  |
| PBO/Sham vs. CNS depressant | Spaeth 2012^84^  Ref ID 122 | 14 | Placebo | 188  F89% M11% | 46.8 (9.7) | 27.4 (4.7) | FIQ 63.7 (14.1)  SF36 PCS 29.1 (7.9)  EQ-5D 46.6 (22.95) | White: 92.0%; Black: 5.3%; Other: 2.7% | NR |
|  |  |  | Sodium oxybate 4.5 g | 195  F90% M10% | 46.6 (10.8) | 27.4 (4.3) | FIQ 62.3 (15.2)  SF36 PCS 29.6 (8.7)  EQ-5D 48.8 (22.25) | White: 93.3%; Black: 5.6%; Other: 1.0% | NR |
|  |  |  | Sodium oxybate 6 g | 190  F89% M11% | 46.4 (11.4) | 28.0 (4.8) | FIQ 62.1 (15.1)  SF36 PCS 30.8 (7.5)  EQ-5D 47.4 (24) | White: 88.9%; Black: 9.5%; Other: 1.6% | NR |
| PBO/Sham vs. CNS depressant | Russell 2011^85^  Ref ID 228 | 14 | Placebo | 183  F91% M9% | 46.5 (11.4) | 28.9 (5.1) | FIQ 63.3 (13)  SF36 PCS 30.6 (8.4)  SF36 MCS 46.8 (11.3) | White: 91.3%; Black: 4.4%; Other: 4.4% | NR |
|  |  |  | Sodium oxybate 4.5 g | 182  F91% M9% | 47.0 (11.8) | 28.1 (4.6) | FIQ 63 (13.1)  SF36 PCS 29.2 (8.5)  SF36 MCS 48.7 (10.3) | White: 90.1%; Black: 7.1%; Other: 2.7% | NR |
|  |  |  | Sodium oxybate 6 g | 183  F91% M9% | 47.5 (10.6) | 28.4 (4.6) | FIQ 62.4 (14.1)  SF36 PCS 30.2 (8.4)  SF36 MCS 48.4 (11.1) | White: 91.3%; Black: 6.6%; Other: 2.2% | NR |
| PBO/Sham vs. SRI | Vitton 2004^86, 87^  Ref ID 328 | 12 | Placebo | 28  (F96-98% whole pop) | Range 46.2 to 48.0, whole pop | NR | NR | Caucasian: 79% to 89% of whole pop | NR |
|  |  |  | Milnacipran 25 mg QD (single daily dose) | 46 |  | NR | NR |  | NR |
|  |  |  | Milnacipran 12.5 mg BID (two divided doses) | 51 |  | NR | NR |  | NR |
| PBO/Sham vs. TeCAs vs. TeCAs | Yeephu 2013  Ref ID 340 | 13 | Placebo | 13  F100% M0% | 47.4 (10.5) | 22.6 (3.4) | FIQ 45.17 | NR | NR |
|  |  |  | Mirtazapine 15 mg | 13  F100% M0% | 42.7 (12.6) | 22.0 (2.5) | FIQ 39.29 | NR | NR |
|  |  |  | Mirtazapine 30 mg | 14  F100% M0% | 43.9 (9.4) | 22.1 (3.2) | FIQ 44.13 | NR | NR |
| Studies reporting Fibromyalgia Sleep Diary (FMSD) outcome |  |  |  |  |  |  |  |  |  |
| PBO/Sham vs. ASP0819 | Arnold 2020^88^  Ref ID 2315 | 8 | Placebo | 95  F95% M5% | 49.8 (12.5) | 32.2 (6.2) | FIQR 55.99 (12) | White: 77.7%; Black or African American: 17.0%; Asian: 1.1%; American Indian or Alaskan Native: 3.2%; Native Hawaiian or other Pacific Islander: 1.1%; Other: 0 Not Hispanic or Latino: 86.2%; Hispanic or Latio: 13.8% | NR |
|  |  |  | ASP0819 | 91  F98% M2% | 48.7 (12.1) | 31.8 (6.6) | FIQR 56.12 (13.17) | White: 86.7%; Black or African American: 11.1%; Asian: 0; American Indian or Alaskan Native: 0; Native Hawaiian or other Pacific Islander: 1.1%; Other: 1.1%; Not Hispanic or Latino: 88.9%; Hispanic or Latino: 11.1% | NR |
| Studies reporting Sleep Quality Numerical Rating Scale (SQ-NRS) outcome |  |  |  |  |  |  |  |  |  |
| PBO/Sham vs. Gapapentinoids vs. Gapapentinoids vs. Gapapentinoids | Pauer 2011^89^  Ref ID 148 | 14 | Placebo | 184  F91% M9% | 48.1 (11.3) | NR | FIQ 62.58 (14.85) | White: 76.6%; Black: 0; Hispanic: 13.0%; Other: 10.3% | NR |
|  |  |  | Pregabalin 300 mg/d | 184  F90% M10% | 48.4 (10.8) | NR | FIQ 60.69 (14.78) | White: 77.7%; Black: 0.5%; Hispanic: 11.4%; Other: 10.3% | NR |
|  |  |  | Pregabalin 450 mg/d | 182  F92% M8% | 48.0 (11.3) | NR | FIQ 60.59 (14.59) | White: 74.7%; Black: 0; Hispanic: 13.2%; Other: 12.1% | NR |
|  |  |  | Pregabalin 600 mg/d | 186  F90% M10% | 49.6 (11.3) | NR | FIQ 60.4 (13.87) | White: 74.7%; Black: 0; Hispanic: 12.4%; Other: 12.9% | NR |
| Studies reporting VAS/NRS on sleep quality |  |  |  |  |  |  |  |  |  |
| PBO/Sham vs. Endogenous hormones | Mameli 2014^90^  Ref ID 292 | 3 | Placebo | 14  F100%  (cross-over trial) | 51.9 (7.8) | NR | SF12 22.5 (6.3) | NR | Junior: 21.4%; Secondary: 35.7%; High: 42.9%; |
|  |  |  | Oxytocin |  |  |  | SF12 23.7 (5.8) |  |  |

**Abbreviation:** ACT, acceptance and commitment therapy; AP, antipsychotics; AQ, aquatic or pool-based; Ax, assessment; BID, twice daily; CBT, cognitive behavioural therapy; CNS depressants, central nervous system depressants; DLPFC, dorsolateral prefrontal cortex; Electro T, electrotherapy; FIQ, Fibromyalgia Impact Questionnaire; FIQR, Revised Fibromyalgia Impact Questionnaire; Flex/skill, Flexibility/neuro-motor skills exercise; FMS, fibromyalgia syndrome; LD, land-based; Manual T, manual therapy; Mind-body Ex, mind-body exercise; Mx Exercise, mix (aerobic and anaerobic) exercise; Non-MSM practice, non-mainstream practice; NR, not reported; NRS, numerical rating scale; OIC, operculo-insular cortex; PBO/Sham, placebo or sham treatment; PT/BT gen, psychological or behavioural therapy generic; PT/BT sleep, psychological or behavioural therapy, sleep-focused; QD, once daily; Ref ID, reference ID; rTMS, repetitive transcranial magnetic stimulation; SE, standard error; SERM, selective oestrogen receptor modulators; SRI, serotonin reuptake inhibitors; SF36 MCS, SF-36 mental component score; SF36 PCS, SF-36 physical component score; SSRI, selective serotonin reuptake inhibitors; SXB, sodium oxybate; TAU, treatment as usual; repetitive transcranial magnetic stimulation; TCA, tricyclics, or tricyclic antidepressant; tDCS = transcranial direct current stimulation; TeCA, tetracyclic antidepressants; TENS, transcutaneous electrical nerve stimulation; Tx, treatment; UC, usual care; VAS, visual analogue scale; w, weeks; * This study also reports SQ-NRS outcome.

**References**

1. Maindet C, Maire A, Vermorel C, et al. Spa therapy for the treatment of fibromyalgia: an open, randomized multicenter trial. J Pain. 2021;**8**:940-51.

2. Ceca D, Pablos A, Elvira L, et al. Effectiveness of a self-myofascial conditioning programme on pain, depression, anxiety and sleep quality in people with Fibromyalgia. Cuadernos de Psicologia del Deporte. 2020;**20**(1):147-65.

3. Castro-Sánchez AM, Aguilar-Ferrándiz ME, Matarán-Peñarrocha GA, et al. Short-term effects of a manual therapy protocol on pain, physical function, quality of sleep, depressive symptoms, and pressure sensitivity in women and men with fibromyalgia syndrome: a randomized controlled trial. Clin J Pain. 2014;**30**(7):589‐97.

4. Jiao J, Russell IJ, Wang W, et al. Ba-Duan-Jin alleviates pain and fibromyalgia-related symptoms in patients with fibromyalgia: results of a randomised controlled trial. Clin Exp Rheumatol. 2019;**37**(6):953‐62.

5. Lynch M, Sawynok J, Hiew C, et al. A randomized controlled trial of qigong for fibromyalgia. Arthritis Res Ther. 2012;**14**(4):R178.

6. Munguía-Izquierdo D, Legaz-Arrese A. Assessment of the effects of aquatic therapy on global symptomatology in patients with fibromyalgia syndrome: a randomized controlled trial. Arch Phys Med Rehabil. 2008;**89**(12):2250‐7.

7. Lauche R, Spitzer J, Schwahn B, et al. Efficacy of cupping therapy in patients with the fibromyalgia syndrome-a randomised placebo controlled trial. Sci Rep. 2016;**6**:37316.

8. San Mauro Martin I, Lopez Oliva S, Collado Yurrita L, et al. Anti-inflammatory and antioxidant feeding and supplementation may serve as adjuvants in women with fibromyalgia. J Nutr Intermed Metab. 2019;**15**:3‐9.

9. Barmaki M, Maindet-Dominici C, Nizard J, et al. Multicenter, prospective, controlled double-blind study comparing Fib-19-01, a phytotherapy treatment, to a dietary supplement and to conventional care in patients suffering from fibromyalgia. Altern Ther Health Med. 2019;**25**(4):46‐53.

10. Amutio A, Franco C, Sanchez-Sanchez LC, et al. Effects of mindfulness training on sleep problems in patients with fibromyalgia. Front Psychol. 2018;**9**.

11. Simister HD, Tkachuk GA, Shay BL, et al. Rrandomized controlled trial of online acceptance and commitment therapy for fibromyalgia. J Pain. 2018;**19**(7):741‐53.

12. Schmidt S, Grossman P, Schwarzer B, et al. Treating fibromyalgia with mindfulness-based stress reduction: results from a 3-armed randomized controlled trial. Pain. 2011;**152**(2):361‐9.

13. Lami MJ, Martinez MP, Miro E, et al. Efficacy of combined cognitive-behavioral therapy for insomnia and pain in patients with fibromyalgia: a randomized controlled trial. Cognit Ther Res. 2018;**42**(1):63‐79.

14. Onieva-Zafra MD, Parra-Fernandez ML, Fernandez-Martinez E. Benefits of a home treatment program using guided imagery relaxation based on audio recordings for people with fibromyalgia. Holist Nurs Pract. 2019;**33**(2):111‐20.

15. Senna MK, Sallam RA, Ashour HS, et al. Effect of weight reduction on the quality of life in obese patients with fibromyalgia syndrome: a randomized controlled trial. Clin Rheumatol. 2012;**31**(11):1591‐7.

16. Arcos-Carmona IM, Castro-Sánchez AM, Matarán-Peñarrocha GA, et al. Effects of aerobic exercise program and relaxation techniques on anxiety, quality of sleep, depression, and quality of life in patients with fibromyalgia: a randomized controlled trial. Med Clin (Barc). 2011;**137**(9):398‐401.

17. Castro-Sanchez AM, Mataran-Pearrocha GA, Granero-Molina J, et al. Benefits of massage-myofascial release therapy on pain, anxiety, quality of sleep, depression, and quality of life in patients with fibromyalgia. Evid Based Complement Alternat Med. 2011;**2011**.

18. Nadal-Nicolás Y, Rubio-Arias JÁ, Martínez-Olcina M, et al. Effects of manual therapy on fatigue, pain, and psychological aspects in women with fibromyalgia. Int J Environ Res Public Health. 2020;**17**(12):1-14.

19. Ide MR, Laurindo LMM, Rodrigues-Junior AL, et al. Effect of aquatic respiratory exercise-based program in patients with fibromyalgia. Int J Rheum Dis. 2008;**11**(2):131‐40.

20. Liu W, Zahner L, Cornell M, et al. Benefit of Qigong exercise in patients with fibromyalgia: a pilot study. Int J Neurosci. 2012;**122**(11):657‐64.

21. Sarmento CVM, Moon S, Pfeifer T, et al. The therapeutic efficacy of Qigong exercise on the main symptoms of fibromyalgia: a pilot randomized clinical trial. Integr Med Res. 2020;**9**(4).

22. Guinot M, Maindet C, Hodaj H, et al. Effects of repetitive transcranial magnetic stimulation and multicomponent therapy in patients with fibromyalgia: a randomized controlled trial. Arthritis Care Res. 2021;**73**(3):449-58.

23. Moustafa IM, Diab AA. The addition of upper cervical manipulative therapy in the treatment of patients with fibromyalgia: a randomized controlled trial. Rheumatol Int. 2015;**35**(7):1163‐74.

24. Goldway N, Ablin J, Lubin O, et al. Volitional limbic neuromodulation exerts a beneficial clinical effect on Fibromyalgia. NeuroImage. 2019;**186**:758‐70.

25. Samartin-Veiga N, Pidal-Miranda M, Gonzalez-Villar AJ, et al. Transcranial direct current stimulation of three cortical targets is no more effective than placebo as treatment for fibromyalgia: a double-blind sham-controlled clinical trial. Pain. 2021.

26. Wu YL, Fang SC, Chen SC, et al. Effects of neurofeedback on fibromyalgia: a randomized controlled trial. Pain Manag Nurs. 2021.

27. Mirzaei A, Zabihiyeganeh M, Jahed SA, et al. Effects of vitamin D optimization on quality of life of patients with fibromyalgia: A randomized controlled trial. Med J Islam Repub Iran. 2018;**32**:29.

28. Mataran-Penarrocha GA, Castro-Sanchez AM, Garcia GC, et al. Influence of craniosacral therapy on anxiety, depression and quality of life in patients with fibromyalgia. Evid Based Complement Alternat Med. 2011;**2011**.

29. Gómez-Hernández M, Gallego-Izquierdo T, Martínez-Merinero P, et al. Benefits of adding stretching to a moderate-intensity aerobic exercise programme in women with fibromyalgia: a randomized controlled trial. Clinical rehabilitation. 2020;**34**(2):242‐51.

30. de Medeiros SA, de Almeida Silva HJ, do Nascimento RM, et al. Mat Pilates is as effective as aquatic aerobic exercise in treating women with fibromyalgia: a clinical, randomized and blind trial. Adv Rheumatol. 2020;**60**(1):21.

31. Wang C, Schmid CH, Fielding RA, et al. Effect of tai chi versus aerobic exercise for fibromyalgia: comparative effectiveness randomized controlled trial. BMJ (Clinical research ed). 2018;**360**:k851.

32. Kurt EE, Kocak FA, Erdem HR, et al. Which non-pharmacological treatment is more effective on clinical parameters in patients with fibromyalgia: balneotherapy or aerobic exercise? Arch Rheumatol. 2016;**31**(2):162‐9.

33. Jones KD, Sherman CA, Mist SD, et al. A randomized controlled trial of 8-form Tai chi improves symptoms and functional mobility in fibromyalgia patients. Clin Rheumatol. 2012;**31**(8):1205‐14.

34. Maddali Bongi S, Paoletti G, Calà M, et al. Efficacy of rehabilitation with Tai Ji Quan in an Italian cohort of patients with Fibromyalgia Syndrome. Complement Ther Clin Pract. 2016;**24**:109‐15.

35. Mist S, Jones K, Sherman C, et al. A randomized controlled trial of 8-form Tai chi improves symptoms and functional mobility in fibromyalgia patients. BMC Complement Altern Med. 2012;**12**.

36. Fonseca ACS, Faria PC, Alcântara MA, et al. Effects of aquatic physiotherapy or health education program in women with fibromyalgia: a randomized clinical trial. Physiother Theory Pract. 2021;**37**(5):620‐32.

37. Martínez MP, Miró E, Sánchez AI, et al. Cognitive-behavioral therapy for insomnia and sleep hygiene in fibromyalgia: a randomized controlled trial. J Behav Med. 2014;**37**(4):683‐97.

38. Miró E, Lupiáñez J, Martínez MP, et al. Cognitive-behavioral therapy for insomnia improves attentional function in fibromyalgia syndrome: a pilot, randomized controlled trial. J Health Psychol. 2011;**16**(5):770-82.

39. Wang C, Schmid CH, Rones R, et al. A randomized trial of tai chi for fibromyalgia. N Engl J Med. 2010;**363**(8):743‐54.

40. Molina-Torres G, Rodríguez-Archilla A, Matarán-Peñarrocha G, et al. Laser therapy and occlusal stabilization splint for temporomandibular disorders in patients with fibromyalgia syndrome: a randomized, clinical trial. Altern Ther Health Med. 2016;**22**(5):23‐31.

41. Calandre EP, Rodriguez-Claro ML, Rico-Villademoros F, et al. Effects of pool-based exercise in fibromyalgia symptomatology and sleep quality: a prospective randomised comparison between stretching and Ai Chi. Clin Exp Rheumatol. 2009;**27**(5 Suppl 56):S21‐8.

42. López-Rodríguez MM, Fernández-Martínez M, Matarán-Peñarrocha GA, et al. Effectiveness of aquatic biodance on sleep quality, anxiety and other symptoms in patients with fibromyalgia. Med Clin (Barc). 2013;**141**(11):471‐8.

43. Castro Sánchez AM, García López H, Fernández Sánchez M, et al. Improvement in clinical outcomes after dry needling versus myofascial release on pain pressure thresholds, quality of life, fatigue, pain intensity, quality of sleep, anxiety, and depression in patients with fibromyalgia syndrome. Disabil Rehabil. 2019;**41**(19):2235‐46.

44. Martínez-Rodríguez A, Rubio-Arias J, Ramos-Campo DJ, et al. Psychological and sleep effects of tryptophan and magnesium-enriched Mediterranean diet in women with fibromyalgia. Int J Environ Res Public Health. 2020;**17**(7).

45. Slim M, Calandre EP, Garcia-Leiva JM, et al. The effects of a gluten-free diet versus a hypocaloric diet among patients with fibromyalgia experiencing gluten sensitivity-like symptoms. J Clin Gastroenterol. 2017;**51**(6):500-7.

46. Van Gordon W, Shonin E, Dunn TJ, et al. Meditation awareness training for the treatment of fibromyalgia syndrome: a randomized controlled trial. Br J Health Psychol. 2017;**22**(1):186‐206.

47. Prados G, Miro E, Martinez MP, et al. Combined cognitive-behavioral therapy for fibromyalgia: Effects on polysomnographic parameters and perceived sleep quality. Int J Clin Health Psychol. 2020;**20**(3):232-42.

48. Ericsson A, Palstam A, Larsson A, et al. Resistance exercise improves physical fatigue in women with fibromyalgia: a randomized controlled trial. Arthritis Res Ther. 2016;**18**:176.

49. Castel A, Fontova R, Montull S, et al. Efficacy of a multidisciplinary fibromyalgia treatment adapted for women with low educational levels: a randomized controlled trial. Arthritis Care Res. 2013;**65**(3):421‐31.

50. Kong KR, Lee EN. Effects of a cognitive behavior therapy program for patients with fibromyalgia syndrome: a randomized controlled trial. J Korean Acad Nurs. 2021;**51**(3):347‐62.

51. Williams DA, Kuper D, Segar M, et al. Internet-enhanced management of fibromyalgia: a randomized controlled trial. Pain. 2010;**151**(3):694‐702.

52. Racine M, Jensen MP, Harth M, et al. Operant learning versus energy conservation activity pacing treatments in a sample of patients with fibromyalgia syndrome: a pilot randomized controlled trial. J Pain. 2019;**20**(4):420‐39.

53. Castel A, Cascón R, Padrol A, et al. Multicomponent cognitive-behavioral group therapy with hypnosis for the treatment of fibromyalgia: long-term outcome. J Pain. 2012;**13**(3):255‐65.

54. Picard P, Jusseaume C, Boutet M, et al. Hypnosis for management of fibromyalgia. Int J Clin Health Psychol. 2013;**61**(1):111‐23.

55. Amirova A, Cropley M, Theadom A. The effectiveness of the Mitchell Method Relaxation Technique for the treatment of fibromyalgia symptoms: a three-arm randomized controlled trial. Int J Stress Manag. 2017;**24**(1):86‐106.

56. Nelson DV, Bennett RM, Barkhuizen A, et al. Neurotherapy of fibromyalgia? Pain Med. 2010;**11**(6):912‐9.

57. Curtis K, Katz J, Djaiani C, et al. Evaluation of a hyperbaric oxygen therapy intervention in individuals with fibromyalgia. Pain Med. 2021;**22**(6):1324-32.

58. Udina-Cortes C, Fernandez-Carnero J, Romano AA, et al. Effects of neuro-adaptive electrostimulation therapy on pain and disability in fibromyalgia A prospective, randomized, double-blind study. Medicine. 2020;**99**(51).

59. Toprak Celenay S, Mete O, Akan S, et al. Comparison of the effects of stabilization exercise plus kinesio taping and stabilization exercise alone on pain and well-being in fibromyalgia. Complement Ther Clin Pract. 2020;**38**:101076.

60. Haak T, Scott B. The effect of Qigong on Fibromyalgia (FMS): A controlled randomized study. Disabil Rehabil. 2008;**30**(8):625-33.

61. Wong A, Figueroa A, Sanchez-Gonzalez MA, et al. Effectiveness of Tai Chi on cardiac autonomic function and symptomatology in women with fibromyalgia: a randomized controlled trial. J Aging Phys Act. 2018;**26**(2):214‐21.

62. Haugmark T, Hagen KB, Provan SA, et al. Effects of a mindfulness-based and acceptance-based group programme followed by physical activity for patients with fibromyalgia: a randomised controlled trial. BMJ Open. 2021;**11**(6):e046943.

63. Merchant RE, Andre CA, Wise CM. Nutritional supplementation with Chlorella pyrenoidosa for fibromyalgia syndrome: a double-blind, placebo-controlled, crossover study. J Musculoskelet Pain. 2001;**9**(4):37‐54.

64. Deluze C, Bosia L, Zirbs A, et al. Electroacupuncture in fibromyalgia: results of a controlled trial. BMJ (Clinical research ed). 1992;**305**(6864):1249‐52.

65. Maddali Bongi S, Del Rosso A, Di Felice C, et al. Rességuier method and Qi Gong sequentially integrated in patients with fibromyalgia syndrome. Clin Exp Rheumatol. 2012;**30**(6 Suppl 74):51‐8.

66. Potvin S, Morin M, Cloutier C, et al. Add-on treatment of quetiapine for fibromyalgia: a pilot, randomized, double-blind, placebo-controlled 12-week trial. J Clin Psychopharmacol. 2012;**32**(5):684‐7.

67. Di Pierro F, Rossi A, Consensi A, et al. Role for a water-soluble form of CoQ10 in female subjects affected by fibromyalgia. A preliminary study. Clin Exp Rheumatol. 2017;**35 Suppl 105**(3):20‐7.

68. Reuter E, Tafelski S, Thieme K, et al. Treatment of fibromyalgia syndrome with gamma-hydroxybutyrate : a randomized controlled study. Schmerz (Berlin, Germany). 2017;**31**(2):149‐58.

69. Calandre EP, Rico-Villademoros F, Galán J, et al. Quetiapine extended-release (Seroquel-XR) versus amitriptyline monotherapy for treating patients with fibromyalgia: a 16-week, randomized, flexible-dose, open-label trial. Psychopharmacology. 2014;**231**(12):2525‐31.

70. de Zanette SA, Vercelino R, Laste G, et al. Melatonin analgesia is associated with improvement of the descending endogenous pain-modulating system in fibromyalgia: a phase II, randomized, double-dummy, controlled trial. BMC Pharmacol Toxicol. 2014;**15**:40.

71. Arnold LM, Goldenberg DL, Stanford SB, et al. Gabapentin in the treatment of fibromyalgia: a randomized, double-blind, placebo-controlled, multicenter trial. Arthritis Rheum. 2007;**56**(4):1336‐44.

72. Arnold LM, Arsenault P, Huffman C, et al. Once daily controlled-release pregabalin in the treatment of patients with fibromyalgia: a phase III, double-blind, randomized withdrawal, placebo-controlled study. Curr Med Res Opin. 2014;**30**(10):2069‐83.

73. Ohta H, Oka H, Usui C, et al. A randomized, double-blind, multicenter, placebo-controlled phase III trial to evaluate the efficacy and safety of pregabalin in Japanese patients with fibromyalgia. Arthritis Res Ther. 2012;**14**(5):R217.

74. Arnold LM, Russell IJ, Diri EW, et al. A 14-week, randomized, double-blinded, placebo-controlled monotherapy trial of pregabalin in patients with fibromyalgia. J Pain. 2008;**9**(9):792‐805.

75. Crofford LJ, Rowbotham MC, Mease PJ, et al. Pregabalin for the treatment of fibromyalgia syndrome: results of a randomized, double-blind, placebo-controlled trial. Arthritis Rheum. 2005;**52**(4):1264‐73.

76. Mease PJ, Russell IJ, Arnold LM, et al. A randomized, double-blind, placebo-controlled, phase III trial of pregabalin in the treatment of patients with fibromyalgia. J Rheumatol. 2008;**35**(3):502-14.

77. Gilron I, Chaparro LE, Tu D, et al. Combination of pregabalin with duloxetine for fibromyalgia: a randomized controlled trial. Pain. 2016;**157**(7):1532‐40.

78. Boomershine CS, Koch TA, Morris D. A blinded, randomized, placebo-controlled study to investigate the efficacy and safety of ferric carboxymaltose in iron-deficient patients with fibromyalgia. Rheumatol Ther. 2018;**5**(1):271‐81.

79. Arnold LM, Chatamra K, Hirsch I, et al. Safety and efficacy of esreboxetine in patients with fibromyalgia: an 8-week, multicenter, randomized, double-blind, placebo-controlled study. Clin Ther. 2010;**32**(9):1618‐32.

80. Ahmed M, Aamir R, Jishi Z, et al. The effects of milnacipran on sleep disturbance in fibromyalgia: a randomized, double-blind, placebo-controlled, two-way crossover study. J Clin Sleep Med. 2016;**12**(1):79‐86.

81. Branco JC, Zachrisson O, Perrot S, et al. A European multicenter randomized double-blind placebo-controlled monotherapy clinical trial of milnacipran in treatment of fibromyalgia. J Rheumatol. 2010;**37**(4):851-9.

82. González-Viejo MA, Avellanet M, Hernández-Morcuende MI. A comparative study of fibromyalgia treatment: ultrasonography and physiotherapy versus sertraline treatment. Ann Readapt Med Phys. 2005;**48**(8):610‐5.

83. Moldofsky H, Inhaber NH, Guinta DR, et al. Effects of sodium oxybate on sleep physiology and sleep/wake-related symptoms in patients with fibromyalgia syndrome: a double-blind, randomized, placebo-controlled study. J Rheumatol. 2010;**37**(10):2156‐66.

84. Spaeth M, Bennett RM, Benson BA, et al. Sodium oxybate therapy provides multidimensional improvement in fibromyalgia: results of an international phase 3 trial. Ann Rheum Dis. 2012;**71**(6):935‐42.

85. Russell JI, Holman AJ, Swick TJ, et al. Sodium oxybate reduces pain, fatigue, and sleep disturbance and improves functionality in fibromyalgia: results from a 14-week, randomized, double-blind, placebo-controlled study. Pain. 2011;**152**(5):1007‐17.

86. Vitton O, Gendreau M, Gendreau J, et al. A double-blind placebo-controlled trial of milnacipran in the treatment of fibromyalgia. Hum Psychopharmacol. 2004;**19 Suppl 1**:S27‐35.

87. Yeephu S, Suthisisang C, Suttiruksa S, et al. Efficacy and safety of mirtazapine in fibromyalgia syndrome patients: a randomized placebo-controlled pilot study. Ann Pharmacother. 2013;**47**(7‐8):921‐32.

88. Arnold LM, Blauwet MB, Tracy K, et al. Efficacy and safety of asp0819 in patients with fibromyalgia: results of a proof-of-concept, randomized, double-blind, placebo-controlled trial. J Pain Res. 2020;**13**:3355-69.

89. Pauer L, Winkelmann A, Arsenault P, et al. An international, randomized, double-blind, placebo-controlled, phase III trial of pregabalin monotherapy in treatment of patients with fibromyalgia. J Rheumatol. 2011;**38**(12):2643‐52.

90. Mameli S, Pisanu GM, Sardo S, et al. Oxytocin nasal spray in fibromyalgic patients. Rheumatol Int. 2014;**34**(8):1047‐52.
