# Supplementary material for: Effects of Pharmacologic and Nonpharmacologic Interventions for the Management of Sleep Problems in People With Fibromyalgia: Systematic Review and Network Meta‐Analysis of Randomized Controlled Trials
Source: Arthritis Care Res (Hoboken). 2025 Mar 26;77(9):1095–105. doi: 10.1002/acr.25505 (PMC12371313; doi:10.1002/acr.25505)
Supplement: Supplementary file 4 — Appendix 3: Network‐meta‐analysis results for sleep quality outcome [file ACR-77-1095-s005.docx]

**Appendix 3 Network-meta-analysis results for sleep quality outcome**

**Table 1. Interventions and the number of participants**

| **Intervention** | **n** |
| --- | --- |
| Placebo/Sham | 2087 |
| Education + Flexibility exercise LD | 33 |
| Mind-body exercise LD | 465 |
| Aerobic exercise LD | 107 |
| Education | 182 |
| Usual care | 924 |
| Aerobic exercise AQ | 59 |
| Nutrition | 42 |
| Balneotherapy | 127 |
| PT/BT generic | 352 |
| Manual therapy | 59 |
| Relaxation | 116 |
| Electrotherapy | 49 |
| Flexibility exercise LD | 79 |
| PT/BT sleep | 94 |
| Mind-body exercise AQ | 18 |
| Mixed exercise AQ | 97 |
| Weight loss | 41 |
| Neuromodulation | 158 |
| Non-mainstream practice | 75 |
| Dental splint | 29 |
| HBOT | 9 |
| Aerobic exercise LD + Flexibility exercise LD | 32 |
| Multidisciplinary | 81 |
| Flexibility exercise LD + Manual Therapy | 17 |
| Balneotherapy + Mixed exercise AQ | 36 |
| Strengthening exercise LD | 56 |
| Tricyclics | 43 |
| Antipsychotics | 53 |
| Endogenous hormones | 14 |
| Antioxidant | 12 |
| SRI | 668 |
| Gabapentinoid | 1474 |
| Analgesic | 90 |
| CNS depressants | 469 |

**Abbreviation:** FIQ Fibromyalgia Impact Questionnaire; AQ, aquatic; LD, land-based; PT/BT, psychological or behavioural therapy; CNS Central nervous system; HBOT Hyperbaric oxygen therapy.

**Table 2. Direct evidence compared to placebo/sham**

| **Intervention** | **Number of trials** | | **SMD** | **95% CI** |
| --- | --- | --- | --- | --- |
| Education + Flexibility exercise LD | | - |  |  |
| Mind-body exercise LD | | 2 | -1.01 | (-1.76, -0.26) |
| Aerobic exercise LD | | - |  |  |
| Education | | - |  |  |
| Usual care | | 4 | 0.20 | (-0.04, 0.44) |
| Aerobic exercise AQ | | - |  |  |
| Nutrition | | 1 | 0.26 | (-0.32, 0.83) |
| Balneotherapy | | - |  |  |
| PT/BT generic | | 1 | -0.01 | (-0.47, 0.45) |
| Manual therapy | | 1 | -0.31 | (-1.13, 0.51) |
| Relaxation | | 1 | -0.39 | (-0.81, 0.03) |
| Electrotherapy | | 1 | -0.98 | (-1.67, -0.29) |
| Flexibility exercise LD | | - |  |  |
| PT/BT sleep | | - |  |  |
| Mind-body exercise AQ | | 1 | 4.25 | (3.00, 5.49) |
| Mixed exercise AQ | | - |  |  |
| Weight loss | | - |  |  |
| Neuromodulation | | 3 | -0.32 | (-0.63, -0.02) |
| Non-mainstream practice | | 2 | -0.98 | (-1.38, -0.58) |
| Dental splint | | - |  |  |
| HBOT | | - |  |  |
| Aerobic exercise LD + Flexibility exercise LD | | - |  |  |
| Multidisciplinary | | - |  |  |
| Flexibility exercise LD + Manual Therapy | | - |  |  |
| Balneotherapy + Mixed exercise AQ | | - |  |  |
| Strengthening exercise LD | | - |  |  |
| Tricyclics | | - |  |  |
| Antipsychotics | | 1 | -1.29 | (-1.95, -0.63) |
| Endogenous hormones | | 1 | 0.25 | (-0.50, 0.99) |
| Antioxidant | | 1 | -0.29 | (-1.13, 0.55) |
| SRI | | 4 | -0.01 | (-0.12, 0.10) |
| Gabapentinoid | | 5 | -0.28 | (-0.37, -0.19) |
| Analgesic | | 1 | -0.25 | (-0.54, 0.04) |
| CNS depressants | | 3 | -0.44 | (-0.59, -0.28) |

**Note:** Negative values indicate a better outcome while positive values indicate a worse outcome.

**Abbreviations:** AQ, aquatic; CI Confidence interval; CNS, central nervous system; CI Confidence interval. LD, land-based; NMA Network meta-analysis; PT/BT, psychological or behavioural therapy; SMD standardised mean difference; SRI, serotonin reuptake inhibitors; HBOT Hyperbaric oxygen therapy.

**Table 3. Results for direct and NMA compared to Education + Flexibility exercise LD**

|  | **Direct evidence** | | | **NMA** | |
| --- | --- | --- | --- | --- | --- |
| **Intervention** | **Number of trials** | **SMD** | **95% CI** | **SMD** | **95% Crl** |
| Mind-body exercise LD | 1 | -0.81 | (-1.32, -0.31) | -0.82 | (-3.08, 1.42) |
| Aerobic exercise LD | - |  |  | -0.76 | (-3.97, 2.39) |
| Education | - |  |  | -0.53 | (-3.03, 1.96) |
| Usual care | - |  |  | -0.78 | (-3.24, 1.61) |
| Aerobic exercise AQ | - |  |  | -3.25 | (-6.17, -0.34) |
| Nutrition | - |  |  | -0.77 | (-3.66, 2.06) |
| Balneotherapy | - |  |  | -1.21 | (-4.19, 1.73) |
| PT/BT generic | - |  |  | -1.05 | (-3.61, 1.43) |
| Manual therapy | - |  |  | -1.13 | (-4.05, 1.78) |
| Relaxation | - |  |  | -1.23 | (-4.31, 1.87) |
| Electrotherapy | - |  |  | -1.59 | (-5.01, 1.83) |
| Flexibility exercise LD | - |  |  | -0.12 | (-3.10, 2.84) |
| PT/BT sleep | - |  |  | -1.51 | (-4.17, 1.12) |
| Mind-body exercise AQ | - |  |  | 3.65 | (0.10, 7.16) |
| Mixed exercise AQ | - |  |  | -0.80 | (-3.65, 1.97) |
| Weight loss | - |  |  | -1.76 | (-5.12, 1.48) |
| Neuromodulation | - |  |  | -0.87 | (-3.71, 1.96) |
| Non-mainstream practice | - |  |  | -1.78 | (-4.65, 1.06) |
| Dental splint | - |  |  | -2.23 | (-6.34, 1.88) |
| HBOT | - |  |  | -5.13 | (-8.83, -1.40) |
| Aerobic exercise LD + Flexibility exercise LD | - |  |  | -5.31 | (-9.29, -1.43) |
| Multidisciplinary | - |  |  | 1.19 | (-2.13, 4.49) |
| Flexibility exercise LD + Manual Therapy | - |  |  | 0.18 | (-3.61, 3.92) |
| Balneotherapy + Mixed exercise AQ | - |  |  | -0.24 | (-3.67, 3.09) |
| Tricyclics | - |  |  | -1.88 | (-5.93, 2.18) |
| Antipsychotics | - |  |  | -1.90 | (-5.28, 1.46) |
| Endogenous hormones | - |  |  | -0.37 | (-3.81, 3.00) |
| Antioxidant | - |  |  | -0.90 | (-4.31, 2.52) |
| SRI | - |  |  | -0.63 | (-3.40, 2.11) |
| Gabapentinoid | - |  |  | -1.03 | (-3.77, 1.66) |
| Analgesic | - |  |  | -0.86 | (-4.22, 2.44) |
| CNS depressants | - |  |  | -0.80 | (-3.65, 2.04) |
| Strengthening exercise LD | - |  |  | -1.58 | (-5.35, 2.28) |

NMA Network meta-analysis; SMD Standardised mean difference; CI Confidence interval; Crl Credible interval. Note: Negative values indicate a better outcome while higher values indicate a worse outcome.

**Table 4. Results for direct and NMA compared to Mind-body exercise LD**

|  | **Direct evidence** | | | **NMA** | |
| --- | --- | --- | --- | --- | --- |
| **Intervention** | **Number of trials** | **SMD** | **95% CI** | **SMD** | **95% Crl** |
| Aerobic exercise LD | 1 | 0.05 | (-0.23, 0.32) | 0.05 | (-2.16, 2.24) |
| Education | 3 | 0.47 | (0.21, 0.73) | 0.28 | (-0.82, 1.39) |
| Usual care | 4 | 0.28 | (-0.02, 0.57) | 0.04 | (-0.88, 0.90) |
| Aerobic exercise AQ | 1 | -0.97 | (-1.61, -0.32) | -2.43 | (-4.34, -0.58) |
| Nutrition | - |  |  | 0.04 | (-1.71, 1.78) |
| Balneotherapy | - |  |  | -0.40 | (-2.29, 1.49) |
| PT/BT generic | - |  |  | -0.24 | (-1.37, 0.86) |
| Manual therapy | - |  |  | -0.32 | (-2.15, 1.50) |
| Relaxation | - |  |  | -0.43 | (-2.51, 1.68) |
| Electrotherapy | - |  |  | -0.78 | (-3.33, 1.76) |
| Flexibility exercise LD | - |  |  | 0.69 | (-1.25, 2.67) |
| PT/BT sleep | - |  |  | -0.69 | (-2.08, 0.68) |
| Mind-body exercise AQ | - |  |  | 4.45 | (1.73, 7.13) |
| Mixed exercise AQ | - |  |  | 0.00 | (-1.67, 1.62) |
| Weight loss | - |  |  | -0.95 | (-3.37, 1.45) |
| Neuromodulation | - |  |  | -0.05 | (-1.77, 1.63) |
| Non-mainstream practice | - |  |  | -0.95 | (-2.73, 0.76) |
| Dental splint | - |  |  | -1.41 | (-4.85, 2.01) |
| HBOT | - |  |  | -4.32 | (-7.26, -1.37) |
| Aerobic exercise LD + Flexibility exercise LD | - |  |  | -4.51 | (-7.77, -1.29) |
| Multidisciplinary | - |  |  | 2.00 | (-0.43, 4.38) |
| Flexibility exercise LD + Manual Therapy | - |  |  | 0.98 | (-2.05, 3.97) |
| Balneotherapy + Mixed exercise AQ | - |  |  | 0.58 | (-1.95, 3.05) |
| Tricyclics | - |  |  | -1.05 | (-4.46, 2.31) |
| Antipsychotics | - |  |  | -1.08 | (-3.62, 1.41) |
| Endogenous hormones | - |  |  | 0.44 | (-2.11, 2.97) |
| Antioxidant | - |  |  | -0.09 | (-2.65, 2.48) |
| SRI | - |  |  | 0.18 | (-1.37, 1.72) |
| Gabapentinoid | - |  |  | -0.22 | (-1.71, 1.22) |
| Analgesic | - |  |  | -0.04 | (-2.53, 2.39) |
| CNS depressants | - |  |  | 0.01 | (-1.68, 1.72) |
| Strengthening exercise LD | - |  |  | -0.76 | (-3.81, 2.32) |

NMA Network meta-analysis; SMD Standardised mean difference; CI Confidence interval; Crl Credible interval. Negative values indicate a better outcome while higher values indicate a worse outcome.

**Table 5. Results for direct and NMA compared to Aerobic exercise LD**

|  | **Direct evidence** | | | **NMA** | |
| --- | --- | --- | --- | --- | --- |
| **Intervention** | **Number of trials** | **SMD** | **95% CI** | **SMD** | **95% Crl** |
| Education | - |  |  | 0.23 | (-2.22, 2.70) |
| Usual care | - |  |  | -0.02 | (-2.40, 2.37) |
| Aerobic exercise AQ | - |  |  | -2.49 | (-5.39, 0.40) |
| Nutrition | - |  |  | -0.01 | (-2.84, 2.81) |
| Balneotherapy | - |  |  | -0.45 | (-3.37, 2.47) |
| PT/BT generic | - |  |  | -0.29 | (-2.77, 2.18) |
| Manual therapy | - |  |  | -0.38 | (-3.22, 2.50) |
| Relaxation | - |  |  | -0.47 | (-3.49, 2.60) |
| Electrotherapy | - |  |  | -0.84 | (-4.20, 2.53) |
| Flexibility exercise LD | - |  |  | 0.64 | (-2.28, 3.60) |
| PT/BT sleep | - |  |  | -0.75 | (-3.33, 1.84) |
| Mind-body exercise AQ | - |  |  | 4.41 | (0.93, 7.94) |
| Mixed exercise AQ | - |  |  | -0.04 | (-2.80, 2.68) |
| Weight loss | - |  |  | -1.01 | (-4.26, 2.27) |
| Neuromodulation | - |  |  | -0.11 | (-2.88, 2.70) |
| Non-mainstream practice | - |  |  | -1.01 | (-3.82, 1.79) |
| Dental splint | - |  |  | -1.47 | (-5.52, 2.63) |
| HBOT | - |  |  | -4.36 | (-8.04, -0.70) |
| Aerobic exercise LD + Flexibility exercise LD | 1 | -4.56 | (-5.51, -3.61) | -4.56 | (-6.94, -2.17) |
| Multidisciplinary | - |  |  | 1.95 | (-1.28, 5.24) |
| Flexibility exercise LD + Manual Therapy | - |  |  | 0.94 | (-2.76, 4.63) |
| Balneotherapy + Mixed exercise AQ | - |  |  | 0.53 | (-2.81, 3.84) |
| Tricyclics | - |  |  | -1.11 | (-5.14, 2.96) |
| Antipsychotics | - |  |  | -1.14 | (-4.49, 2.23) |
| Endogenous hormones | - |  |  | 0.39 | (-2.98, 3.73) |
| Antioxidant | - |  |  | -0.13 | (-3.53, 3.25) |
| SRI | - |  |  | 0.14 | (-2.57, 2.83) |
| Gabapentinoid | - |  |  | -0.29 | (-2.92, 2.40) |
| Analgesic | - |  |  | -0.10 | (-3.40, 3.22) |
| CNS depressants | - |  |  | -0.04 | (-2.80, 2.78) |
| Strengthening exercise LD | - |  |  | -0.82 | (-4.57, 2.99) |

NMA Network meta-analysis; SMD Standardised mean difference; CI Confidence interval; Crl Credible interval. Negative values indicate a better outcome while higher values indicate a worse outcome.

**Table 6. Results for direct and NMA compared to Education**

|  | **Direct evidence** | | | **NMA** | |
| --- | --- | --- | --- | --- | --- |
| **Intervention** | **Number of trials** | **SMD** | **95% CI** | **SMD** | **95% Crl** |
| Usual care | - |  |  | -0.25 | (-1.45, 0.95) |
| Aerobic exercise AQ | - |  |  | -2.72 | (-4.88, -0.59) |
| Nutrition | - |  |  | -0.24 | (-2.17, 1.69) |
| Balneotherapy | - |  |  | -0.68 | (-2.65, 1.29) |
| PT/BT generic | - |  |  | -0.52 | (-1.84, 0.79) |
| Manual therapy | - |  |  | -0.60 | (-2.60, 1.43) |
| Relaxation | - |  |  | -0.71 | (-2.96, 1.57) |
| Electrotherapy | - |  |  | -1.06 | (-3.74, 1.61) |
| Flexibility exercise LD | - |  |  | 0.41 | (-1.73, 2.59) |
| PT/BT sleep | 2 | -0.81 | (-1.23, -0.39) | -0.97 | (-2.26, 0.29) |
| Mind-body exercise AQ | - |  |  | 4.17 | (1.31, 7.02) |
| Mixed exercise AQ | 1 | 0.12 | (-0.46, 0.71) | -0.28 | (-1.89, 1.31) |
| Weight loss | - |  |  | -1.24 | (-3.79, 1.32) |
| Neuromodulation | - |  |  | -0.33 | (-2.25, 1.58) |
| Non-mainstream practice | - |  |  | -1.23 | (-3.21, 0.69) |
| Dental splint | - |  |  | -1.70 | (-5.25, 1.85) |
| HBOT | - |  |  | -4.60 | (-7.63, -1.55) |
| Aerobic exercise LD + Flexibility exercise LD | - |  |  | -4.79 | (-8.22, -1.39) |
| Multidisciplinary | - |  |  | 1.71 | (-0.80, 4.25) |
| Flexibility exercise LD + Manual Therapy | - |  |  | 0.70 | (-2.45, 3.83) |
| Balneotherapy + Mixed exercise AQ | - |  |  | 0.29 | (-2.26, 2.76) |
| Tricyclics | - |  |  | -1.34 | (-4.81, 2.15) |
| Antipsychotics | - |  |  | -1.37 | (-4.05, 1.27) |
| Endogenous hormones | - |  |  | 0.15 | (-2.53, 2.82) |
| Antioxidant | - |  |  | -0.37 | (-3.09, 2.35) |
| SRI | - |  |  | -0.09 | (-1.89, 1.70) |
| Gabapentinoid | - |  |  | -0.51 | (-2.23, 1.21) |
| Analgesic | - |  |  | -0.32 | (-2.96, 2.27) |
| CNS depressants | - |  |  | -0.27 | (-2.19, 1.65) |
| Strengthening exercise LD | - |  |  | -1.04 | (-4.17, 2.17) |

NMA Network meta-analysis; SMD Standardised mean difference; CI Confidence interval; Crl Credible interval. Negative values indicate a better outcome while higher values indicate a worse outcome.

**Table 7. Results for direct and NMA compared to Usual care**

|  | **Direct evidence** | | | **NMA** | |
| --- | --- | --- | --- | --- | --- |
| **Intervention** | **Number of trials** | **SMD** | **95% CI** | **SMD** | **95% Crl** |
| Aerobic exercise AQ | - |  |  | -2.47 | (-4.43, -0.52) |
| Nutrition | 1 | -0.30 | (-1.39, 0.80) | 0.01 | (-1.52, 1.56) |
| Balneotherapy | 1 | 0.04 | (-0.25, 0.32) | -0.44 | (-2.17, 1.32) |
| PT/BT generic | 7 | -0.20 | (-0.37, -0.03) | -0.27 | (-1.03, 0.49) |
| Manual therapy | 1 | -0.54 | (-0.96, -0.11) | -0.35 | (-2.00, 1.32) |
| Relaxation | - |  |  | -0.46 | (-2.39, 1.53) |
| Electrotherapy | - |  |  | -0.82 | (-3.27, 1.69) |
| Flexibility exercise LD | 1 | -0.79 | (-1.42, -0.16) | 0.66 | (-1.22, 2.57) |
| PT/BT sleep | 1 | -0.57 | (-1.18, 0.03) | -0.72 | (-2.00, 0.56) |
| Mind-body exercise AQ | - |  |  | 4.42 | (1.80, 7.07) |
| Mixed exercise AQ | 1 | -0.92 | (-1.47, -0.37) | -0.03 | (-1.53, 1.48) |
| Weight loss | 1 | -0.98 | (-1.44, -0.53) | -0.99 | (-3.20, 1.27) |
| Neuromodulation | - |  |  | -0.09 | (-1.66, 1.51) |
| Non-mainstream practice | - |  |  | -0.99 | (-2.59, 0.63) |
| Dental splint | - |  |  | -1.44 | (-4.79, 1.95) |
| HBOT | 1 | -4.35 | (-6.28, -2.43) | -4.35 | (-7.15, -1.54) |
| Aerobic exercise LD + Flexibility exercise LD | - |  |  | -4.53 | (-7.91, -1.17) |
| Multidisciplinary | 1 | 1.97 | (1.58, 2.35) | 1.96 | (-0.26, 4.20) |
| Flexibility exercise LD + Manual Therapy | - |  |  | 0.94 | (-2.00, 3.89) |
| Balneotherapy + Mixed exercise AQ | - |  |  | 0.54 | (-1.87, 2.92) |
| Tricyclics | - |  |  | -1.09 | (-4.38, 2.23) |
| Antipsychotics | - |  |  | -1.12 | (-3.56, 1.32) |
| Endogenous hormones | - |  |  | 0.40 | (-2.04, 2.87) |
| Antioxidant | - |  |  | -0.12 | (-2.60, 2.37) |
| SRI | - |  |  | 0.15 | (-1.26, 1.57) |
| Gabapentinoid | - |  |  | -0.26 | (-1.59, 1.08) |
| Analgesic | - |  |  | -0.07 | (-2.46, 2.30) |
| CNS depressants | - |  |  | -0.02 | (-1.59, 1.59) |
| Strengthening exercise LD | - |  |  | -0.79 | (-3.73, 2.21) |

NMA Network meta-analysis; SMD Standardised mean difference; CI Confidence interval; Crl Credible interval. Negative values indicate a better outcome while higher values indicate a worse outcome.

**Table 8. Results for direct and NMA compared to Aerobic exercise AQ**

|  | **Direct evidence** | | | **NMA** | |
| --- | --- | --- | --- | --- | --- |
| **Intervention** | **Number of trials** | **SMD** | **95% CI** | **SMD** | **95% Crl** |
| Nutrition | - |  |  | 2.49 | (-0.00, 4.96) |
| Balneotherapy | - |  |  | 2.03 | (-0.56, 4.63) |
| PT/BT generic | - |  |  | 2.20 | (0.14, 4.28) |
| Manual therapy | - |  |  | 2.12 | (-0.42, 4.65) |
| Relaxation | - |  |  | 2.01 | (-0.69, 4.75) |
| Electrotherapy | - |  |  | 1.66 | (-1.42, 4.77) |
| Flexibility exercise LD | 1 | 4.71 | (3.82, 5.60) | 3.13 | (1.22, 5.08) |
| PT/BT sleep | - |  |  | 1.74 | (-0.52, 3.99) |
| Mind-body exercise AQ | - |  |  | 6.89 | (3.67, 10.15) |
| Mixed exercise AQ | - |  |  | 2.44 | (0.05, 4.86) |
| Weight loss | - |  |  | 1.48 | (-1.50, 4.47) |
| Neuromodulation | - |  |  | 2.37 | (-0.05, 4.87) |
| Non-mainstream practice | - |  |  | 1.48 | (-1.02, 3.97) |
| Dental splint | - |  |  | 1.02 | (-2.87, 4.88) |
| HBOT | - |  |  | -1.88 | (-5.23, 1.56) |
| Aerobic exercise LD + Flexibility exercise LD | - |  |  | -2.07 | (-5.83, 1.66) |
| Multidisciplinary | - |  |  | 4.44 | (1.45, 7.38) |
| Flexibility exercise LD + Manual Therapy | - |  |  | 3.42 | (0.44, 6.43) |
| Balneotherapy + Mixed exercise AQ | - |  |  | 3.02 | (-0.03, 6.07) |
| Tricyclics | - |  |  | 1.38 | (-2.42, 5.21) |
| Antipsychotics | - |  |  | 1.35 | (-1.70, 4.41) |
| Endogenous hormones | - |  |  | 2.87 | (-0.22, 6.01) |
| Antioxidant | - |  |  | 2.34 | (-0.77, 5.47) |
| SRI | - |  |  | 2.62 | (0.28, 5.00) |
| Gabapentinoid | - |  |  | 2.21 | (-0.07, 4.53) |
| Analgesic | - |  |  | 2.39 | (-0.64, 5.44) |
| CNS depressants | - |  |  | 2.45 | (-0.00, 4.92) |
| Strengthening exercise LD | - |  |  | 1.68 | (-1.85, 5.21) |

NMA Network meta-analysis; SMD Standardised mean difference; CI Confidence interval; Crl Credible interval. Negative values indicate a better outcome while higher values indicate a worse outcome.

**Table 9. Results for direct and NMA compared to Nutrition**

|  | **Direct evidence** | | | **NMA** | |
| --- | --- | --- | --- | --- | --- |
| **Intervention** | **Number of trials** | **SMD** | **95% CI** | **SMD** | **95% Crl** |
| Balneotherapy | - |  |  | -0.45 | (-2.77, 1.88) |
| PT/BT generic | - |  |  | -0.28 | (-2.00, 1.42) |
| Manual therapy | - |  |  | -0.36 | (-2.55, 1.86) |
| Relaxation | - |  |  | -0.47 | (-2.89, 2.01) |
| Electrotherapy | - |  |  | -0.83 | (-3.63, 2.01) |
| Flexibility exercise LD | - |  |  | 0.65 | (-1.77, 3.10) |
| PT/BT sleep | - |  |  | -0.74 | (-2.72, 1.27) |
| Mind-body exercise AQ | - |  |  | 4.42 | (1.43, 7.40) |
| Mixed exercise AQ | - |  |  | -0.03 | (-2.19, 2.12) |
| Weight loss | - |  |  | -0.99 | (-3.70, 1.72) |
| Neuromodulation | - |  |  | -0.09 | (-2.23, 2.02) |
| Non-mainstream practice | - |  |  | -1.00 | (-3.15, 1.16) |
| Dental splint | - |  |  | -1.46 | (-5.08, 2.21) |
| HBOT | - |  |  | -4.35 | (-7.54, -1.14) |
| Aerobic exercise LD + Flexibility exercise LD | - |  |  | -4.54 | (-8.22, -0.83) |
| Multidisciplinary | - |  |  | 1.95 | (-0.73, 4.67) |
| Flexibility exercise LD + Manual Therapy | - |  |  | 0.94 | (-2.38, 4.27) |
| Balneotherapy + Mixed exercise AQ | - |  |  | 0.53 | (-2.31, 3.38) |
| Tricyclics | - |  |  | -1.09 | (-4.69, 2.48) |
| Antipsychotics | - |  |  | -1.12 | (-3.94, 1.67) |
| Endogenous hormones | - |  |  | 0.40 | (-2.45, 3.20) |
| Antioxidant | - |  |  | -0.14 | (-2.98, 2.72) |
| SRI | - |  |  | 0.14 | (-1.85, 2.14) |
| Gabapentinoid | - |  |  | -0.27 | (-2.19, 1.68) |
| Analgesic | - |  |  | -0.09 | (-2.82, 2.68) |
| CNS depressants | - |  |  | -0.03 | (-2.14, 2.08) |
| Strengthening exercise LD | - |  |  | -0.81 | (-4.09, 2.55) |

NMA Network meta-analysis; SMD Standardised mean difference; CI Confidence interval; Crl Credible interval. Negative values indicate a better outcome while higher values indicate a worse outcome.

**Table 10. Results for direct and NMA compared to Balneotherapy**

|  | **Direct evidence** | | | **NMA** | |
| --- | --- | --- | --- | --- | --- |
| **Intervention** | **Number of trials** | **SMD** | **95% CI** | **SMD** | **95% Crl** |
| PT/BT generic | - |  |  | 0.16 | (-1.72, 2.04) |
| Manual therapy | - |  |  | 0.08 | (-2.27, 2.48) |
| Relaxation | - |  |  | -0.03 | (-2.62, 2.61) |
| Electrotherapy | - |  |  | -0.38 | (-3.40, 2.62) |
| Flexibility exercise LD | - |  |  | 1.09 | (-1.43, 3.69) |
| PT/BT sleep | - |  |  | -0.29 | (-2.38, 1.80) |
| Mind-body exercise AQ | - |  |  | 4.85 | (1.71, 8.03) |
| Mixed exercise AQ | 1 | 0.83 | (0.25, 1.41) | 0.40 | (-1.35, 2.15) |
| Weight loss | - |  |  | -0.55 | (-3.39, 2.29) |
| Neuromodulation | - |  |  | 0.35 | (-1.98, 2.70) |
| Non-mainstream practice | - |  |  | -0.55 | (-2.94, 1.79) |
| Dental splint | - |  |  | -1.01 | (-4.80, 2.78) |
| HBOT | - |  |  | -3.92 | (-7.19, -0.63) |
| Aerobic exercise LD + Flexibility exercise LD | - |  |  | -4.11 | (-7.85, -0.35) |
| Multidisciplinary | - |  |  | 2.40 | (-0.45, 5.27) |
| Flexibility exercise LD + Manual Therapy | - |  |  | 1.38 | (-2.02, 4.82) |
| Balneotherapy + Mixed exercise AQ | 1 | 1.20 | (0.59, 1.82) | 0.98 | (-1.16, 3.09) |
| Tricyclics | - |  |  | -0.65 | (-4.35, 3.07) |
| Antipsychotics | - |  |  | -0.68 | (-3.66, 2.29) |
| Endogenous hormones | - |  |  | 0.84 | (-2.18, 3.85) |
| Antioxidant | - |  |  | 0.31 | (-2.70, 3.34) |
| SRI | - |  |  | 0.59 | (-1.65, 2.83) |
| Gabapentinoid | - |  |  | 0.19 | (-2.02, 2.35) |
| Analgesic | - |  |  | 0.36 | (-2.58, 3.29) |
| CNS depressants | - |  |  | 0.41 | (-1.92, 2.75) |
| Strengthening exercise LD | - |  |  | -0.36 | (-3.73, 3.08) |

NMA Network meta-analysis; SMD Standardised mean difference; CI Confidence interval; Crl Credible interval. Negative values indicate a better outcome while higher values indicate a worse outcome.

**Table 11. Results for direct and NMA compared to PT/BT generic**

|  | **Direct evidence** | | | **NMA** | |
| --- | --- | --- | --- | --- | --- |
| **Intervention** | **Number of trials** | **SMD** | **95% CI** | **SMD** | **95% Crl** |
| Manual therapy | - |  |  | -0.08 | (-1.88, 1.75) |
| Relaxation | - |  |  | -0.18 | (-2.24, 1.92) |
| Electrotherapy | - |  |  | -0.54 | (-3.08, 2.05) |
| Flexibility exercise LD | - |  |  | 0.93 | (-1.07, 2.99) |
| PT/BT sleep | 1 | -0.93 | (-1.67, -0.20) | -0.45 | (-1.74, 0.84) |
| Mind-body exercise AQ | - |  |  | 4.70 | (1.99, 7.43) |
| Mixed exercise AQ | - |  |  | 0.24 | (-1.41, 1.89) |
| Weight loss | - |  |  | -0.72 | (-3.03, 1.67) |
| Neuromodulation | - |  |  | 0.18 | (-1.52, 1.92) |
| Non-mainstream practice | - |  |  | -0.71 | (-2.45, 1.02) |
| Dental splint | - |  |  | -1.18 | (-4.61, 2.26) |
| HBOT | - |  |  | -4.07 | (-6.96, -1.17) |
| Aerobic exercise LD + Flexibility exercise LD | - |  |  | -4.26 | (-7.70, -0.83) |
| Multidisciplinary | - |  |  | 2.23 | (-0.13, 4.59) |
| Flexibility exercise LD + Manual Therapy | - |  |  | 1.21 | (-1.84, 4.29) |
| Balneotherapy + Mixed exercise AQ | - |  |  | 0.82 | (-1.69, 3.26) |
| Tricyclics | - |  |  | -0.82 | (-4.17, 2.58) |
| Antipsychotics | - |  |  | -0.85 | (-3.34, 1.68) |
| Endogenous hormones | - |  |  | 0.68 | (-1.87, 3.21) |
| Antioxidant | - |  |  | 0.15 | (-2.42, 2.72) |
| SRI | - |  |  | 0.43 | (-1.15, 2.01) |
| Gabapentinoid | - |  |  | 0.02 | (-1.47, 1.51) |
| Analgesic | - |  |  | 0.20 | (-2.27, 2.66) |
| CNS depressants | - |  |  | 0.25 | (-1.44, 1.98) |
| Strengthening exercise LD | - |  |  | -0.52 | (-3.55, 2.56) |

NMA Network meta-analysis; SMD Standardised mean difference; CI Confidence interval; Crl Credible interval. Negative values indicate a better outcome while higher values indicate a worse outcome.

**Table 12. Results for direct and NMA compared to Manual therapy**

|  | **Direct evidence** | | | **NMA** | |
| --- | --- | --- | --- | --- | --- |
| **Intervention** | **Number of trials** | **SMD** | **95% CI** | **SMD** | **95% Crl** |
| Relaxation | - |  |  | -0.11 | (-2.59, 2.41) |
| Electrotherapy | - |  |  | -0.46 | (-3.30, 2.39) |
| Flexibility exercise LD | - |  |  | 1.02 | (-1.50, 3.51) |
| PT/BT sleep | - |  |  | -0.37 | (-2.46, 1.70) |
| Mind-body exercise AQ | - |  |  | 4.78 | (1.79, 7.79) |
| Mixed exercise AQ | - |  |  | 0.33 | (-1.91, 2.55) |
| Weight loss | - |  |  | -0.63 | (-3.42, 2.13) |
| Neuromodulation | - |  |  | 0.26 | (-1.86, 2.40) |
| Non-mainstream practice | - |  |  | -0.65 | (-2.83, 1.56) |
| Dental splint | - |  |  | -1.09 | (-4.75, 2.53) |
| HBOT | - |  |  | -3.99 | (-7.23, -0.75) |
| Aerobic exercise LD + Flexibility exercise LD | - |  |  | -4.18 | (-7.87, -0.49) |
| Multidisciplinary | - |  |  | 2.33 | (-0.45, 5.06) |
| Flexibility exercise LD + Manual Therapy | - |  |  | 1.29 | (-2.10, 4.67) |
| Balneotherapy + Mixed exercise AQ | - |  |  | 0.89 | (-2.02, 3.77) |
| Tricyclics | - |  |  | -0.73 | (-4.34, 2.88) |
| Antipsychotics | - |  |  | -0.77 | (-3.61, 2.04) |
| Endogenous hormones | - |  |  | 0.75 | (-2.07, 3.58) |
| Antioxidant | - |  |  | 0.23 | (-2.66, 3.10) |
| SRI | - |  |  | 0.50 | (-1.50, 2.49) |
| Gabapentinoid | - |  |  | 0.09 | (-1.84, 2.02) |
| Analgesic | - |  |  | 0.27 | (-2.51, 3.03) |
| CNS depressants | - |  |  | 0.33 | (-1.80, 2.47) |
| Strengthening exercise LD | - |  |  | -0.44 | (-3.79, 2.92) |

NMA Network meta-analysis; SMD Standardised mean difference; CI Confidence interval; Crl Credible interval. Negative values indicate a better outcome while higher values indicate a worse outcome.

**Table 13. Results for direct and NMA compared to Relaxation**

|  | **Direct evidence** | | | **NMA** | |
| --- | --- | --- | --- | --- | --- |
| **Intervention** | **Number of trials** | **SMD** | **95% CI** | **SMD** | **95% Crl** |
| Electrotherapy | - |  |  | -0.35 | (-3.42, 2.63) |
| Flexibility exercise LD | - |  |  | 1.12 | (-1.59, 3.82) |
| PT/BT sleep | - |  |  | -0.26 | (-2.60, 2.04) |
| Mind-body exercise AQ | - |  |  | 4.89 | (1.72, 8.01) |
| Mixed exercise AQ | - |  |  | 0.43 | (-2.06, 2.86) |
| Weight loss | - |  |  | -0.53 | (-3.50, 2.46) |
| Neuromodulation | - |  |  | 0.36 | (-2.00, 2.73) |
| Non-mainstream practice | - |  |  | -0.54 | (-2.98, 1.88) |
| Dental splint | - |  |  | -0.98 | (-4.78, 2.79) |
| HBOT | - |  |  | -3.89 | (-7.32, -0.48) |
| Aerobic exercise LD + Flexibility exercise LD | - |  |  | -4.07 | (-7.96, -0.24) |
| Multidisciplinary | - |  |  | 2.42 | (-0.55, 5.38) |
| Flexibility exercise LD + Manual Therapy | - |  |  | 1.40 | (-2.11, 4.95) |
| Balneotherapy + Mixed exercise AQ | - |  |  | 0.99 | (-2.08, 4.05) |
| Tricyclics | - |  |  | -0.63 | (-4.39, 3.13) |
| Antipsychotics | - |  |  | -0.66 | (-3.66, 2.30) |
| Endogenous hormones | - |  |  | 0.86 | (-2.13, 3.86) |
| Antioxidant | - |  |  | 0.34 | (-2.74, 3.35) |
| SRI | - |  |  | 0.61 | (-1.66, 2.85) |
| Gabapentinoid | - |  |  | 0.20 | (-1.99, 2.39) |
| Analgesic | - |  |  | 0.37 | (-2.59, 3.33) |
| CNS depressants | - |  |  | 0.44 | (-1.92, 2.79) |
| Strengthening exercise LD | 1 | -0.34 | (-0.73, 0.05) | -0.33 | (-2.54, 1.85) |

NMA Network meta-analysis; SMD Standardised mean difference; CI Confidence interval; Crl Credible interval. Negative values indicate a better outcome while higher values indicate a worse outcome.

**Table 14. Results for direct and NMA compared to Electrotherapy**

|  | **Direct evidence** | | | **NMA** | |
| --- | --- | --- | --- | --- | --- |
| **Intervention** | **Number of trials** | **SMD** | **95% CI** | **SMD** | **95% Crl** |
| Flexibility exercise LD | - |  |  | 1.48 | (-1.65, 4.58) |
| PT/BT sleep | - |  |  | 0.10 | (-2.66, 2.81) |
| Mind-body exercise AQ | - |  |  | 5.24 | (1.83, 8.65) |
| Mixed exercise AQ | - |  |  | 0.79 | (-2.06, 3.63) |
| Weight loss | - |  |  | -0.17 | (-3.51, 3.16) |
| Neuromodulation | - |  |  | 0.73 | (-1.94, 3.36) |
| Non-mainstream practice | - |  |  | -0.17 | (-2.96, 2.56) |
| Dental splint | 1 | -0.63 | (-1.15, -0.10) | -0.64 | (-2.89, 1.63) |
| HBOT | - |  |  | -3.52 | (-7.25, 0.18) |
| Aerobic exercise LD + Flexibility exercise LD | - |  |  | -3.71 | (-7.89, 0.37) |
| Multidisciplinary | - |  |  | 2.78 | (-0.57, 6.07) |
| Flexibility exercise LD + Manual Therapy | - |  |  | 1.76 | (-2.10, 5.63) |
| Balneotherapy + Mixed exercise AQ | - |  |  | 1.34 | (-2.08, 4.75) |
| Tricyclics | - |  |  | -0.28 | (-4.16, 3.65) |
| Antipsychotics | - |  |  | -0.31 | (-3.52, 2.94) |
| Endogenous hormones | - |  |  | 1.22 | (-2.04, 4.44) |
| Antioxidant | - |  |  | 0.69 | (-2.56, 3.97) |
| SRI | - |  |  | 0.97 | (-1.60, 3.52) |
| Gabapentinoid | - |  |  | 0.56 | (-1.96, 3.05) |
| Analgesic | - |  |  | 0.74 | (-2.44, 3.92) |
| CNS depressants | - |  |  | 0.79 | (-1.85, 3.45) |
| Strengthening exercise LD | - |  |  | 0.02 | (-3.72, 3.83) |

NMA Network meta-analysis; SMD Standardised mean difference; CI Confidence interval; Crl Credible interval. Negative values indicate a better outcome while higher values indicate a worse outcome.

**Table 15. Results for direct and NMA compared to Flexibility exercise LD**

|  | **Direct evidence** | | | **NMA** | |
| --- | --- | --- | --- | --- | --- |
| **Intervention** | **Number of trials** | **SMD** | **95% CI** | **SMD** | **95% Crl** |
| PT/BT sleep | - |  |  | -1.39 | (-3.63, 0.85) |
| Mind-body exercise AQ | - |  |  | 3.76 | (0.53, 6.99) |
| Mixed exercise AQ | - |  |  | -0.69 | (-3.10, 1.68) |
| Weight loss | - |  |  | -1.65 | (-4.55, 1.29) |
| Neuromodulation | - |  |  | -0.75 | (-3.20, 1.70) |
| Non-mainstream practice | - |  |  | -1.65 | (-4.12, 0.81) |
| Dental splint | - |  |  | -2.11 | (-5.97, 1.74) |
| HBOT | - |  |  | -5.01 | (-8.39, -1.62) |
| Aerobic exercise LD + Flexibility exercise LD | - |  |  | -5.20 | (-9.00, -1.45) |
| Multidisciplinary | - |  |  | 1.30 | (-1.62, 4.23) |
| Flexibility exercise LD + Manual Therapy | 1 | 0.29 | (-0.37, 0.95) | 0.28 | (-2.00, 2.56) |
| Balneotherapy + Mixed exercise AQ | - |  |  | -0.12 | (-3.17, 2.91) |
| Tricyclics | - |  |  | -1.74 | (-5.57, 2.03) |
| Antipsychotics | - |  |  | -1.77 | (-4.86, 1.29) |
| Endogenous hormones | - |  |  | -0.26 | (-3.35, 2.86) |
| Antioxidant | - |  |  | -0.78 | (-3.94, 2.31) |
| SRI | - |  |  | -0.51 | (-2.85, 1.84) |
| Gabapentinoid | - |  |  | -0.91 | (-3.21, 1.36) |
| Analgesic | - |  |  | -0.74 | (-3.79, 2.31) |
| CNS depressants | - |  |  | -0.68 | (-3.14, 1.77) |
| Strengthening exercise LD | - |  |  | -1.46 | (-4.96, 2.06) |

NMA Network meta-analysis; SMD Standardised mean difference; CI Confidence interval; Crl Credible interval. Negative values indicate a better outcome while higher values indicate a worse outcome.

**Table 16. Results for direct and NMA compared to PT/BT sleep**

|  | **Direct evidence** | | | **NMA** | |
| --- | --- | --- | --- | --- | --- |
| **Intervention** | **Number of trials** | **SMD** | **95% CI** | **SMD** | **95% Crl** |
| Mind-body exercise AQ | - |  |  | 5.15 | (2.25, 8.05) |
| Mixed exercise AQ | - |  |  | 0.69 | (-1.11, 2.53) |
| Weight loss | - |  |  | -0.26 | (-2.83, 2.31) |
| Neuromodulation | - |  |  | 0.64 | (-1.33, 2.61) |
| Non-mainstream practice | - |  |  | -0.26 | (-2.30, 1.74) |
| Dental splint | - |  |  | -0.73 | (-4.28, 2.86) |
| HBOT | - |  |  | -3.62 | (-6.67, -0.52) |
| Aerobic exercise LD + Flexibility exercise LD | - |  |  | -3.81 | (-7.34, -0.32) |
| Multidisciplinary | - |  |  | 2.69 | (0.10, 5.27) |
| Flexibility exercise LD + Manual Therapy | - |  |  | 1.67 | (-1.51, 4.88) |
| Balneotherapy + Mixed exercise AQ | - |  |  | 1.27 | (-1.39, 3.86) |
| Tricyclics | - |  |  | -0.37 | (-3.88, 3.18) |
| Antipsychotics | - |  |  | -0.40 | (-3.11, 2.33) |
| Endogenous hormones | - |  |  | 1.13 | (-1.61, 3.87) |
| Antioxidant | - |  |  | 0.60 | (-2.16, 3.37) |
| SRI | - |  |  | 0.88 | (-0.99, 2.75) |
| Gabapentinoid | - |  |  | 0.47 | (-1.31, 2.27) |
| Analgesic | - |  |  | 0.66 | (-2.03, 3.28) |
| CNS depressants | - |  |  | 0.70 | (-1.28, 2.69) |
| Strengthening exercise LD | - |  |  | -0.06 | (-3.27, 3.17) |

NMA Network meta-analysis; SMD Standardised mean difference; CI Confidence interval; Crl Credible interval. Negative values indicate a better outcome while higher values indicate a worse outcome.

**Table 17. Results for direct and NMA compared to Mind-body exercise AQ**

|  | **Direct evidence** | | | **NMA** | |
| --- | --- | --- | --- | --- | --- |
| **Intervention** | **Number of trials** | **SMD** | **95% CI** | **SMD** | **95% Crl** |
| Mixed exercise AQ | - |  |  | -4.46 | (-7.52, -1.43) |
| Weight loss | - |  |  | -5.40 | (-8.89, -1.96) |
| Neuromodulation | - |  |  | -4.51 | (-7.33, -1.70) |
| Non-mainstream practice | - |  |  | -5.42 | (-8.33, -2.51) |
| Dental splint | - |  |  | -5.87 | (-9.97, -1.74) |
| HBOT | - |  |  | -8.77 | (-12.64, -4.91) |
| Aerobic exercise LD + Flexibility exercise LD | - |  |  | -8.96 | (-13.19, -4.71) |
| Multidisciplinary | - |  |  | -2.46 | (-5.90, 0.97) |
| Flexibility exercise LD + Manual Therapy | - |  |  | -3.47 | (-7.45, 0.45) |
| Balneotherapy + Mixed exercise AQ | - |  |  | -3.88 | (-7.45, -0.33) |
| Tricyclics | - |  |  | -5.51 | (-9.60, -1.50) |
| Antipsychotics | - |  |  | -5.54 | (-8.88, -2.17) |
| Endogenous hormones | - |  |  | -4.02 | (-7.41, -0.64) |
| Antioxidant | - |  |  | -4.56 | (-7.96, -1.14) |
| SRI | - |  |  | -4.27 | (-6.98, -1.53) |
| Gabapentinoid | - |  |  | -4.67 | (-7.36, -2.01) |
| Analgesic | - |  |  | -4.50 | (-7.87, -1.18) |
| CNS depressants | - |  |  | -4.45 | (-7.26, -1.62) |
| Strengthening exercise LD | - |  |  | -5.22 | (-9.04, -1.35) |

NMA Network meta-analysis SMD Standardised mean difference; CI Confidence interval; Crl Credible interval. Negative values indicate a better outcome while higher values indicate a worse outcome.

**Table 18. Results for direct and NMA compared to Mixed exercise AQ**

|  | **Direct evidence** | | | **NMA** | |
| --- | --- | --- | --- | --- | --- |
| **Intervention** | **Number of trials** | **SMD** | **95% CI** | **SMD** | **95% Crl** |
| Weight loss | - |  |  | -0.95 | (-3.65, 1.74) |
| Neuromodulation | - |  |  | -0.05 | (-2.20, 2.10) |
| Non-mainstream practice | - |  |  | -0.96 | (-3.15, 1.19) |
| Dental splint | - |  |  | -1.42 | (-5.08, 2.25) |
| HBOT | - |  |  | -4.32 | (-7.47, -1.12) |
| Aerobic exercise LD + Flexibility exercise LD | - |  |  | -4.50 | (-8.12, -0.87) |
| Multidisciplinary | - |  |  | 2.00 | (-0.73, 4.69) |
| Flexibility exercise LD + Manual Therapy | - |  |  | 0.97 | (-2.31, 4.28) |
| Balneotherapy + Mixed exercise AQ | - |  |  | 0.57 | (-1.58, 2.68) |
| Tricyclics | - |  |  | -1.06 | (-4.69, 2.60) |
| Antipsychotics | - |  |  | -1.09 | (-3.92, 1.73) |
| Endogenous hormones | - |  |  | 0.43 | (-2.43, 3.28) |
| Antioxidant | - |  |  | -0.09 | (-3.00, 2.82) |
| SRI | - |  |  | 0.18 | (-1.86, 2.23) |
| Gabapentinoid | - |  |  | -0.22 | (-2.22, 1.75) |
| Analgesic | - |  |  | -0.04 | (-2.84, 2.75) |
| CNS depressants | - |  |  | 0.01 | (-2.13, 2.19) |
| Strengthening exercise LD | - |  |  | -0.77 | (-4.04, 2.61) |

NMA Network meta-analysis; SMD Standardised mean difference; CI Confidence interval; Crl Credible interval. Negative values indicate a better outcome while higher values indicate a worse outcome.

**Table 19. Results for direct and NMA compared to Weight loss**

|  | **Direct evidence** | | | **NMA** | |
| --- | --- | --- | --- | --- | --- |
| **Intervention** | **Number of trials** | **SMD** | **95% CI** | **SMD** | **95% Crl** |
| Neuromodulation | - |  |  | 0.90 | (-1.87, 3.64) |
| Non-mainstream practice | - |  |  | -0.00 | (-2.80, 2.74) |
| Dental splint | - |  |  | -0.47 | (-4.50, 3.61) |
| HBOT | - |  |  | -3.35 | (-6.95, 0.19) |
| Aerobic exercise LD + Flexibility exercise LD | - |  |  | -3.56 | (-7.60, 0.47) |
| Multidisciplinary | - |  |  | 2.95 | (-0.22, 6.10) |
| Flexibility exercise LD + Manual Therapy | - |  |  | 1.94 | (-1.74, 5.63) |
| Balneotherapy + Mixed exercise AQ | - |  |  | 1.52 | (-1.73, 4.82) |
| Tricyclics | - |  |  | -0.10 | (-4.12, 3.88) |
| Antipsychotics | - |  |  | -0.13 | (-3.46, 3.15) |
| Endogenous hormones | - |  |  | 1.39 | (-1.92, 4.71) |
| Antioxidant | - |  |  | 0.86 | (-2.51, 4.18) |
| SRI | - |  |  | 1.14 | (-1.50, 3.79) |
| Gabapentinoid | - |  |  | 0.74 | (-1.90, 3.33) |
| Analgesic | - |  |  | 0.90 | (-2.35, 4.15) |
| CNS depressants | - |  |  | 0.97 | (-1.79, 3.70) |
| Strengthening exercise LD | - |  |  | 0.20 | (-3.55, 3.92) |

NMA Network meta-analysis; SMD Standardised mean difference; CI Confidence interval; Crl Credible interval. Negative values indicate a better outcome while higher values indicate a worse outcome.

**Table 20. Results for direct and NMA compared to Neuromodulation**

|  | **Direct evidence** | | | **NMA** | |
| --- | --- | --- | --- | --- | --- |
| **Intervention** | **Number of trials** | **SMD** | **95% CI** | **SMD** | **95% Crl** |
| Non-mainstream practice | - |  |  | -0.90 | (-2.90, 1.07) |
| Dental splint | - |  |  | -1.35 | (-4.85, 2.17) |
| HBOT | - |  |  | -4.25 | (-7.49, -1.04) |
| Aerobic exercise LD + Flexibility exercise LD | - |  |  | -4.45 | (-8.14, -0.81) |
| Multidisciplinary | - |  |  | 2.05 | (-0.70, 4.81) |
| Flexibility exercise LD + Manual Therapy | - |  |  | 1.03 | (-2.32, 4.39) |
| Balneotherapy + Mixed exercise AQ | - |  |  | 0.64 | (-2.26, 3.45) |
| Tricyclics | - |  |  | -1.00 | (-4.46, 2.48) |
| Antipsychotics | - |  |  | -1.03 | (-3.65, 1.58) |
| Endogenous hormones | - |  |  | 0.49 | (-2.15, 3.13) |
| Antioxidant | - |  |  | -0.04 | (-2.73, 2.66) |
| SRI | - |  |  | 0.24 | (-1.47, 1.98) |
| Gabapentinoid | - |  |  | -0.17 | (-1.79, 1.45) |
| Analgesic | - |  |  | 0.01 | (-2.56, 2.57) |
| CNS depressants | - |  |  | 0.08 | (-1.79, 1.90) |
| Strengthening exercise LD | - |  |  | -0.69 | (-3.96, 2.58) |

NMA Network meta-analysis; SMD Standardised mean difference; CI Confidence interval; Crl Credible interval. Negative values indicate a better outcome while higher values indicate a worse outcome.

**Table 21. Results for direct and NMA compared to Non-mainstream practice**

|  | **Direct evidence** | | | **NMA** | |
| --- | --- | --- | --- | --- | --- |
| **Intervention** | **Number of trials** | **SMD** | **95% CI** | **SMD** | **95% Crl** |
| Dental splint | - |  |  | -0.45 | (-4.00, 3.14) |
| HBOT | - |  |  | -3.36 | (-6.58, -0.08) |
| Aerobic exercise LD + Flexibility exercise LD | - |  |  | -3.56 | (-7.23, 0.15) |
| Multidisciplinary | - |  |  | 2.95 | (0.21, 5.69) |
| Flexibility exercise LD + Manual Therapy | - |  |  | 1.94 | (-1.42, 5.26) |
| Balneotherapy + Mixed exercise AQ | - |  |  | 1.54 | (-1.35, 4.38) |
| Tricyclics | - |  |  | -0.10 | (-3.64, 3.43) |
| Antipsychotics | - |  |  | -0.14 | (-2.84, 2.59) |
| Endogenous hormones | - |  |  | 1.40 | (-1.34, 4.16) |
| Antioxidant | - |  |  | 0.86 | (-1.92, 3.67) |
| SRI | - |  |  | 1.14 | (-0.71, 3.02) |
| Gabapentinoid | - |  |  | 0.73 | (-1.05, 2.54) |
| Analgesic | - |  |  | 0.91 | (-1.75, 3.57) |
| CNS depressants | - |  |  | 0.97 | (-1.02, 2.97) |
| Strengthening exercise LD | - |  |  | 0.20 | (-3.07, 3.52) |

NMA Network meta-analysis; SMD Standardised mean difference; CI Confidence interval; Crl Credible interval. Negative values indicate a better outcome while higher values indicate a worse outcome.

**Table 22. Results for direct and NMA compared to Dental splint**

|  | **Direct evidence** | | | **NMA** | |
| --- | --- | --- | --- | --- | --- |
| **Intervention** | **Number of trials** | **SMD** | **95% CI** | **SMD** | **95% Crl** |
| HBOT | - |  |  | -2.89 | (-7.27, 1.48) |
| Aerobic exercise LD + Flexibility exercise LD | - |  |  | -3.09 | (-7.83, 1.57) |
| Multidisciplinary | - |  |  | 3.41 | (-0.63, 7.42) |
| Flexibility exercise LD + Manual Therapy | - |  |  | 2.39 | (-2.10, 6.88) |
| Balneotherapy + Mixed exercise AQ | - |  |  | 1.97 | (-2.09, 6.08) |
| Tricyclics | - |  |  | 0.36 | (-4.15, 4.93) |
| Antipsychotics | - |  |  | 0.31 | (-3.62, 4.32) |
| Endogenous hormones | - |  |  | 1.83 | (-2.08, 5.83) |
| Antioxidant | - |  |  | 1.32 | (-2.66, 5.30) |
| SRI | - |  |  | 1.60 | (-1.80, 5.01) |
| Gabapentinoid | - |  |  | 1.18 | (-2.21, 4.58) |
| Analgesic | - |  |  | 1.37 | (-2.54, 5.26) |
| CNS depressants | - |  |  | 1.43 | (-2.11, 4.94) |
| Strengthening exercise LD | - |  |  | 0.65 | (-3.70, 5.04) |

NMA Network meta-analysis; SMD Standardised mean difference; CI Confidence interval; Crl Credible interval. Negative values indicate a better outcome while higher values indicate a worse outcome.

**Table 23. Results for direct and NMA compared to HBOT**

|  | **Direct evidence** | | | **NMA** | |
| --- | --- | --- | --- | --- | --- |
| **Intervention** | **Number of trials** | **SMD** | **95% CI** | **SMD** | **95% Crl** |
| Aerobic exercise LD + Flexibility exercise LD | - |  |  | -0.20 | (-4.52, 4.16) |
| Multidisciplinary | - |  |  | 6.31 | (2.71, 9.88) |
| Flexibility exercise LD + Manual Therapy | - |  |  | 5.29 | (1.23, 9.34) |
| Balneotherapy + Mixed exercise AQ | - |  |  | 4.89 | (1.21, 8.52) |
| Tricyclics | - |  |  | 3.27 | (-1.08, 7.55) |
| Antipsychotics | - |  |  | 3.22 | (-0.50, 6.93) |
| Endogenous hormones | - |  |  | 4.74 | (0.99, 8.50) |
| Antioxidant | - |  |  | 4.22 | (0.49, 7.96) |
| SRI | - |  |  | 4.50 | (1.32, 7.63) |
| Gabapentinoid | - |  |  | 4.09 | (0.98, 7.17) |
| Analgesic | - |  |  | 4.26 | (0.58, 7.96) |
| CNS depressants | - |  |  | 4.33 | (1.09, 7.54) |
| Strengthening exercise LD | - |  |  | 3.55 | (-0.48, 7.65) |

NMA Network meta-analysis; SMD Standardised mean difference; CI Confidence interval; Crl Credible interval. Negative values indicate a better outcome while higher values indicate a worse outcome.

**Table 24. Results for direct and NMA compared to Aerobic exercise LD + Flexibility exercise LD**

|  | **Direct evidence** | | | **NMA** | |
| --- | --- | --- | --- | --- | --- |
| **Intervention** | **Number of trials** | **SMD** | **95% CI** | **SMD** | **95% Crl** |
| Multidisciplinary | - |  |  | 6.50 | (2.49, 10.57) |
| Flexibility exercise LD + Manual Therapy | - |  |  | 5.49 | (1.07, 9.92) |
| Balneotherapy + Mixed exercise AQ | - |  |  | 5.09 | (0.98, 9.11) |
| Tricyclics | - |  |  | 3.45 | (-1.23, 8.19) |
| Antipsychotics | - |  |  | 3.41 | (-0.71, 7.57) |
| Endogenous hormones | - |  |  | 4.94 | (0.84, 9.03) |
| Antioxidant | - |  |  | 4.41 | (0.26, 8.57) |
| SRI | - |  |  | 4.69 | (1.10, 8.30) |
| Gabapentinoid | - |  |  | 4.27 | (0.72, 7.87) |
| Analgesic | - |  |  | 4.46 | (0.39, 8.56) |
| CNS depressants | - |  |  | 4.53 | (0.87, 8.20) |
| Strengthening exercise LD | - |  |  | 3.74 | (-0.69, 8.20) |

NMA Network meta-analysis; SMD Standardised mean difference; CI Confidence interval; Crl Credible interval. Negative values indicate a better outcome while higher values indicate a worse outcome.

**Table 25. Results for direct and NMA compared to Multidisciplinary**

|  | **Direct evidence** | | | **NMA** | |
| --- | --- | --- | --- | --- | --- |
| **Intervention** | **Number of trials** | **SMD** | **95% CI** | **SMD** | **95% Crl** |
| Flexibility exercise LD + Manual Therapy | - |  |  | -1.02 | (-4.72, 2.70) |
| Balneotherapy + Mixed exercise AQ | - |  |  | -1.42 | (-4.72, 1.85) |
| Tricyclics | - |  |  | -3.06 | (-7.05, 0.95) |
| Antipsychotics | - |  |  | -3.10 | (-6.37, 0.20) |
| Endogenous hormones | - |  |  | -1.56 | (-4.85, 1.74) |
| Antioxidant | - |  |  | -2.09 | (-5.43, 1.25) |
| SRI | - |  |  | -1.82 | (-4.45, 0.83) |
| Gabapentinoid | - |  |  | -2.22 | (-4.82, 0.39) |
| Analgesic | - |  |  | -2.04 | (-5.31, 1.24) |
| CNS depressants | - |  |  | -1.98 | (-4.72, 0.77) |
| Strengthening exercise LD | - |  |  | -2.76 | (-6.49, 0.95) |

NMA Network meta-analysis; SMD Standardised mean difference; CI Confidence interval; Crl Credible interval. Negative values indicate a better outcome while higher values indicate a worse outcome.

**Table 26. Results for direct and NMA compared to Flexibility exercise LD + Manual Therapy**

|  | **Direct evidence** | | | **NMA** | |
| --- | --- | --- | --- | --- | --- |
| **Intervention** | **Number of trials** | **SMD** | **95% CI** | **SMD** | **95% Crl** |
| Balneotherapy + Mixed exercise AQ | - |  |  | -0.40 | (-4.19, 3.36) |
| Tricyclics | - |  |  | -2.04 | (-6.45, 2.42) |
| Antipsychotics | - |  |  | -2.06 | (-5.88, 1.79) |
| Endogenous hormones | - |  |  | -0.54 | (-4.37, 3.28) |
| Antioxidant | - |  |  | -1.06 | (-4.93, 2.80) |
| SRI | - |  |  | -0.79 | (-4.07, 2.47) |
| Gabapentinoid | - |  |  | -1.20 | (-4.42, 2.02) |
| Analgesic | - |  |  | -1.01 | (-4.79, 2.80) |
| CNS depressants | - |  |  | -0.97 | (-4.32, 2.40) |
| Strengthening exercise LD | - |  |  | -1.74 | (-5.93, 2.44) |

NMA Network meta-analysis; SMD Standardised mean difference; CI Confidence interval; Crl Credible interval. Negative values indicate a better outcome while higher values indicate a worse outcome.

**Table 27. Results for direct and NMA compared to Balneotherapy + Mixed exercise AQ**

|  | **Direct evidence** | | | **NMA** | |
| --- | --- | --- | --- | --- | --- |
| **Intervention** | **Number of trials** | **SMD** | **95% CI** | **SMD** | **95% Crl** |
| Tricyclics | - |  |  | -1.64 | (-5.64, 2.40) |
| Antipsychotics | - |  |  | -1.66 | (-5.01, 1.76) |
| Endogenous hormones | - |  |  | -0.15 | (-3.52, 3.25) |
| Antioxidant | - |  |  | -0.67 | (-4.09, 2.80) |
| SRI | - |  |  | -0.39 | (-3.15, 2.40) |
| Gabapentinoid | - |  |  | -0.80 | (-3.52, 1.93) |
| Analgesic | - |  |  | -0.62 | (-3.96, 2.74) |
| CNS depressants | - |  |  | -0.56 | (-3.39, 2.30) |
| Strengthening exercise LD | - |  |  | -1.33 | (-5.05, 2.46) |

NMA Network meta-analysis; SMD Standardised mean difference; CI Confidence interval; Crl Credible interval. Negative values indicate a better outcome while higher values indicate a worse outcome.

**Table 28. Results for direct and NMA compared to Tricyclics**

|  | **Direct evidence** | | | **NMA** | |
| --- | --- | --- | --- | --- | --- |
| **Intervention** | **Number of trials** | **SMD** | **95% CI** | **SMD** | **95% Crl** |
| Antipsychotics | 1 | -0.02 | (-0.47, 0.43) | -0.03 | (-2.28, 2.22) |
| Endogenous hormones | - |  |  | 1.50 | (-2.45, 5.44) |
| Antioxidant | - |  |  | 0.96 | (-2.97, 4.92) |
| SRI | - |  |  | 1.23 | (-2.09, 4.62) |
| Gabapentinoid | - |  |  | 0.83 | (-2.51, 4.19) |
| Analgesic | - |  |  | 1.01 | (-2.86, 4.88) |
| CNS depressants | - |  |  | 1.07 | (-2.40, 4.53) |
| Strengthening exercise LD | - |  |  | 0.29 | (-4.07, 4.63) |

NMA Network meta-analysis; SMD Standardised mean difference; CI Confidence interval; Crl Credible interval. Negative values indicate a better outcome while higher values indicate a worse outcome.

**Table 29. Results for direct and NMA compared to Antipsychotics**

|  | **Direct evidence** | | | **NMA** | |
| --- | --- | --- | --- | --- | --- |
| **Intervention** | **Number of trials** | **SMD** | **95% CI** | **SMD** | **95% Crl** |
| Endogenous hormones | - |  |  | 1.53 | (-1.70, 4.76) |
| Antioxidant | - |  |  | 0.99 | (-2.27, 4.25) |
| SRI | - |  |  | 1.27 | (-1.24, 3.82) |
| Gabapentinoid | - |  |  | 0.86 | (-1.60, 3.34) |
| Analgesic | - |  |  | 1.05 | (-2.12, 4.21) |
| CNS depressants | - |  |  | 1.10 | (-1.54, 3.71) |
| Strengthening exercise LD | - |  |  | 0.34 | (-3.41, 4.00) |

NMA Network meta-analysis; SMD Standardised mean difference; CI Confidence interval; Crl Credible interval. Negative values indicate a better outcome while higher values indicate a worse outcome

**Table 30. Results for direct and NMA compared to Endogenous hormones**

|  | **Direct evidence** | | | **NMA** | |
| --- | --- | --- | --- | --- | --- |
| **Intervention** | **Number of trials** | **SMD** | **95% CI** | **SMD** | **95% Crl** |
| Antioxidant | - |  |  | -0.53 | (-3.78, 2.72) |
| SRI | - |  |  | -0.25 | (-2.79, 2.32) |
| Gabapentinoid | - |  |  | -0.66 | (-3.14, 1.85) |
| Analgesic | - |  |  | -0.48 | (-3.66, 2.69) |
| CNS depressants | - |  |  | -0.42 | (-3.07, 2.22) |
| Strengthening exercise LD | - |  |  | -1.19 | (-4.93, 2.57) |

NMA Network meta-analysis; SMD Standardised mean difference; CI Confidence interval; Crl Credible interval. Negative values indicate a better outcome while higher values indicate a worse outcome.

**Table 31.** **Results for direct and NMA compared to Antioxidant**

|  | **Direct evidence** | | | **NMA** | |
| --- | --- | --- | --- | --- | --- |
| **Intervention** | **Number of trials** | **SMD** | **95% CI** | **SMD** | **95% Crl** |
| SRI | - |  |  | 0.27 | (-2.30, 2.87) |
| Gabapentinoid | - |  |  | -0.14 | (-2.66, 2.39) |
| Analgesic | - |  |  | 0.05 | (-3.15, 3.27) |
| CNS depressants | - |  |  | 0.11 | (-2.58, 2.81) |
| Strengthening exercise LD | - |  |  | -0.67 | (-4.42, 3.15) |

NMA Network meta-analysis; SMD Standardised mean difference; CI Confidence interval; Crl Credible interval. Negative values indicate a better outcome while higher values indicate a worse outcome.

**Table 32. Results for direct and NMA compared to SRI**

|  | **Direct evidence** | | | **NMA** | |
| --- | --- | --- | --- | --- | --- |
| **Intervention** | **Number of trials** | **SMD** | **95% CI** | **SMD** | **95% Crl** |
| Gabapentinoid | - |  |  | -0.41 | (-1.88, 1.07) |
| Analgesic | - |  |  | -0.22 | (-2.69, 2.21) |
| CNS depressants | - |  |  | -0.17 | (-1.89, 1.56) |
| Strengthening exercise LD | - |  |  | -0.95 | (-4.08, 2.23) |

NMA Network meta-analysis; SMD Standardised mean difference; CI Confidence interval; Crl Credible interval. Negative values indicate a better outcome while higher values indicate a worse outcome.

**Table 33. Results for direct and NMA compared to Gabapentinoid**

|  | **Direct evidence** | | | **NMA** | |
| --- | --- | --- | --- | --- | --- |
| **Intervention** | **Number of trials** | **SMD** | **95% CI** | **SMD** | **95% Crl** |
| Analgesic | - |  |  | 0.18 | (-2.24, 2.59) |
| CNS depressants | - |  |  | 0.24 | (-1.41, 1.88) |
| Strengthening exercise LD | - |  |  | -0.53 | (-3.63, 2.61) |

NMA Network meta-analysis; SMD Standardised mean difference; CI Confidence interval; Crl Credible interval. Negative values indicate a better outcome while higher values indicate a worse outcome.

**Table 34. Results for direct and NMA compared to Analgesic**

|  | **Direct evidence** | | | **NMA** | |
| --- | --- | --- | --- | --- | --- |
| **Intervention** | **Number of trials** | **SMD** | **95% CI** | **SMD** | **95% Crl** |
| CNS depressants | - |  |  | 0.05 | (-2.48, 2.64) |
| Strengthening exercise LD | - |  |  | -0.70 | (-4.37, 2.98) |

NMA Network meta-analysis; SMD Standardised mean difference; CI Confidence interval; Crl Credible interval. Negative values indicate a better outcome while higher values indicate a worse outcome.

**Table 35. Results for direct and NMA compared to CNS depressants**

|  | **Direct evidence** | | | **NMA** | |
| --- | --- | --- | --- | --- | --- |
| **Intervention** | **Number of trials** | **SMD** | **95% CI** | **SMD** | **95% Crl** |
| Strengthening exercise LD | - |  |  | -0.76 | (-4.01, 2.50) |

NMA Network meta-analysis; SMD Standardised mean difference; CI Confidence interval; Crl Credible interval. Negative values indicate a better outcome while higher values indicate a worse outcome.
