# Supplementary material for: Effects of Pharmacologic and Nonpharmacologic Interventions for the Management of Sleep Problems in People With Fibromyalgia: Systematic Review and Network Meta‐Analysis of Randomized Controlled Trials
Source: Arthritis Care Res (Hoboken). 2025 Mar 26;77(9):1095–105. doi: 10.1002/acr.25505 (PMC12371313; doi:10.1002/acr.25505)
Supplement: Supplementary file 5 — Appendix 4: Network‐meta‐analysis results for other outcomes [file ACR-77-1095-s001.docx]

**Appendix 4 Network-meta-analysis results for other outcomes**

**Table 1. Interventions and the number of participants**

| **Intervention** | **FIQ** | **SF-36 MCS** | **SF-36 PCS** | **Sleep duration** |
| --- | --- | --- | --- | --- |
| Placebo/Sham | 2263 | 1167 | 1167 | 179 |
| Education + Flexibility exercise LD | 33 | 33 | 33 |  |
| Mind-body exercise LD | 420 | 281 | 281 |  |
| Aerobic exercise LD | 107 | 75 | 75 |  |
| Education | 182 | 22 | 22 |  |
| Flexibility exercise AQ | 39 |  |  |  |
| Usual care | 559 | 153 | 153 |  |
| Aerobic exercise AQ | 59 |  |  |  |
| Nutrition | 73 | 36 | 36 |  |
| Balneotherapy | 37 |  |  |  |
| PT/BT generic | 145 |  |  |  |
| Manual therapy | 45 |  |  |  |
| Relaxation | 67 |  |  |  |
| Electrotherapy | 20 | 20 | 20 |  |
| Flexibility exercise LD | 57 |  |  |  |
| PT/BT sleep | 77 |  |  |  |
| Mind-body exercise AQ | 60 |  |  |  |
| Mixed exercise AQ | 89 |  |  |  |
| Weight loss | 41 |  |  |  |
| Neuromodulation | 76 |  |  |  |
| Non-mainstream practice | 47 | 47 | 47 |  |
| HBOT | 9 |  |  |  |
| Aerobic exercise LD + Flexibility exercise LD | 32 |  |  |  |
| PT/BT generic + Relaxation | 29 |  |  |  |
| Multidisciplinary | 81 |  |  |  |
| Flexibility exercise LD + Manual Therapy | 17 |  |  |  |
| Balneotherapy + Mixed exercise AQ | 36 |  |  |  |
| Tricyclics | 43 |  |  |  |
| Antipsychotics | 53 |  |  |  |
| Antioxidant | 12 | 12 | 12 |  |
| SRI | 573 | 556 | 556 | 15 |
| Iron replacement | 38 |  |  |  |
| Gabapentinoid | 737 | 245 | 245 | 169 |
| Analgesic | 90 |  |  |  |
| CNS depressants | 881 | 489 | 489 |  |

**Abbreviation:** FIQ Fibromyalgia Impact Questionnaire; LD, land-based; AQ, aquatic; PT/BT, psychological or behavioural therapy; HBOT Hyperbaric oxygen therapy; SRI, serotonin reuptake inhibitor; CNS Central nervous system.


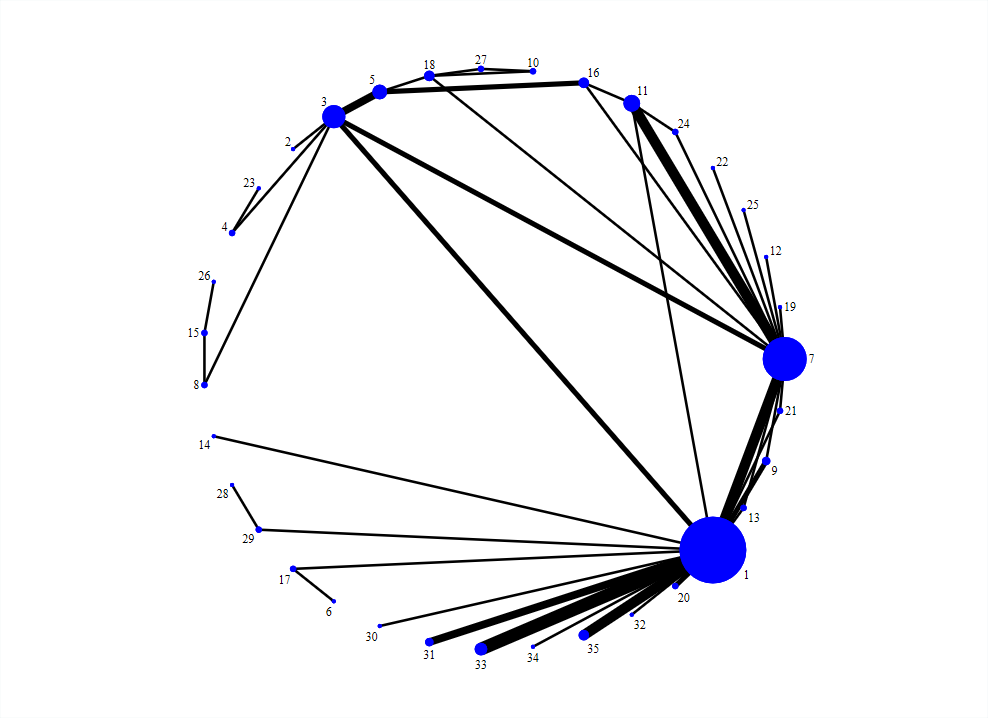


1, Placebo/sham; 2, Education + Flexibility exercise LD; 3, Mind-body exercise LD; 4, Aerobic exercise LD; 5, Education; 6, Flexibility exercise AQ; 7, Usual care; 8, Aerobic exercise AQ; 9, Nutrition; 10, Balneotherapy; 11, PT/BT generic; 12, Manual therapy; 13, Relaxation; 14, Electrotherapy; 15, Flexibility exercise LD; 16, PT/BT sleep-focused; 17, Mind-body exercise AQ; 18, Mixed exercise AQ; 19, Weight loss; 20, Neuromodulation; 21, Non-mainstream practice; 22, HBOT; 23, Aerobic exercise LD + Flexibility exercise LD; 24, PT/BT generic + Relaxation; 25, Multidisciplinary; 26, Flexibility exercise LD + Manual Therapy; 27, Balneotherapy + Mixed exercise AQ; 28, Tricyclics; 29, Antioxidant; 30, Serotonin reuptake inhibitors (SRI); 31, Iron replacement; 32, Gabapentinoid; 33, Analgesic; 34, Antipsychotics; 35, Central nervous system (CNS) depressants;

**Abbreviation:** LD, land-based; AQ, aquatic; PT/BT, psychological or behavioural therapy.

**Note:** Circle size represents the number of randomised participants; line width represents the number of direct comparisons.

**Figure 1. Network diagram for** **Fibromyalgia Impact Questionnaire**


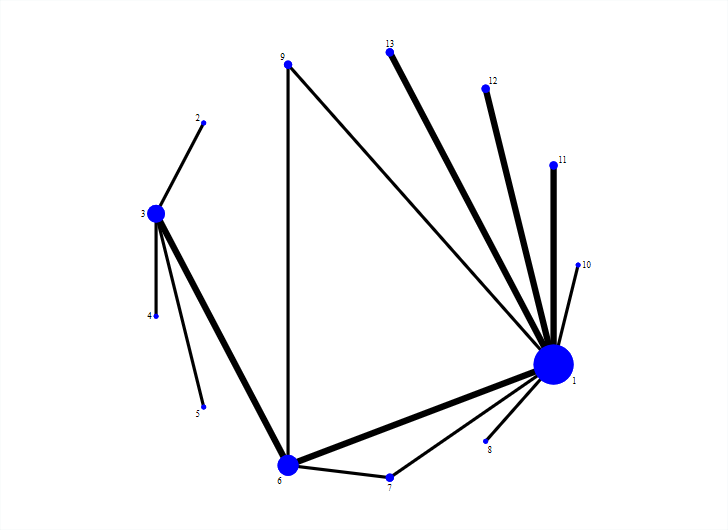


1, Placebo/sham; 2, Education + Flexibility exercise LD; 3, Mind-body exercise LD; 4, Aerobic exercise LD; 5, Education; 6, Usual care; 7, Nutrition; 8, Electrotherapy; 9, Non-mainstream practice; 10, Antioxidant; 11, Serotonin reuptake inhibitors (SRI); 12, Gabapentinoid; 13, Central nervous system (CNS) depressants.

**Abbreviation:** LD, land-based;

**Note:** Circle size represents the number of randomised participants; line width represents the number of direct comparisons

**Figure 2 Network diagram for SF-36 mental component summary score**


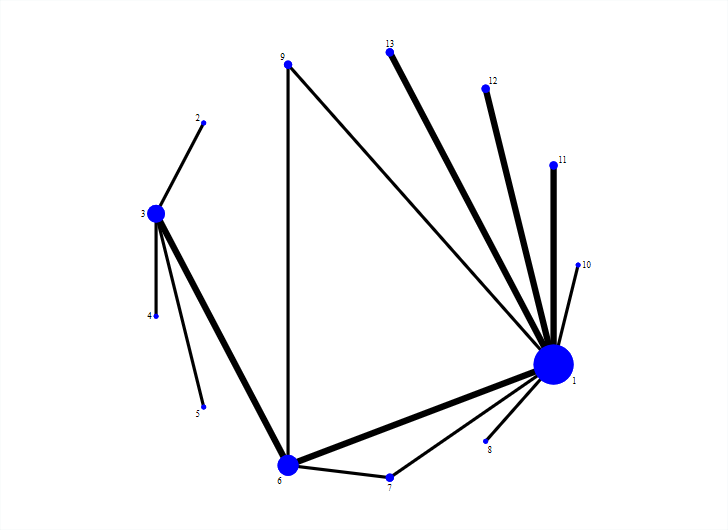


1, Placebo/sham; 2, Education + Flexibility exercise LD; 3, Mind-body exercise LD; 4, Aerobic exercise LD; 5, Education; 6, Usual care; 7, Nutrition; 8, Electrotherapy; 9, Non-mainstream practice; 10, Antioxidant; 11, Serotonin reuptake inhibitors (SRI); 12, Gabapentinoid; 13, Central nervous system (CNS) depressants.

**Abbreviation:** LD, land-based;

**Note:** Circle size represents the number of randomised participants; line width represents the number of direct comparisons

**Figure 3 Network diagram for SF-36 mental component summary score**


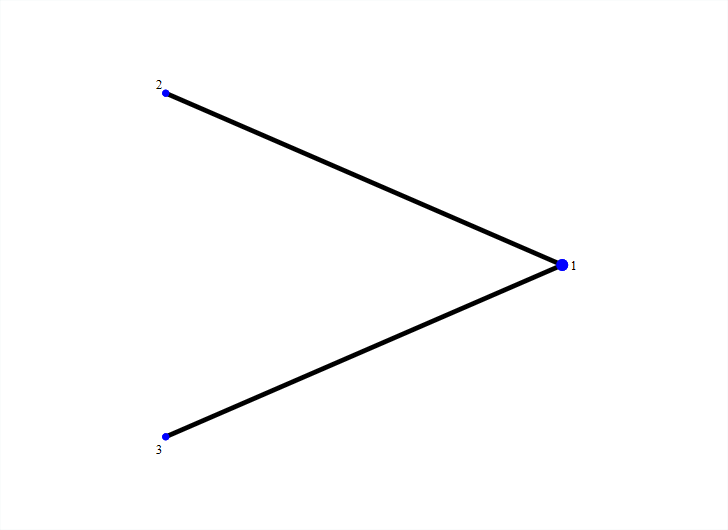


1, Placebo; 2, Serotonin reuptake inhibitors (SRI); 3, Gabapentinoid

**Figure 4. Network diagram for sleep duration**

**Table 2. Direct evidence compared to placebo/sham**

| **Intervention** | **FIQ** | **SF-36 MCS** | **SF-36 PCS** | **Sleep duration** |
| --- | --- | --- | --- | --- |
|  | **MD (95% CI)** | **MD (95% CI)** | **MD (95% CI)** |  |
| Education + Flexibility exercise LD |  |  |  |  |
| Mind-body exercise LD | -19.49 (-29.14, -9.84) |  |  |  |
| Aerobic exercise LD |  |  |  |  |
| Education |  |  |  |  |
| Flexibility exercise AQ |  |  |  |  |
| Usual care | 0.23 (-0.48, 0.93) | -0.39 (-5.34, 4.57) | 0.62 (-2.68, 3.91) |  |
| Aerobic exercise AQ |  |  |  |  |
| Nutrition | -5.77 (-12.34, 0.80) | -7.79 (-16.17, 0.59) | 0.83 (-4.71, 6.37) |  |
| Balneotherapy |  |  |  |  |
| PT/BT generic | -0.18 (-0.93, 0.57) |  |  |  |
| Manual therapy |  |  |  |  |
| Relaxation | 0.07 (-7.25, 7.39) |  |  |  |
| Electrotherapy | -9.30 (-18.26, -0.34) | -0.80 (-3.77, 2.17) | -0.10 (-3.23, 3.03) |  |
| Flexibility exercise LD |  |  |  |  |
| PT/BT sleep |  |  |  |  |
| Mind-body exercise AQ | 2.05 (1.70, 2.40) |  |  |  |
| Mixed exercise AQ |  |  |  |  |
| Weight loss |  |  |  |  |
| Neuromodulation | 0.10 (-11.50, 11.71) |  |  |  |
| Non-mainstream practice | -5.10 (-12.30, 2.10) | 3.70 (-1.91, 9.31) | 1.60 (-2.60, 5.80) |  |
| HBOT |  |  |  |  |
| Aerobic exercise LD + Flexibility exercise LD |  |  |  |  |
| PT/BT generic + Relaxation |  |  |  |  |
| Multidisciplinary |  |  |  |  |
| Flexibility exercise LD + Manual Therapy |  |  |  |  |
| Balneotherapy + Mixed exercise AQ |  |  |  |  |
| Tricyclics |  |  |  |  |
| Antipsychotics | -6.80 (-16.31, 2.71) | 7.20 (-0.83, 15.23) | 5.10 (0.17, 10.03) |  |
| Antioxidant | -18.20 (-33.15, -3.25) |  |  |  |
| SRI | -10.16 (-14.84, -5.47) | 1.68 (0.60, 2.75) | 1.59 (0.13, 3.06) | -24.40 (-69.96, 21.16) |
| Iron replacement | -15.30 (-27.94, -2.66) |  |  |  |
| Gabapentinoid | -3.63 (-6.37, -0.90) | 0.82 (-1.12, 2.77) | 0.12 (-1.27, 1.51) | 7.40 (-8.97, 23.77) |
| Analgesic | -3.27 (-3.78, -2.76) |  |  |  |
| CNS depressants | -9.44 (-12.38, -6.51) | 0.98 (-0.89, 2.84) | 2.95 (1.90, 4.01) |  |

Higher scores indicate a worse outcome.

Between study standard deviation 4.3 (95% Crl 3.0, 5.0)

**Abbreviation:** FIQ Fibromyalgia Impact Questionnaire; MD Mean difference; CI Confidence interval; LD, land-based; AQ, aquatic; PT/BT, psychological or behavioural therapy; HBOT Hyperbaric oxygen therapy; SRI, serotonin reuptake inhibitor; CNS Central nervous system.

**Results for direct and NMA compared to other interventions (not placebo/sham) are available on request.**
